# Kinetics of ligand exchange in solution: a quantitative mass spectrometry approach†

**DOI:** 10.1039/d3sc03342b

**Published:** 2023-08-24

**Authors:** Quentin Duez, Paul Tinnemans, Johannes A. A. W. Elemans, Jana Roithová

**Affiliations:** a Radboud University, Institute for Molecules and Materials Heyendaalseweg 135 6525 AJ Nijmegen The Netherlands j.roithova@science.ru.nl quentin.duez@ru.nl

## Abstract

Complex speciation and exchange kinetics of labile ligands are critical parameters for understanding the reactivity of metal complexes in solution. We present a novel approach to determine ligand exchange parameters based on electrospray ionization mass spectrometry (ESI-MS). The introduction of isotopically labelled ligands to a solution of metal host and unlabelled ligands allows the quantitative investigation of the solution-phase equilibria. Furthermore, ion mobility separation can target individual isomers, such as ligands bound at specific sites. As a proof of concept, we investigate the solution equilibria of labile pyridine ligands coordinated in the cavity of macrocyclic porphyrin cage complexes bearing diamagnetic or paramagnetic metal centres. The effects of solvent, porphyrin coordination sphere, transition metal, and counterion on ligand dissociation are discussed. Rate constants and activation parameters for ligand dissociation in the solution can be derived from our ESI-MS approach, thereby providing mechanistic insights that are not easily obtained from traditional solution-phase techniques.

## Abstract 2

Delayed reactant labelling can provide thermodynamic parameters (Δ*G*, Δ*H*, and Δ*S*) of solution equilibria using ESI-MS monitoring. As a proof of concept, we investigated the equilibria of pyridine ligands with macrocyclic porphyrin cage complexes.

## Graphical abstract

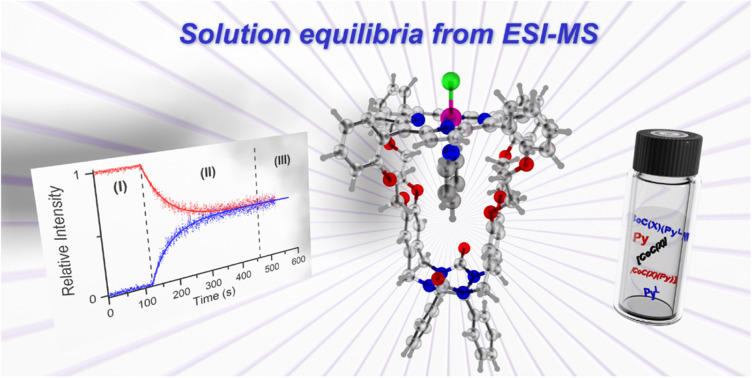

Ligands are often essential for tuning the reactivity of metal complexes in solution.^1,2^ Labile or semi-labile ligands may play a role to enhance,^3–5^ direct,^6–8^ or inhibit^9^ the reactivity of the metal centres, thereby affording more efficient and more selective catalysis. Investigating the speciation and exchange kinetics of labile ligands is thus essential to understand how metal complexes react in solution.

The binding and exchange of ligands to a metal centre are usually monitored in solution by UV-visible or nuclear magnetic resonance (NMR) spectroscopies.^10–14^ These methods provide information about the complexes' ligand exchange and spin states. However, they usually only report on the major species in solution and cannot efficiently track low-abundant complexes. Moreover, the analysis of paramagnetic complexes by NMR requires sophisticated approaches.^15^ On the contrary, mass spectrometry (MS) coupled with Electrospray Ionization (ESI) offers high sensitivity and enables to monitor minor species. It is often used to study the speciation of metal complexes with labile ligands, regardless of the nature or spin state of the metal, or to follow reactions catalysed by metal–organic complexes.^16–23^ However, a significant pitfall of the MS approaches is the difficulty of correlating the abundance of ions in the gas phase with the concentration of their precursors in the solution.^24^ Quantitative measurements, such as determining binding constants and reaction rates, thus become challenging.^25^

We designed the delayed reactant labelling (DRL) approach to bridge the gap between the solution and the gas phase experiments and to assess the artifacts related to ESI processes.^26^ This method enables the direct kinetic monitoring of an ongoing reaction with ESI-MS by introducing an isotopologue of one of the reactants after a delay. Isotopically labelled reaction intermediates are then generated from the added reactant. Assuming that the isotopically labelled and unlabelled species have the same ionization efficiency, the relative evolution of the labelled and unlabelled mass signals directly reflects the decomposition kinetics of the targeted species in solution. For instance, DRL has been used to probe the impact of solution conditions on the half-life of reactive intermediates in gold-catalysed reactions.^26^ Recently, the rate constants along the reaction paths of an enantioselective organocatalytic reaction have been mapped using DRL.^27^

Here, we employ DRL to quantitatively investigate the solution equilibria of labile pyridine ligands with macrocyclic porphyrin cage complexes bearing diamagnetic or paramagnetic metal centres (Scheme 1). These porphyrin cage complexes are designed to mimic an enzymatic binding pocket^28^ and are efficient catalysts for reactions such as alkene epoxidation.^6,29,30^

**Scheme 1:**
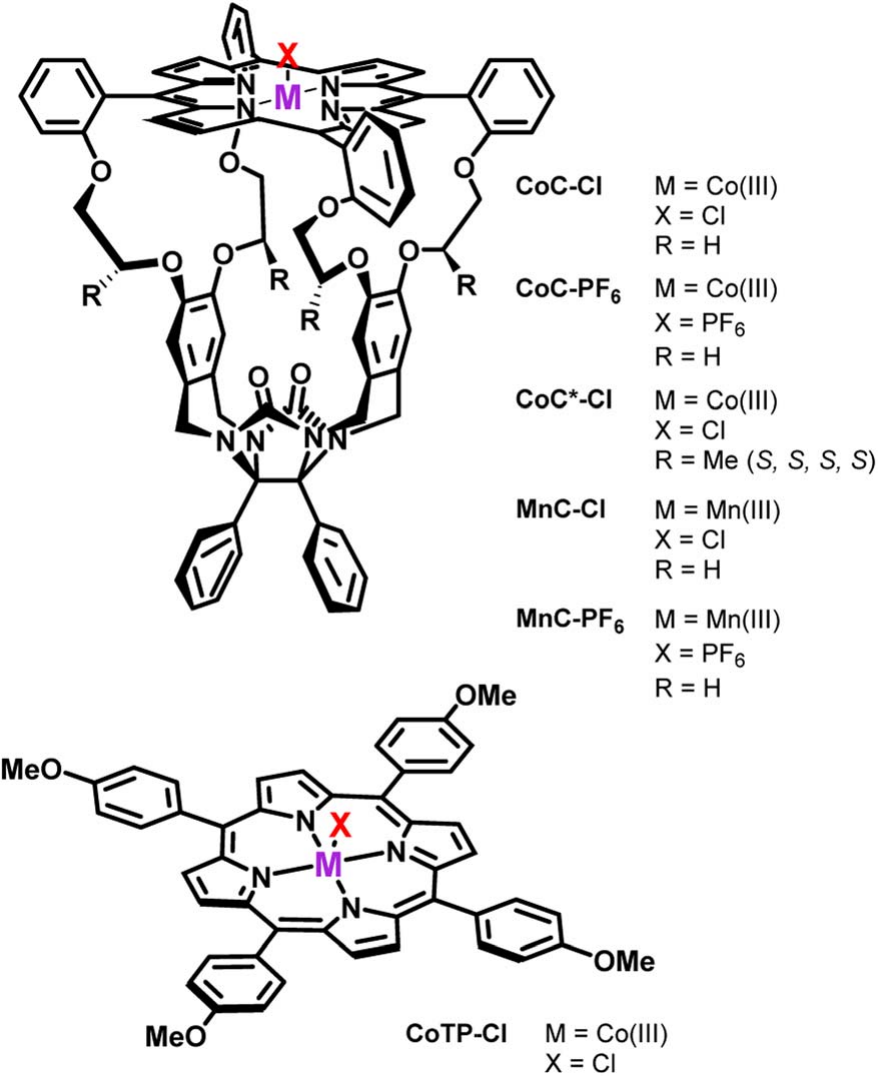
Porphyrin complexes investigated in the present work.

We show that DRL is a powerful technique for determining activation parameters for ligand dissociation and understanding exchange mechanisms in solution. We discuss the influence of solvent, porphyrin coordination sphere, transition metal, and counterion. Our methodology offers an alternative to conventional solution-phase spectroscopy techniques to obtain ligand exchange parameters for metal complexes.

## Methods

### Delayed reactant labeling (DRL) to monitor ligand exchange

**Fig. 1:**
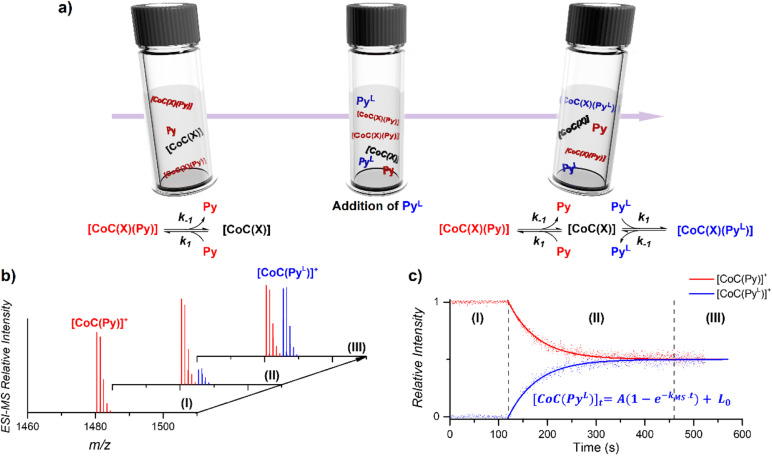
(a) Schematic representation of the DRL experiments described in this work. (I) An initial steady–state equilibrium between the porphyrin cage complex CoC(X), with X being any additional ligand that is lost during electrospray ionization (Scheme 1), and pyridine (Py) is disrupted by (II) addition of isotopically labelled pyridine (Py^L^). The concentrations of complexes bound to pyridine and isotopically labelled pyridine evolve in solution until (III) a steady state equilibrium between CoC–Cl, Py and Py^L^ is reached. (b) Evolution of the intensities of [CoC(Py)]^+^ and [CoC(Py^L^)]^+^ detected by ESI-MS. (c) Time evolution of the relative intensities of [CoC(Py)]^+^ and [CoC(Py^L^)]^+^. Dots are experimental data points and lines correspond to fittings of experimental data by first-order kinetic laws (eqn (S1) and (S2)†), thereby affording the determination of *k*_MS_.

The DRL experiment has three parts (Fig. 1). First (I), 1.25 equiv. pyridine (Py) is added to a stirred CHCl_3_ solution of the cobalt(iii) porphyrin cage complex CoC–Cl (Scheme 1) that is continuously infused in the mass spectrometer,^31,32^ leading to the detection of the complex [CoC(Py)]^+^ (*m*/*z* 1480.4) and the bis-pyridine variant [CoC(Py)_2_]^+^ (*m*/*z* 1559.4) by electrospray ionization mass spectrometry (ESI-MS).

Next (II), with a defined delay, 1.25 equiv. pyridine-D5 (Py^L^) is added to the reaction mixture. At this point, the system is set out of equilibrium, and [CoC(Py^L^)]^+^ ions (*m*/*z* 1485.4) appear in the mass spectrum (for simplicity, we discuss only the simplest complex, but it applies to all detected complexes). [CoC(Py)]^+^ and [CoC(Py^L^)]^+^ are isotopologues and have, therefore, the same ionization efficiency. The relative abundances of the ions with *m*/*z* 1480.4 and *m*/*z* 1485.4 in the mass spectrum directly report on the relative concentrations of complexes bound to pyridine and pyridine-D5. The time evolution of the relative abundances of the ions with *m*/*z* 1480.4 and *m*/*z* 1485.4 reflects the system going toward equilibrium.

Finally (III), the porphyrin cage complex reaches equilibrium with the pyridine ligands. At this point, the signal intensities of [CoC(Py)]^+^ and [CoC(Py^L^)]^+^ reflect the relative concentrations of pyridine and pyridine-D5 in the solution (the 1 : 1 ratio was selected for simplicity).

**Scheme 2:**
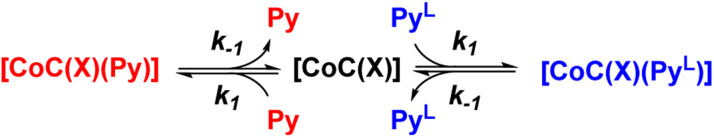
Reaction scheme used for the kinetic model (see ESI†). X stands for any additional ligand that is lost during electrospray ionization.

To understand how the relative evolution of the *m*/*z* 1480.4 and *m*/*z* 1485.4 ions (see Fig. 1c) relates to the pyridine exchange in solution, we assumed a simple reaction scheme in which we replaced an unlabelled pyridine ligand at a binding site with the labelled one (Scheme 2). Note that this scheme assumes the ligand exchange at one coordination site. The scheme does not imply the mechanism of the first step: it can be dissociative or associative if the exchange requires coordination of a *trans*-ligand.

The equations derived from the model reaction scheme can be solved analytically, assuming a significant excess of pyridine ligands (reactions have zero-order kinetics concerning the pyridine concentration, see also the kinetic model in ESI†). In such cases, the signal evolution depends only on the ligand dissociation rate constant *k*_−1_. Accordingly, fitting the DRL curves shown in Fig. 1c using first-order kinetics laws (solid lines in Fig. 1c – see also eqn (S1) and (S2) in the ESI†)^26^ directly provides *k*_−1_ when the experiment is performed with a large excess of pyridine ligands.

To evaluate which range of pyridine concentration satisfies the assumption of a large excess, we conducted DRL experiments with increasing concentrations of pyridine and pyridine-D5 and monitored the evolution of the MS-observed rate *k*_MS_. We found that the observed rate remains constant when the experiments are performed with 100 and more equivalents of pyridine(-D5) (Fig. S1†), suggesting that *k*_−1_ can be accurately measured within this concentration range. All the following experiments were thus conducted with 100 equivalents of pyridine(-D5). Interestingly, the DRL curves exhibit first-order kinetics consistently, even with a small excess of pyridine ligand (see results below).

## Results and discussion

### Binding of pyridine to the porphyrin cage CoC–Cl

**Fig. 2:**
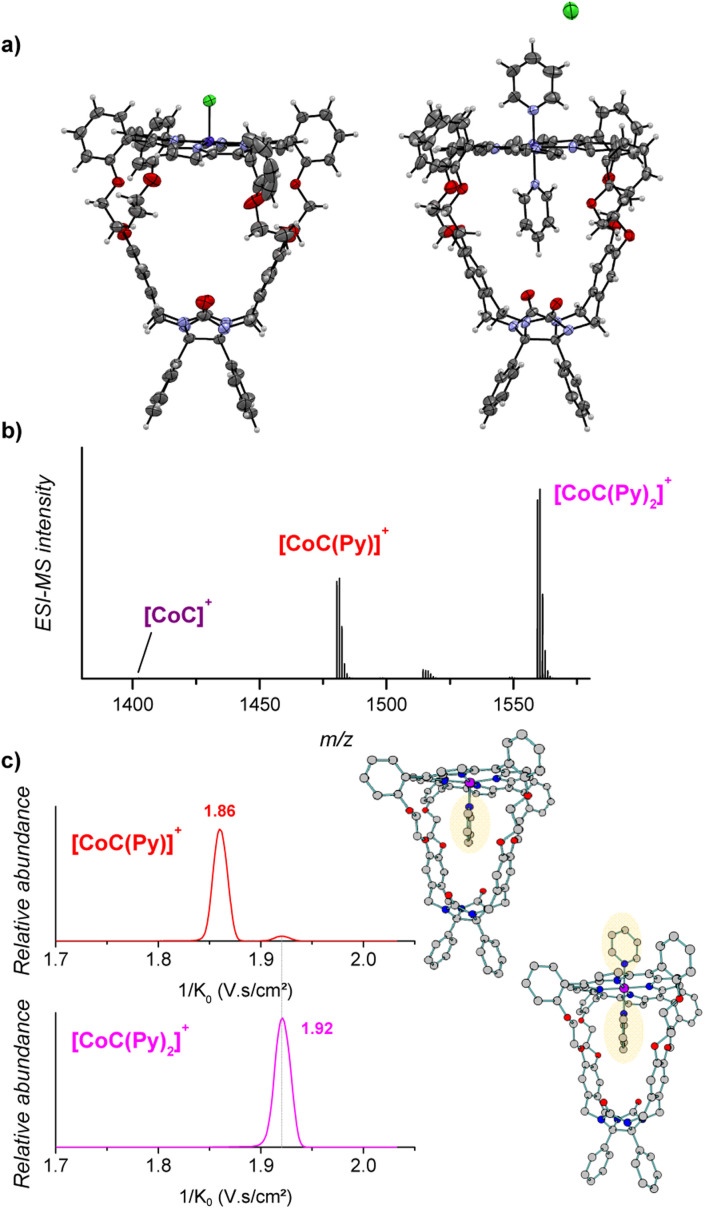
(a) X-ray structures of [CoC(Cl)] (left) and [CoC(Py)_2_]Cl (right). Color code: oxygen, red; nitrogen, blue; carbon, gray; hydrogen, white; chloride, green and cobalt, purple. (b) ESI-MS detection of [CoC]^+^ (*m*/*z* 1401.4), [CoC(Py)]^+^ (*m*/*z* 1480.4), [CoC(Py)_2_]^+^ (*m*/*z* 1559.4). Additional signals observed at *m*/*z* 1515.4 can be attributed to [CoC − H + Py + Cl]^+^. (c) Ion mobility separation of [CoC(Py)]^+^ (*m*/*z* 1480.4 – red mobilogram) and [CoC(Py)_2_]^+^ (*m*/*z* 1559.4 – pink mobilogram). DFT-calculated structures of ^1^[CoC(Py_IN_)]^+^ and ^1^[CoC(Py)_2_]^+^, optimized with B3LYP-D3/def2svp, are shown on the right side of the panel.

The porphyrin cage CoC–Cl crystalizes with the chlorido ligand at the outside of the cage cavity (Fig. 2a). This is consistent with relative energies predicted by DFT for the CoC–Cl complexes with the chloride ligand coordinated on either side of the porphyrin roof ([CoC(Cl_OUT_)] lying 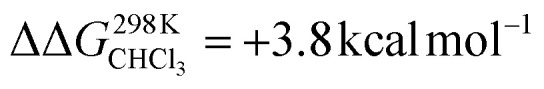 higher in energy than [CoC(Cl_IN_)]). Adding pyridine to a solution of CoC–Cl in chloroform leads to the coordination of a first pyridine ligand dominantly inside the cavity. Indeed, according to DFT calculations of the pyridine complexes with a *trans*-axial chlorido ligand, the [CoC(Py_IN_)(Cl_OUT_)] isomer represents 99.7% of the complexes in chloroform. The alternative [CoC(Py_OUT_)(Cl_IN_)] isomer lies 
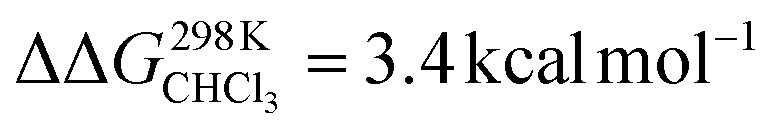
 higher in energy and represents thus only 0.3%. With an increasing molar excess of pyridine, complexes with the pyridine ligands coordinated to both sides of the porphyrin roof can be formed, as evidenced by the crystal structure of the cage complex with two pyridine ligands, [CoC(Py)_2_]^+^Cl^−^. The structure shows that the cavity has the right size to accommodate exactly one pyridine ligand, the second pyridine ligand binds at the outside, and the chloride counter ion is not coordinated (Fig. 2a).

Electrospray ionization of the solution of cobalt(iii) porphyrin cage complex CoC–Cl with 100 equiv. pyridine in CHCl_3_ leads to the detection of [CoC(Py)]^+^ (*m*/*z* 1480.4) and [CoC(Py)_2_]^+^ (*m*/*z* 1559.4) (Fig. 2b). In the 1 : 1 complexes, the pyridine ligand can, in principle, bind at both sides of the porphyrin ring, *i.e.*, inside or outside the macrocyclic pocket. In solution, the opposite coordination site would be filled by the chlorido ligand or another pyridine ligand that is eliminated during the ionization process.

The pyridine binding site in the detected [CoC(Py)]^+^ complexes can be determined by ion mobility experiments. The ion mobility separates the ions according to their shapes.^30,33–37^ The experiments reveal that the [CoC(Py)]^+^ ions exhibit two signals at 1/*K*_0_ 1.86 and 1.92 V s cm^−2^, respectively (Fig. 2c). The ions with lower 1/*K*_0_ correspond to ions with a smaller size and are thus associated with the complexes having a pyridine ligand inside the cavity: [CoC(Py_IN_)]^+^ (see Fig. S2†). The second ion population (1.92 V s cm^−2^) can have two origins: (i) complexes with pyridine bound outside the cavity ([CoC(Py_OUT_)]^+^) or (ii) fragments of the 1 : 2 complexes. The [CoC(Py)_2_]^+^ ions have only one isomer population at 1.92 V s cm^−2^, which corresponds to the same mobility as that of the second population of the [CoC(Py)]^+^ complexes. This suggests that [CoC(Py)_2_]^+^ ions could fragment in the transfer region of the mass spectrometer after the ion mobility separation, thus giving rise to the *m*/*z* of [CoC(Py)]^+^ while having the inverse mobility of [CoC(Py)_2_]^+^. The [CoC(Py_OUT_)]^+^ complexes are expected to have similar ion mobility as [CoC(Py)_2_]^+^ because the ligand inside the cavity does not significantly affect the collisional cross-section of the complex (Fig. S2†).

We tested the possible occurrence of the fragmentation in the transfer region by performing the same experiment with the [tetrakis(4-methoxyphenyl)porphyrinato] cobalt(iii) chloride (CoTP–Cl) complex (Scheme 1). The binding of a pyridine ligand on either side of the porphyrin leads to the same complex; therefore, only one ion population should be obtained by ion mobility analysis of the [CoTP(Py)]^+^ ions. ESI-MS analysis of the CoTP–Cl solution with 100 equiv. pyridine reveals the [CoTP(Py)]^+^ and [CoTP(Py)_2_]^+^ complexes (Fig. S3†). In the ion mobility spectrum the [CoTP(Py)]^+^ complexes have two ion populations, and the larger ions, *i.e.*, the ions detected at larger 1/*K*_0_ values, have the same 1/*K*_0_ value as the [CoTP(Py)_2_]^+^ complexes (Fig. S4†). This result shows that a fraction of the 1 : 2 complexes can indeed dissociate in the ion transfer region of the mass spectrometer after the mobility separation and thus contribute to the signal of the 1 : 1 complexes.

In the case of the CoC complexes, we cannot distinguish the [CoC(Py_OUT_)]^+^ isomers from the fragments. Therefore, we will analyse only the 1 : 1 complexes with pyridine inside the cavity and neglect the population at the higher 1/*K*_0_ value. Coupling ion mobility with mass detection thus enables measuring the dissociation rates of pyridine ligands vacating the cavity of the CoC cage complex, an information not easily achieved by any other technique.

### DRL experiments with small excess of pyridine ligands

DRL experiments conducted with a large excess of pyridine directly report on the ligand dissociation rate *k*_−1_. However, finding an analytical solution for the equations derived from the model reaction scheme (Scheme 2) with any excess of pyridine ligands is difficult. Nevertheless, a numerical solving with arbitrary *k*_1_ and *k*_−1_ rate constants can predict *k*_MS_ in dependence on the pyridine and pyridine-D5 concentrations.

Numerical solving of the rate equations requires estimates of the initial concentrations for the free cage, or pyridine-bound complexes when adding pyridine-D5 (see ESI†). However, these concentrations may vary if the system is not in equilibrium. To evaluate how fast the cage-pyridine system reaches equilibrium, we carried out DRL experiments for CoC–Cl with 2.5 equiv. pyridine(-D5) with different delays between adding pyridine and pyridine-D5. As shown in Fig. S5,† we did not observe any significant effect of the delay between adding pyridine and pyridine-D5 (0.5–5 min) on the MS-observed rates. This result indicates that the initial equilibrium between the cage complex and pyridine ligands is reached within 0.5 min. This is possible only with a large association rate constant (*k*_1_). We estimate the lower range for *k*_1_ to be 50 000 M^−1^ s^−1^ (see Discussion and Fig. S47 in ESI†).

**Fig. 3:**
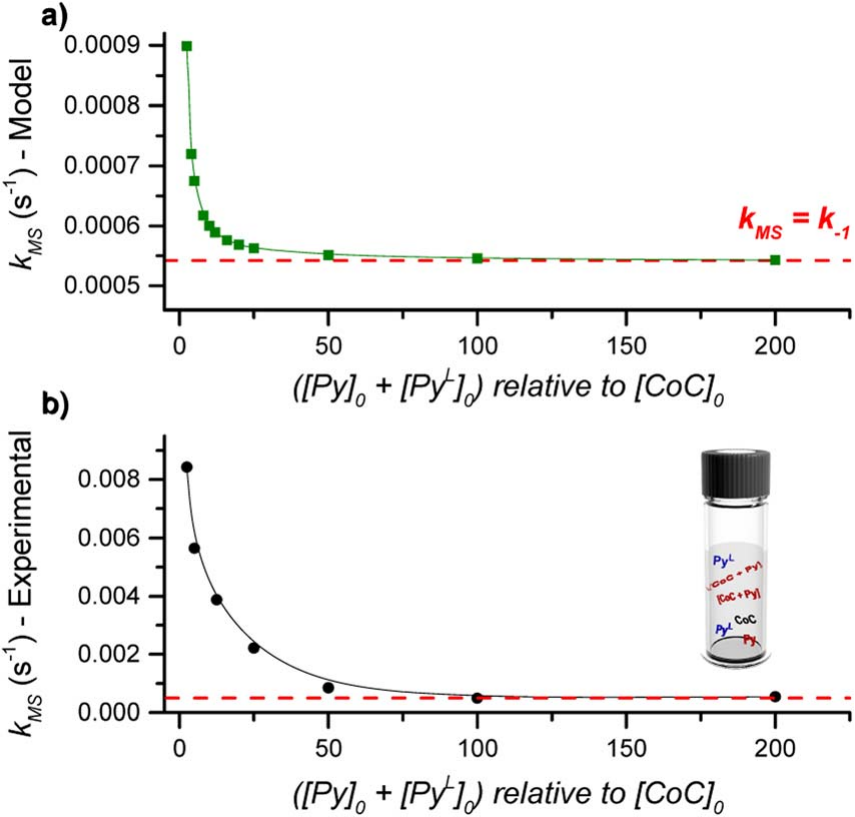
(a) Evolution of *k*_MS_ as predicted by the kinetic model with increasing initial concentrations of (pyridine + pyridine-D5). The rate constants *k*_1_ and *k*_−1_ were fixed at 50 000 M^−1^ s^−1^ and 0.00054 s^−1^, respectively. (b) Evolution of *k*_MS_ as measured by DRL experiments in CHCl_3_ at 24 °C with increasing equivalents of (pyridine + pyridine-D5). For each experiment, a 1 : 1 ratio of pyridine and pyridine-D5 was used.

Using the lower range of *k*_1_ and the dissociation rate *k*_−1_ measured by DRL as fixed values for our numerical modelling, we systematically varied the initial concentration of pyridine and pyridine-D5 and monitored the effect on *k*_MS_. Our model predicts an inverse dependence of *k*_MS_ with respect to the pyridine and pyridine-D5 concentrations (Fig. 3). In agreement with the experimental data discussed in the previous paragraphs, *k*_MS_ becomes equal to *k*_−1_ at a large excess of pyridine. However, at a small excess of pyridine, the experimental *k*_MS_ is about ten times larger than the modelled values.

This difference could originate either from (i) an incorrect estimate of *k*_1_, or from (ii) an effect of the *trans*-axial ligands, which is not taken into account in our model. We recalculated the dependence of *k*_MS_ with respect to the pyridine(-D5) concentration using different values of *k*_1_ and found that the model predicts *k*_MS_ to be independent of *k*_1_ (Fig. S51†). The difference between the experimental and modelled *k*_MS_ for a small excess of pyridine thus points to a complex mixture of cage-pyridine complexes, with either pyridine or chlorido as the *trans*-axial ligand. As discussed in the following paragraphs, the coordination of more electron-donating ligands at the outside of the cage [CoC(Py_IN_)(X_OUT_)] accelerates the dissociation of the inner pyridine ligand. This effect will be more important at small excess of pyridine ligand since more [CoC(Py_IN_)(Cl_OUT_)] complexes are expected to be present, and will thus contribute to an increase of *k*_MS_. At large excess of pyridine, the dominant speciation of cage complexes in solution proposed to be [CoC(Py)_2_]^+^, does not evolve significantly with the pyridine concentration.

### Activation parameters for pyridine exchange

**Fig. 4:**
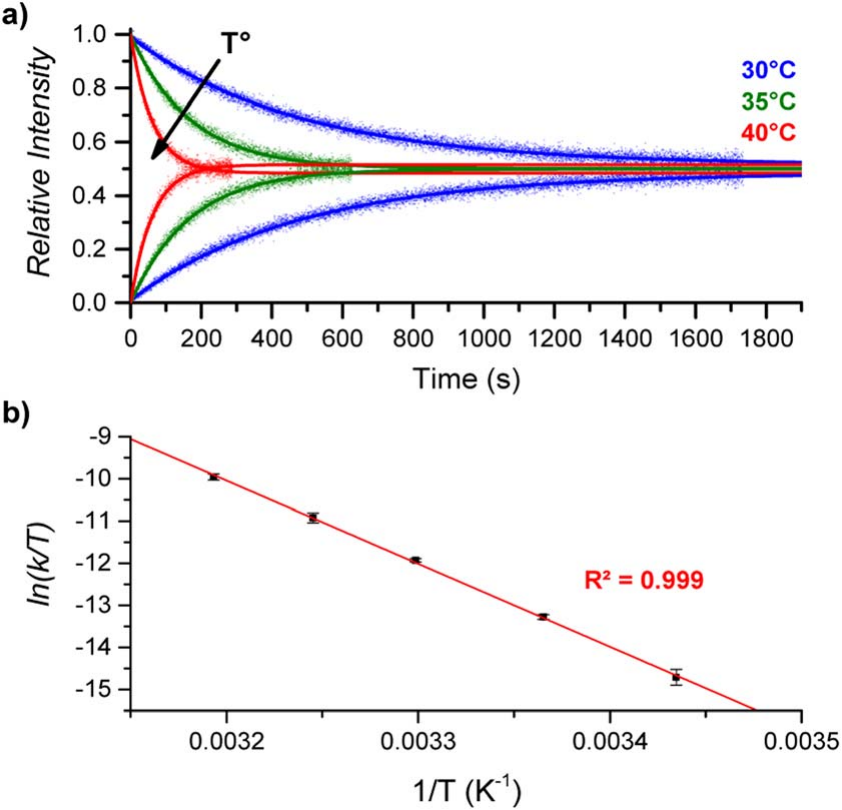
(a) Relative time evolution of [CoC(Py_IN_)]^+^ (top lines) and [CoC(Py^L^_IN_)]^+^ (bottom lines) in DRL experiments carried out in CHCl_3_ at 30 °C (blue), 35 °C (green), and 40 °C (red). Dots are experimental data points, and lines correspond to fittings of experimental data by eqn (S1) and (S2).† (b) Eyring plot built from DRL experiments carried out at different temperatures. Dots correspond to the average of ln(*k*/*T*) values from triplicate measurements, with error bars corresponding to the standard deviation.

By performing DRL experiments with a large excess (100 equiv.) of pyridine(-D5) at different temperatures (Fig. S6†), the measured rate constants *k*_−1_ can be used to build an Eyring plot, thereby enabling the determination of the activation parameters for the dissociation of 1 : 1 [CoC(Py_IN_)] complexes (Fig. 4 and S7†). As shown in Table 1 – entry 1, the dissociation of pyridine from the cavity of the [CoC(Py_IN_)]^+^ complexes is connected with an activation entropy of 58.2 cal mol^−1^ K^−1^ in CHCl_3_. Such large positive values usually correspond to a dissociative ligand exchange mechanism.^38–40^

**Table 1:** Activation parameters determined by DRL for the dissociation of 1 : 1 CoC–pyridine complexes in the presence of 100 equiv. pyridine and 100 equiv. pyridine-D5 (see rate constants in Table S1)

Entry	Host	Solvent	Δ*H*^‡^ (kcal mol^−1^)	Δ*S*^‡^ (cal mol^−1^ K^−1^)	Δ*G*^‡^ at 25 °C (kcal mol^−1^)
1	CoC–Cl	CHCl_3_	39.1 ± 0.5	58.2 ± 1.8	21.8 ± 0.8
2	CoC–Cl	CH_3_CN	28.3 ± 0.4	15.5 ± 1.2	23.7 ± 0.5
3	CoC*–Cl	CHCl_3_	42.8 ± 1.5	67.0 ± 4.9	22.8 ± 2.1
4	CoC–PF_6_	CHCl_3_	40.4 ± 0.7	59.8 ± 2.4	22.6 ± 1.0

To support the hypothesis of a dissociative process, we carried out DFT calculations to determine the energy required to remove a pyridine ligand from the cavity of the porphyrin cage. We have assumed that the complex bears either chloride or pyridine as *trans*-axial ligands on the outside of the cage in the solution [CoC(X_OUT_)(Py_IN_)]. In the case of the chlorido ligand, the free energy calculated for the dissociation of pyridine bound inside the cavity is 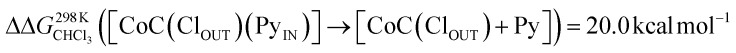 (Fig. S8†), which is in excellent agreement with the free energy of activation determined from the DRL experiments (21.8 kcal mol^−1^, Table 1 – entry 1). The enthalpy and entropy values predicted by DFT for the dissociation of pyridine bound inside the cage are Δ*H*_CHCl_3__ ([CoC(Cl_OUT_)(Py_IN_)] → [CoC(Cl_OUT_)] + Py) = 38.8 kcal mol^−1^ and Δ*S*_CHCl_3__ ([CoC(Cl_OUT_)(Py_IN_)] → [CoC(Cl_OUT_) + Py]) = 56.8 cal mol^−1^ K^−1^, which also agree with the experimental values (Table 1 – entry 1).

For the bis-pyridine complexes, the calculated free energy for the dissociation of pyridine out of the cavity is 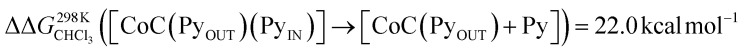 (Fig. S9†). The associated enthalpy and entropy values are calculated to be Δ*H*_CHCl_3__ ([CoC(Py_OUT_)(Py_IN_)] → [CoC(Py_OUT_) + Py]) = 42.9 kcal mol^−1^ and Δ*S*_CHCl_3__ ([CoC(Py_OUT_)(Py_IN_)] → [CoC(Py_OUT_) + Py]) = 63.4 cal mol^−1^ K^−1^, which also agree with the experimental values. Generally, a good agreement between the theoretical model and the experiment supports the hypothesis of a dissociative mechanism for pyridine exchange.

### Influence of solution conditions on ligand dissociation

#### Solvent effect

**Fig. 5:**
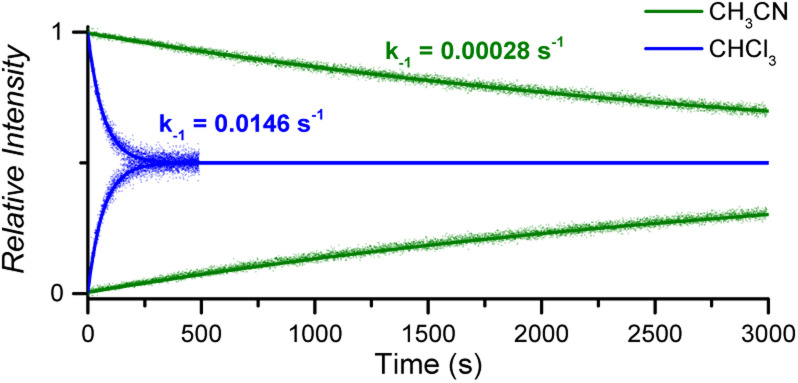
Relative time evolution of [CoC(Py_IN_)]^+^ (top traces and lines) and [CoC(Py^L^_IN_)]^+^ (bottom traces and lines) in DRL experiments carried out at 40 °C in chloroform (blue traces) and acetonitrile (green traces) in the presence of 100 equiv. pyridine and 100 equiv. pyridine-D5. Dots are experimental data points, and lines correspond to fittings of experimental data by eqn (S1) and (S2).†

The impact of solvent on the ligand dissociation was investigated by conducting DRL experiments in acetonitrile. Using a coordinating solvent dramatically affects the pyridine exchange in solution, as was evidenced by the *k*_−1_ values measured by DRL. For instance, the *k*_−1_ value measured at 40 °C for 1 : 1 CoC–pyridine complexes amounts to *k*_MS-CHCl_3__ = 1.46 × 10^−2^ s^−1^ in CHCl_3_ and *k*_MS-CH_3_CN_ = 2.83 × 10^−4^ s^−1^ in CH_3_CN (Fig. 5 and S10†).

The activation parameters determined for pyridine exchange in acetonitrile also differ from the ones obtained in chloroform (Fig. S11† and Table 1 – entry 2). Most notably, the activation enthalpies and entropies decreased significantly. This striking effect suggests a different ligand exchange mechanism involving a tighter transition state.^41–43^

The greater dielectric constant of acetonitrile likely results in changes of ion pairing in solution, which may affect the equilibria between cage complexes and pyridine ligands in solution. The equilibria will also be affected by the competitive coordination of acetonitrile and pyridine to the metal centre. Any labile axial ligand at the outside of the cavity may significantly affect the rate constant of the pyridine dissociation at the inside of the cavity. A weaker electron-donating acetonitrile ligand can decrease the observed exchange rate for [CoC(CH_3_CN)_OUT_(Py)_IN_]^+^ complexes.

The binding of acetonitrile to the metal centre can be evidenced by the detection of [CoC(CH_3_CN)]^+^ ions by ESI-MS, with negligible intensity compared to the [CoC(Py)]^+^ ions (Fig. S12†). However, the dissociation of acetonitrile from the metal centre is very fast, as evidenced by a DRL experiment with acetonitrile-D3. The equilibrium between CoC–Cl, acetonitrile, and acetonitrile-D3 is established almost instantly after adding CD_3_CN (Fig. S13†). Acetonitrile thus dissociates much faster from the cobalt centre than pyridine. Even though the dissociation of acetonitrile from the CoC complex is very fast, the competitive binding of acetonitrile and pyridine in solution transforms the rate-limiting step of inner pyridine dissociation into a tighter transition step. This step may involve the displacement of an outside-bound acetonitrile ligand by a pyridine molecule to assist the dissociation of the inner pyridine ligand. This illustration highlights the effectiveness of DRL as a tool for identifying subtle changes in ligand exchange mechanisms in solution.

#### Effect of the secondary coordination sphere

**Fig. 6:**
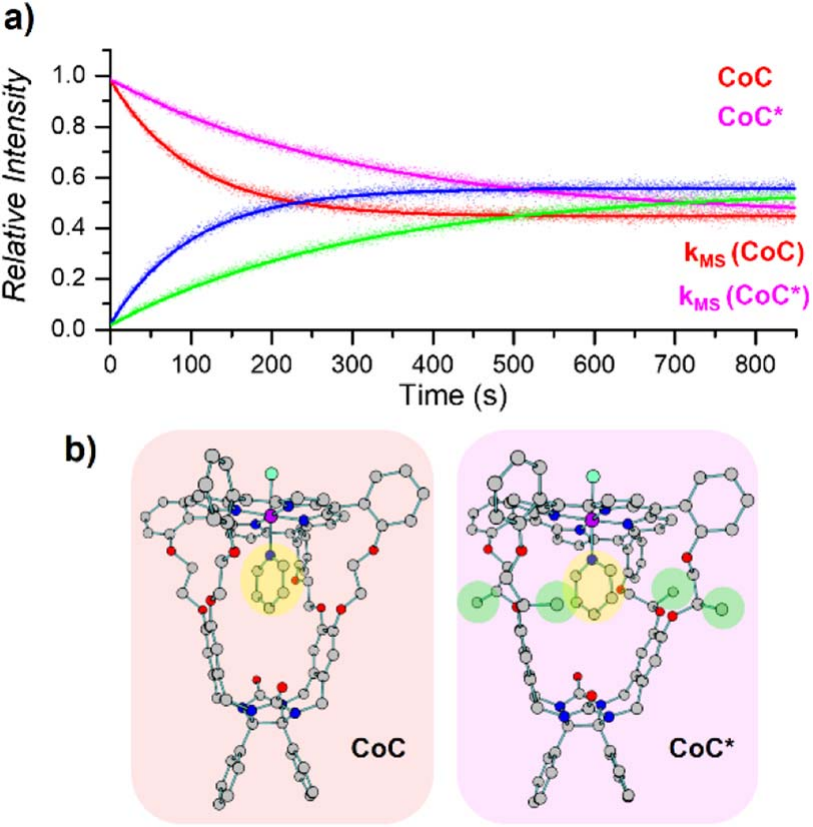
(a) Relative time evolution of [CoC(Py)]^+^ (red), [CoC(Py^L^)]^+^ (blue), [CoC*(Py)]^+^ (magenta), and [CoC*(Py^L^)]^+^ (green) in a single DRL experiment carried out at 40 °C in CHCl_3_ with 200 equiv. pyridine and 200 equiv. pyridine-D5. Dots are experimental data points, and lines correspond to fittings of experimental data by eqn (S1) and (S2).† (b) DFT-calculated structures of ^1^[CoC(Cl_OUT_)(Py_IN_)] and ^1^[CoC*(Cl_OUT_)(Py_IN_)] optimized with B3LYP-D3/def2svp.

We also used the DRL experiments to investigate the impact of the secondary metal coordination sphere, *i.e.* the cage cavity, on the pyridine ligand exchange in solution. DRL experiments were performed in CHCl_3_ with the sterically encumbered cage CoC*–Cl that bears additional methyl groups on the sidewalls of the binding pocket (Fig. 6). The secondary coordination sphere significantly impacts the ligand dissociation in solution, as evidenced by the *k*_−1_ rates. Regardless of the temperature, the dissociation rate of pyridine follows the order CoC–Cl > CoC*–Cl (1.5 × 10^−2^ > 3.7 × 10^−3^ s^−1^ at 40 °C in CHCl_3_). The same trend can be obtained from the Δ*G*^‡^ values for pyridine dissociation from each metal host, as shown in Table 1 – entries 1 & 3 (Fig. S14 and 15†). These values suggest that the presence of methyl groups on the sidewalls of the cage (CoC*–Cl) hinders the dissociation of pyridine ligands from the cavity, resulting in much slower dissociation.

Unlike solution-phase approaches that provide an averaged response for a mixture of compounds, ESI-MS allows the individual monitoring of the many species eventually present based on their *m*/*z* ratios, thereby opening the way for competitive ligand binding experiments. As a proof of concept, the two porphyrin complexes CoC–Cl and CoC*–Cl, were mixed in a single sample with 200 equiv. of pyridine. Upon addition of 200 equiv. pyridine-D5, all metal hosts exchanged their pyridine ligands at different rates, yielding individual *k*_−1_ dissociation rate constants for each host in a single experiment (Fig. 6).

In principle, similar competition experiments could be designed to monitor the equilibrium of metal hosts with multiple ligands in solution, although this was not undertaken in the present work.

#### Nature of the counterion

In the solution phase, all the porphyrin compounds investigated in the previous paragraphs have a chloride counterion. When in excess, pyridine displaces the coordinated Cl^−^ ion, as evidenced by the detection of the [CoC(Py)_2_]^+^ ions (Fig. 2). The complexes with the coordinated counterion are neutral; therefore, they are invisible to our MS technique. Consequently, the role they may play on the dissociation of pyridine ligands inside of the cage cavity must be studied indirectly.

To this end, we carried out experiments with the CoC complex having a non-coordinating hexafluorophosphate counterion (CoC–PF_6_) in the non-coordinating solvent CHCl_3_. The DRL experiments with CoC–PF_6_ reveal about a 3 times lower rate of the pyridine exchange inside the cavity than for CoC–Cl (Fig. S16†). However, the activation parameters for pyridine dissociation do not significantly differ (Table 1 – entry 4 and Fig. S17†), indicating that the exchange mechanism remains the same. Nevertheless, the results suggest that the presence of the coordinating chloride anions in the solution leads to a mixture of cage complexes coordinated with chlorido or pyridine ligands. The coordination of chlorido ligands on the outside of the cage accelerates the dissociation rate of the inner pyridine, as compared to bis-pyridine complexes. In other words, the coordination of more electron-donating *trans*-axial ligands at the outside of the cage accelerates the dissociation of the inner pyridine ligand (*i.e.*, Cl^−^ > pyridine > acetonitrile).

We verified this hypothesis by performing DRL experiments with CoC–PF_6_ in a chloroform solution to which tetrabutylammonium chloride (*t*Bu_4_NCl) was added (Fig. S18†). Mixing CoC–PF_6_ with 5 equiv. *t*Bu_4_NCl accelerates the rate of pyridine dissociation to a similar rate (*k*_−1_) as found for CoC–Cl (Fig. S19†). This experiment confirms the effect of the chlorido ligand on the pyridine exchange kinetics inside the cavity of CoC. Hence, while mass spectrometry cannot provide direct information on the neutral species, the DRL experiments can shed light on the effects of coordinating counterions on the solution kinetics of the detected species.

#### Nature of the transition metal

**Fig. 7:**
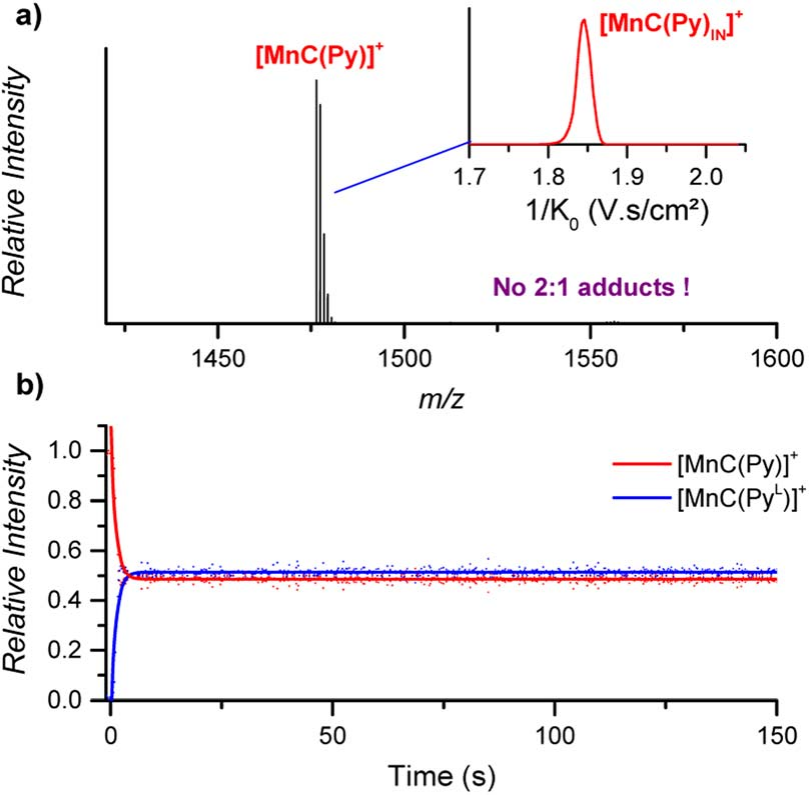
(a) ESI-MS detection of [MnC(Py)]^+^ (*m*/*z* 1476.4) from an acetonitrile solution. Inset: ion mobility separation of [MnC(Py)]^+^. (b) Relative time evolution of the intensities of [MnC(Py)]^+^ and [MnC(Py^L^)]^+^ in a DRL experiment carried out at 24 °C in CH_3_CN with 100 equiv. pyridine and 100 equiv. pyridine-D5. Dots are experimental data points, and lines correspond to fittings of experimental data by eqn (S1) and (S2).†

The NMR spectroscopic analysis of paramagnetic metal complexes is challenging because of the effect of signal broadening. The DRL approach can thus be an elegant alternative to NMR for such systems. We conducted DRL experiments to probe the solution equilibria between the paramagnetic Mn(iii) porphyrin cage complex MnC–Cl and pyridine ligands. As shown in Fig. 7, (i) the equilibrium between MnC–Cl, pyridine, and pyridine-D5 was almost immediately established, and (ii) 1 : 2 cage-pyridine adducts were not detected by ESI-MS (Fig. 7a).

The fast ligand exchange and the absence of complexes with the pyridine ligand at the outside of the porphyrin cage (as was demonstrated by ion mobility, see Fig. 7a – inset) suggest that the binding of pyridine ligands to the Mn(iii) centre is much more labile than that that of the Co(iii) centre in the previously discussed cobalt porphyrin complexes. DFT calculations predict that the Mn–N bonding distance in [MnC(Py_IN_–Cl_OUT_)] is 2.58 Å, which is considerably longer than the Co–N distance in Co–N(Py) for [CoC(Py_IN_–Cl_OUT_)] (2.09 Å, see Fig. S20†). Accordingly, a smaller pyridine binding constant in the Mn(iii) complex is expected compared to that in the analogous Co(iii) complex. Moreover, the predicted free energy required to dissociate the inner pyridine ligand from [MnC(Py_IN_–Cl_OUT_)] complexes amounts to only 12.6 kcal mol^−1^ (Fig. S21†). These results also agree with NMR binding studies performed by Olsson *et al.* on Co(iii) and Mn(iii) metalloporphyrins.^13^

While DRL highlights the difference in pyridine binding in CoC–Cl and MnC–Cl, the fast dissociation kinetics of the MnC–pyridine complexes does not enable an accurate measurement of *k*_MS_. Indeed, the time required to add the isotopically labelled pyridine and to infuse the solution to the ESI source precludes the quantitative application of DRL using the current experimental setup to reactions with *k*_MS_ > 0.5 s^−1^. Therefore, activation parameters for the dissociation of pyridine from MnC–Cl cannot be obtained. Interestingly, the fast dissociation of pyridine is not significantly slowed down by using a non-coordinating counterion since fast dissociation kinetics were also observed for MnC–PF_6_ (Fig. S22†). Moreover, lowering the temperature to 0 °C did not slow down the reaction enough to enable an accurate measurement of *k*_MS_.

### On the dissociation of 1 : 2 cage-pyridine complexes

**Fig. 8:**
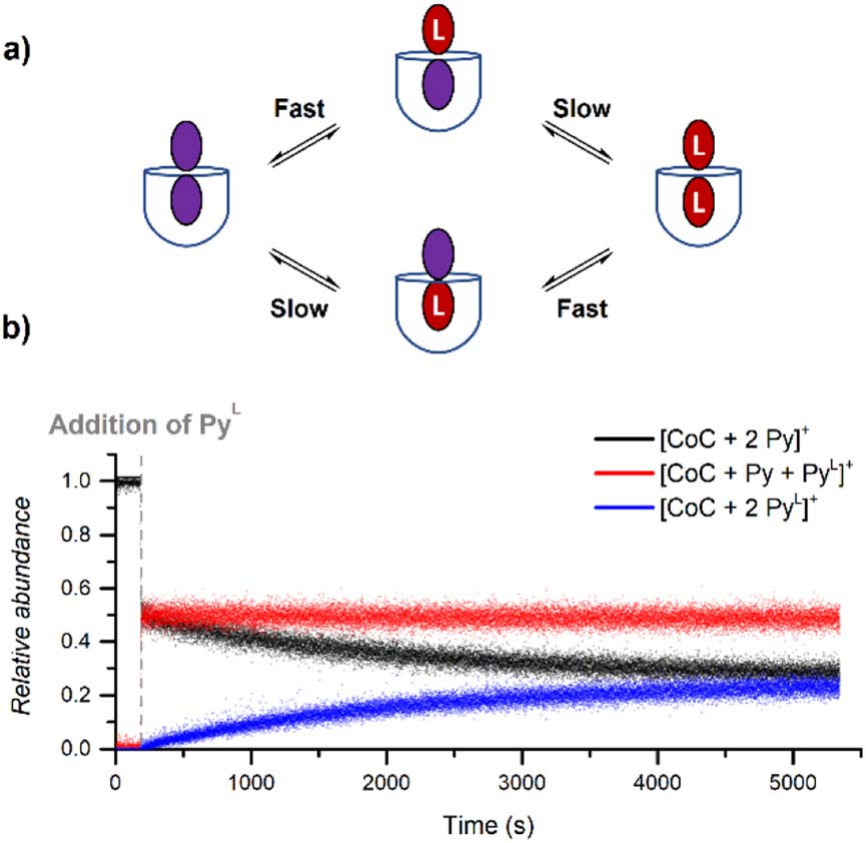
(a) Schematic representation of the two ligand exchange processes taking place for [CoC(Py)_2_]^+^. (b) Relative time evolution of the intensities of [CoC(Py)_2_]^+^, [CoC(Py)(Py^L^)]^+^ and [CoC(Py^L^)_2_]^+^ in a DRL experiment carried out at 24 °C in CHCl_3_ with 100 equiv. pyridine and 100 equiv. pyridine-D5.

During DRL experiments, [CoC(Py)_2_]^+^ (*m*/*z* 1559.5), [CoC(Py)(Py^L^)] (*m*/*z* 1564.5), [CoC(Py^L^)_2_]^+^ (*m*/*z* 1569.5) are detected next to the 1 : 1 complexes. Monitoring the relative abundance of these ions over time in a DRL experiment with 200 equiv. pyridine(-D5) (Fig. 8) reveals two ligand exchange processes with very different rates. The first one can be observed almost immediately after the addition of the isotopically labelled pyridine, as evidenced by the sharp increase in abundance of [CoC(Py)(Py^L^)]^+^. The first fast event can thus be attributed to a fast pyridine exchange in solution, leading to the formation of [CoC(Py)(Py^L^)]^+^. The second exchange corresponds to the slow appearance of [CoC(Py^L^)_2_]^+^.

The rate constant for the second CoC–pyridine exchange can be obtained by renormalizing together the relative abundance of [CoC(Py)_2_]^+^ (*m*/*z* 1559.5) and [CoC(Py^L^)_2_]^+^ (*m*/*z* 1569.5) and fitting them with eqn (S1) and (S2).† The observed rate constants *k*_−1_ obtained for the second event are almost identical to rate constants measured for the dissociation of 1 : 1 CoC–pyridine (5.4 × 10^−4^ s^−1^ for 1 : 2 complexes *vs.* 5.1 × 10^−4^ s^−1^ for 1 : 1 complexes at 24 °C). Since we only monitor the dissociation of the pyridine ligand bound inside of the cavity for 1 : 1 complexes, the second dissociation observed in Fig. 8 reports on removing the pyridine ligand bound inside of the bis-pyridine cage complexes.

The first fast exchange corresponding to the sharp increase in abundance of [CoC(Py)(Py^L^)]^+^ can be related to the rapid dissociation of the pyridine ligand coordinated outside the cavity. However, the fast dissociation kinetics of the outside-bound pyridine ligands is out of the mass spectrometry resolution range. As discussed above, the current setup allows to monitor reactions with *k*_MS_ > 0.5 s^−1^. While the rate of this event is too fast to be accurately measured by DRL, this result highlights that the two binding sites of the porphyrin cage are not equivalent concerning ligand dissociation. This result qualitatively agrees with predicted free energy for the dissociation of the outer pyridine ligand from bispyridine complexes 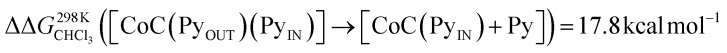 (Fig. S23†), which is lower than the free energy required to remove the inner pyridine ligand (22.0 kcal mol^−1^ – Fig. S9†). As a comparison, DRL experiments performed with the CoTP–Cl complex, which does not bear the glycoluril pocket, also show fast dissociation for 1 : 1 CoTP : pyridine complexes (Fig. S24†).

## Conclusions

We have introduced the delayed reactant labelling method to quantitatively monitor the solution-phase equilibria between metal complexes and labile ligands. As a proof of concept, the equilibria between metal–porphyrin cage complexes and pyridine were investigated. Upon introduction of isotopically labelled pyridine-D5 in a mixture of a metal complex and non-labelled pyridine, the continuous monitoring of the system by ESI-MS enables the tracking of the appearance rate of metal-bound pyridine-D5 in solution. Moreover, hyphenating MS detection with ion mobility separation enables targeting specific isomers, such as pyridine ligands bound inside the porphyrin cage cavity. The method is suitable for processes with rate constants smaller than 0.5 s^−1^. In future studies, the monitoring of fast processes could benefit from approaches involving flow chemistry,^23^ such as the double syringe method.^44^ This could enable the measurement of the relative intensities of [CoC(Py)]^+^ and [CoC(Py)^L^]^+^ for short reaction times.

With the help of a kinetic model, we find that rate constants measured with a large excess of pyridine correspond to ligand dissociation rates in solution. The measurement of dissociation rates at different temperatures leads to activation parameters for ligand dissociation in solution and provides mechanistic insights into the ligand exchange processes. The effect of solvent, secondary coordination sphere around the metal centre, presence of a coordinating counterion, and nature of the metal centre were investigated. We found that the use of acetonitrile as a solvent instead of chloroform modified the mechanism of pyridine exchange, through a competitive coordination of pyridine and solvent molecules to the metal centre. Moreover, the coordination sphere around the pyridine ligand and nature of the *trans*-axial ligand were found to play a significant role. The more electron-donating *trans*-axial ligands accelerate the dissociation of the inner pyridine ligand. We also highlight that the presence of chlorido ligands results in a complex mixture of cage complexes coordinated to chlorido and pyridine ligands, and that the measured *k*_MS_ is an average value for all cage complexes exchanging pyridine ligands inside the cavity.

We note that ESI-MS approaches were previously reported to investigate ligand exchange and self-exchange processes.^45–51^ However, the information obtained was mainly qualitative, limited to comparisons between slow and fast processes or determining which mechanisms contribute or do not contribute to the exchange. The present work is the first example of the derivation of dissociation rate constants and thermodynamic parameters by ESI-MS, for ligand self-exchange in solution. Our method opens a way for the quantitative monitoring of metal–ligand equilibria in solution, regardless of the nature and oxidation state of the metal centre. Even if the metal–ligand complexes are neutral in solution, DRL can provide quantitative and targeted information on the kinetic processes they are involved in, provided the complexes can ionize by losing a charged ligand. As for organic reaction mechanisms,^52^ it is envisioned that future studies could take advantage of machine learning to decipher and classify the speciation and ligand exchange mechanisms in solution based on datasets generated by DRL experiments.

## Experimental section

Ion mobility-mass spectrometry experiments were performed with a timsToF instrument (Bruker, Germany) equipped with an ESI source. Ions were electrosprayed in positive mode with a source voltage of +5.5 kV, with a Nebulizer of 0.2 Bar, a drying gas flow of 2 L min^−1^, and the End Plate Offset set to 500 V. Typical ion transfer voltages were quadrupole ion energy = 3 eV and collision energy = 5 eV. The mass range scanned by the ToF analyser was *m*/*z* 1200–2000. TIMS experiments were performed in N_2_ using the imeX Detect mode, by scanning ion mobility from 1.3 V s cm^−2^ to 2.08 V s cm^−2^. The accumulation time was varied from 0 to 30 ms depending on the pyridine concentration, in order to maintain the maximum ion signal lower than 2 × 10^4^ counts and thereby minimize ion activation in the ion mobility region. Additional details regarding the instrument settings, preparation of reaction mixtures and data analysis can be found in the ESI.†

DFT calculations were carried out at the B3LYP-D3 (ref. 53–55)/def2svp^56^ level with the Gaussian 16 package.^57^ The SMD model was used to account for implicit chloroform solvation.^58^ All reported structures correspond to potential energy surface minima as confirmed by analyses of the corresponding Hessian matrixes. Reported energies include zero-point vibrational energy corrections and thermal corrections calculated at the same level of theory. Reported Δ*G* values also include a correction of (1.9 Δ*n*) kcal mol^−1^ to account for the change in the number of moles (*n*) for reactions involving the dissociation of a ligand. The molecular coordinates of all optimized geometries are included in the ESI.†

The synthesis procedures and characterization of the metal porphyrin compounds can be found in the ESI.†

## Data availability

All the processed DRL data are available as ESI.† Cartesian coordinates of the computed structures, the script used for the kinetic model, and the NMR spectra of the characterized compounds are available as ESI.† Crystallographic data have been deposited at the Cambridge Crystallographic Data Centre (CCDC: 2255454 and 2255455).

## Author contributions

QD and JR designed the research and methodology. JAAWE synthesized, characterized the metal porphyrin complexes, and grew the crystals of the complexes. PT performed the crystallographic analysis. QD carried out the ESI-MS experiments and DFT calculations. QD and JR wrote the initial draft of the manuscript. All authors discussed the results and commented on the manuscript. JR supervised the study and acquired funding.

## Conflicts of interest

There are no conflicts of interest to declare.

## Supplementary Material

SC-014-D3SC03342B-s001

SC-014-D3SC03342B-s002

SC-014-D3SC03342B-s003

SC-014-D3SC03342B-s004
