# Supplementary material for: Kinetics of ligand exchange in solution: a quantitative mass spectrometry approach
Source: Chem Sci. 2023 Aug 24;14(36):9759–69. doi: 10.1039/d3sc03342b (PMC10510763; doi:10.1039/d3sc03342b)
Supplement: SC-014-D3SC03342B-s003 [file SC-014-D3SC03342B-s003.pdf]

Electronic Supplementary Material (ESI) for Chemical Science.
This journal is © The Royal Society of Chemistry 2023

Kinetics of Ligand Exchange in Solution: A Quantitative Mass Spectrometry Approach

Quentin Duez*, Paul Tinnemans, Johannes A. A. W. Elemans and Jana Roithová*

Radboud University, Institute for Molecules and Materials, Heyendaalseweg 135, 6525 AJ, Nijmegen, The Netherlands.

*Email: j.roithova@science.ru.nl and quentin.duez@ru.nl

Supplementary Information

This file contains:

Supplementary Figures – Page 2

Experimental section – Page 17

Synthesis procedures – Page 21

Kinetic model – Page 35

References – Page 42

Supplementary Figures

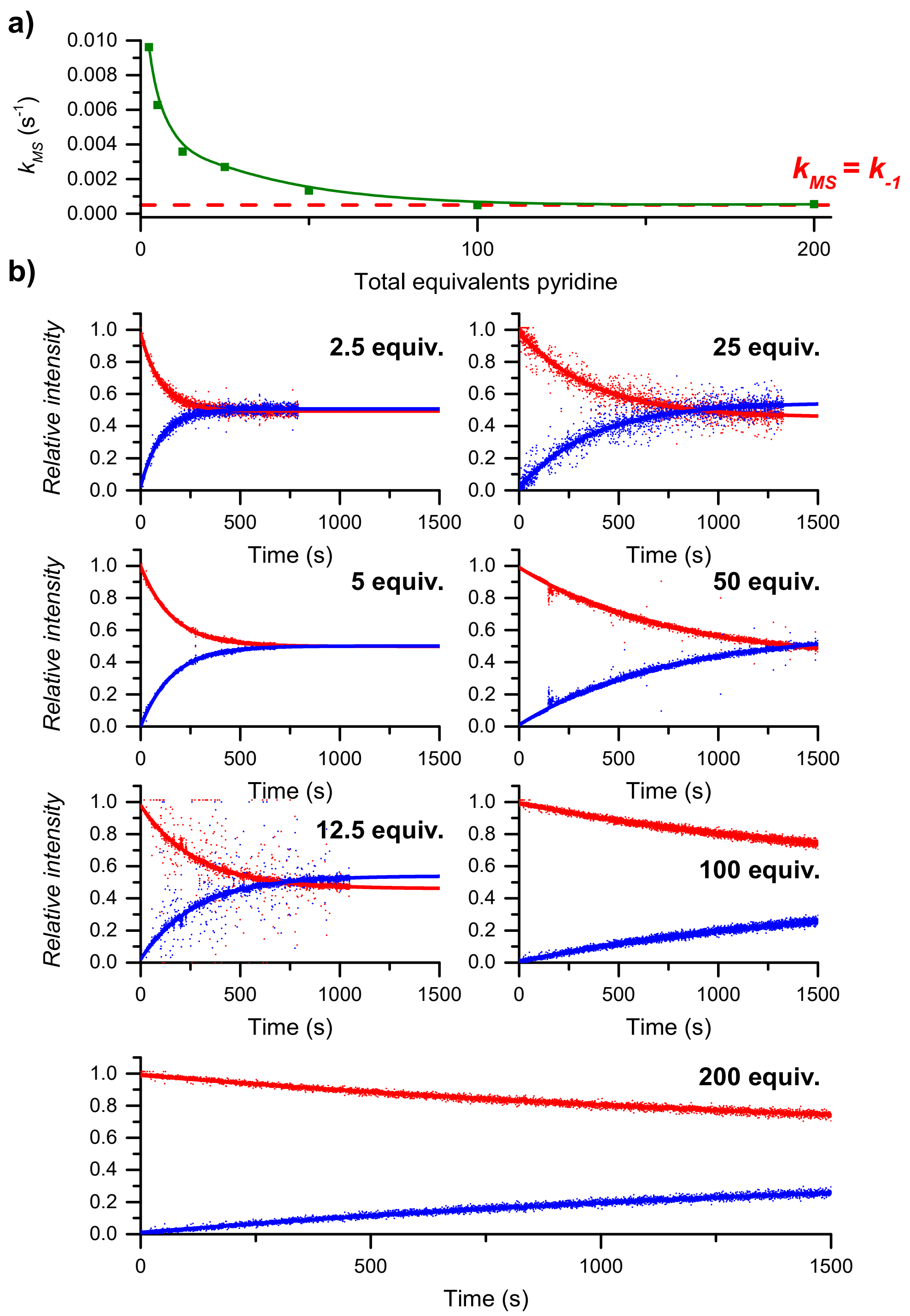

Figure S1. a) Evolution of k_{MS} as measured by DRL experiments in $CHCl_3$ at 24°C with increasing equivalents of (pyridine + pyridine-D5). For each experiment, a 1:1 ratio of pyridine and pyridine-D5 was used. The green line is a guide for the eyes. **b)** Relative time evolution of $[CoC(Pyridine)]^+$ (red dots) and $[CoC(Pyridine\text{-}D5)]^+$ (blue dots) in DRL experiments recorded at 24°C with CoC-Cl after addition of various equiv. of pyridine(-D5) in $CHCl_3$. Dots are experimental data points and lines correspond to fittings of experimental data by Equation S1 (red lines) and S2 (blue lines).

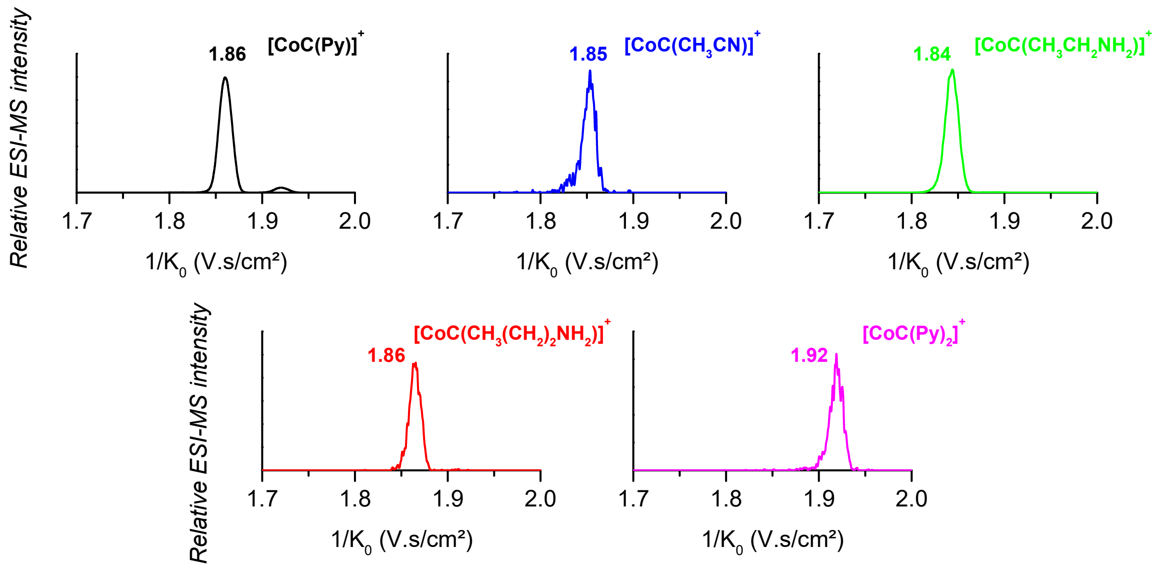

Figure S2. Ion mobility spectra of the CoC complex with various guests: $[CoC(Py)]^+$, $[CoC(CH_3CN)]^+$, $[CoC(CH_3CH_2NH_2)]^+$, $[CoC(CH_3CH_2CH_2NH_2)]^+$ and $[CoC(Py)_2]^+$. The first four guests are proposed to bind inside the cage cavity, since the binding of different guests results in complexes with similar collisional cross section (CCS) (The size difference between $1/K_0$ = 1.84 and 1.86 V.s/cm² corresponds to ΔCCS_{N2} = 4.1 Å²). The second pyridine in $[CoC(Py)_2]^+$ binds outside the cavity and results in a complex with larger size.

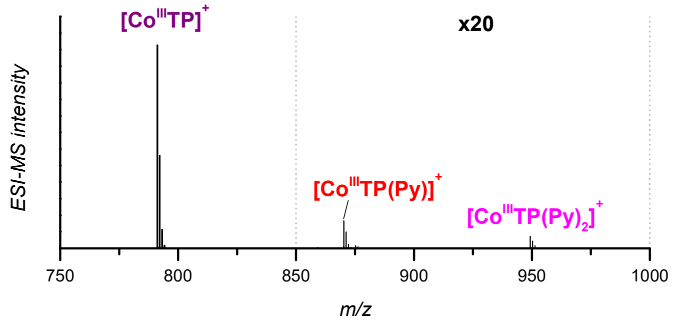

Figure S3. ESI-MS detection of $[Co^{III}TP]^+$ (*m/z* 791.2), $[Co^{III}TP(Py)]^+$ (*m/z* 870.2), $[Co^{III}TP(Py)_2]^+$ (*m/z* 949.3). The area between *m/z* 850-1000 is magnified 20x.

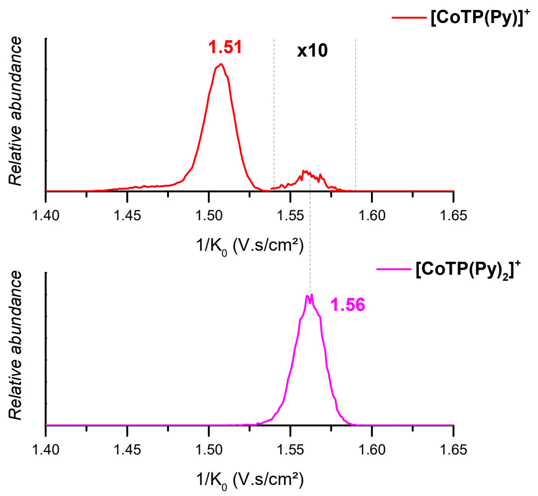

Figure S4. Ion mobility separation of $[Co^{III}TP(Py)]^+$ (*m/z* 870.2- up) and $[Co^{III}TP(Py)_2]^+$ (*m/z* 949.3 - down). In the top graph, the area between $1/K_0$ 1.54 and 1.59 V.s/cm² is magnified 10x.

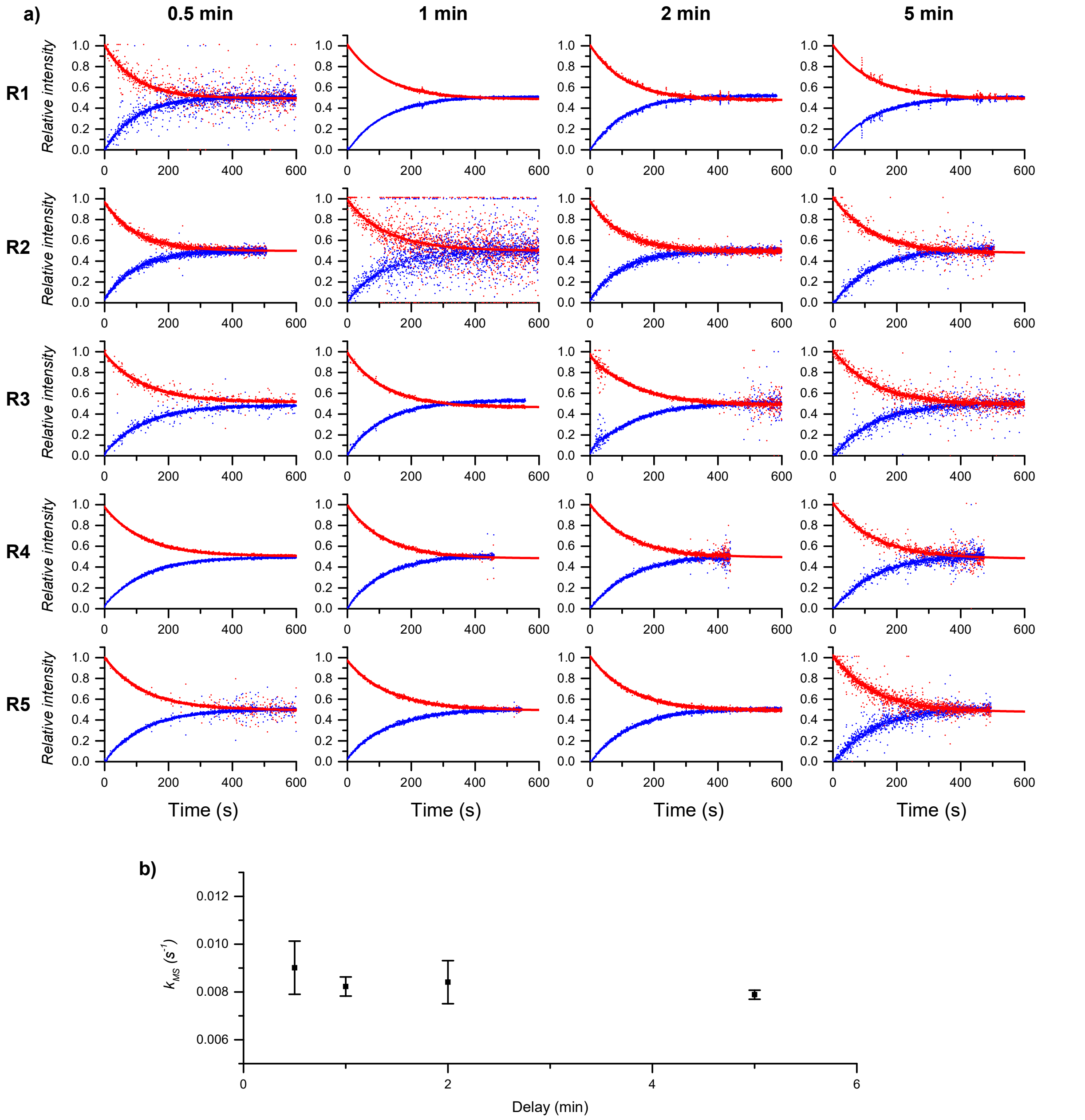

Figure S5. a) Relative time evolution of $[CoC(Pyridine)]^+$ (red dots) and $[CoC(Pyridine\text{-}D5)]^+$ (blue dots) in DRL experiments recorded at 24°C with CoC-Cl after addition of 1.25 equiv. of pyridine and 1.25 equiv. pyridine-D5 in $CHCl_3$, with different delays. Dots are experimental data points and red lines correspond to fittings of experimental data by Equation S1. Each experiment was repeated five times, and each replica is denoted by R1, R2, R3, R4 and R5. **b)** Average values of k_{MS} measured for each time delay between addition of pyridine and pyridine-D5. Error bars correspond to the standard deviation.

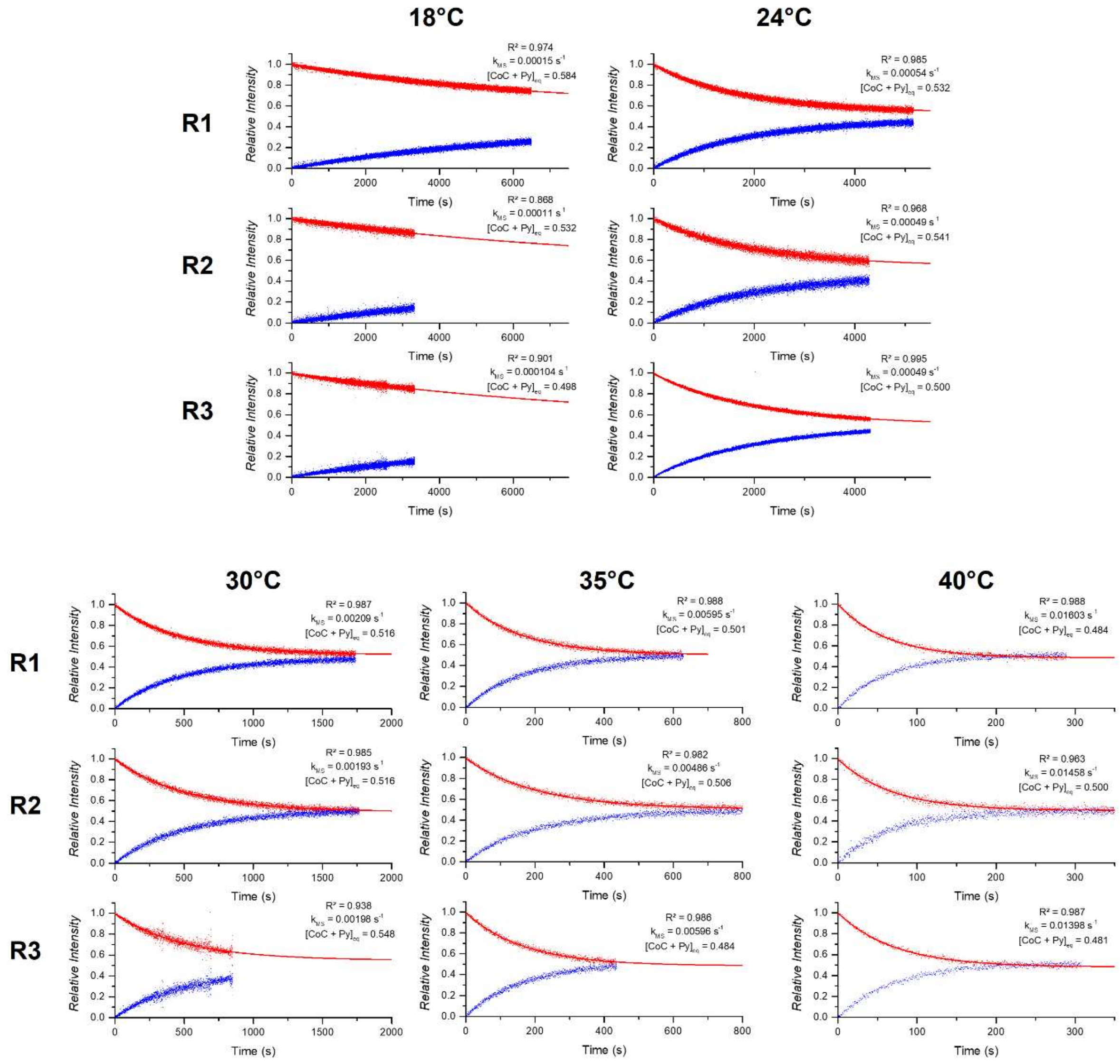

Figure S6. Relative time evolution of $[CoC(Pyridine)]^+$ (red dots) and $[CoC(Pyridine\text{-}D5)]^+$ (blue dots) in DRL experiments recorded at 18°C, 24°C, 30°C, 35°C and 40°C for CoC-Cl after addition of 100 equiv. pyridine and 100 equiv. pyridine-D5 in $CHCl_3$. Dots are experimental data points and red lines correspond to fittings of experimental data by Equation S1. Each experiment was repeated three times, and each replica is denoted by R1, R2, R3. The values of k_{MS} and $[CoC + Py]_{eq}$ obtained from fittings are indicated for each experiment.

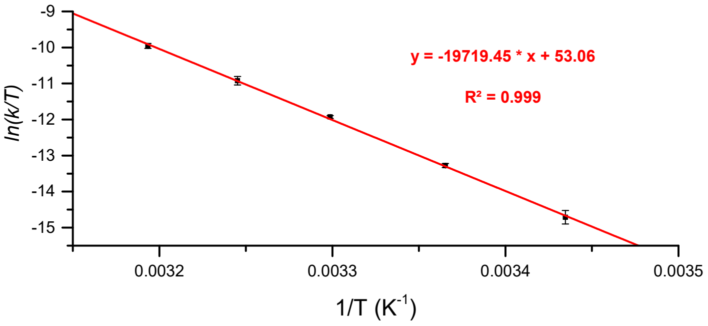

Figure S7. Eyring plot from variable temperature DRL experiments for CoC-Cl after addition of 100 equiv. pyridine and 100 equiv. pyridine-D5 in $CHCl_3$. Dots correspond to the average of ln(k/T) values from triplicate measurements, with error bars corresponding to the standard deviation.

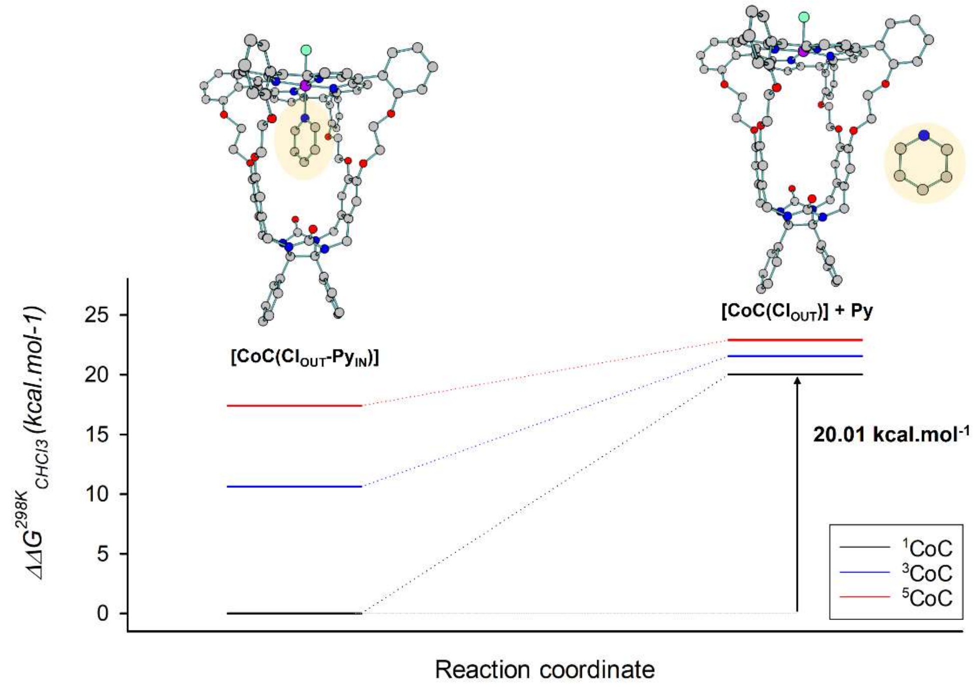

Figure S8. Free energy scheme for the pyridine ligand dissociation mechanism for [Co^{III}C(Cl$_{OUT}$)(Py$_{IN}$)]. Spin states of the Co(III) are written as follow: Singlet ^{1}CoC (black), Triplet ^{3}CoC (blue), Quintet ^{5}CoC (red). DFT structures are only shown for the ^{1}CoC state. H atoms are omitted for clarity.

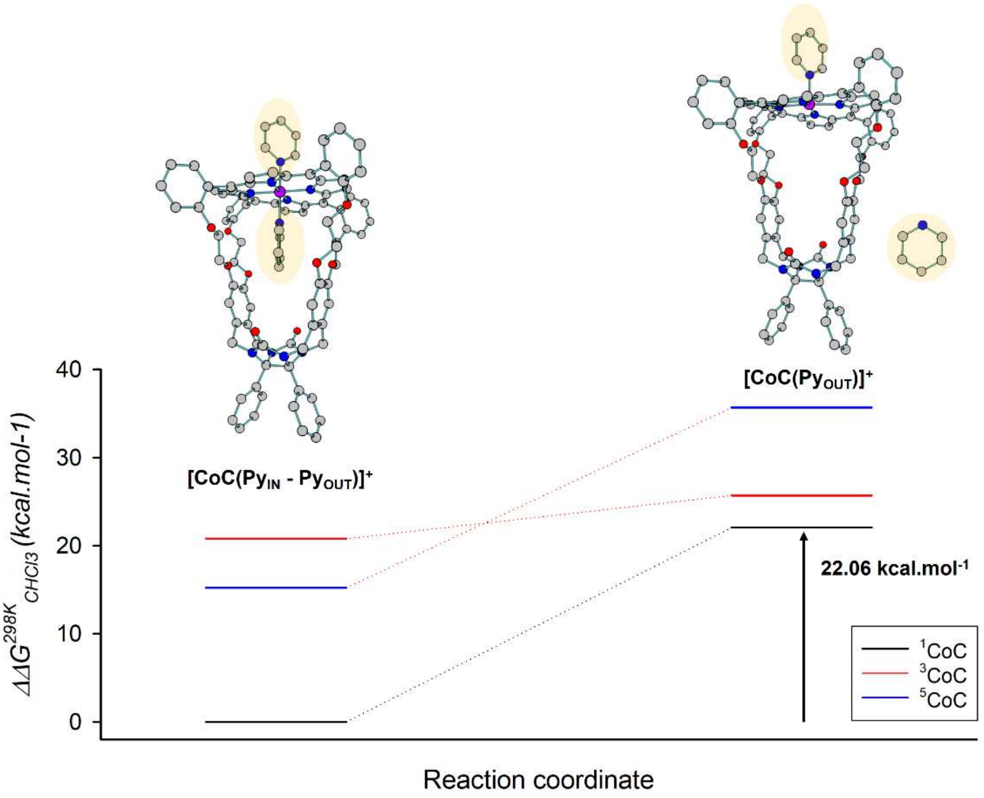

Figure S9. Free energy scheme for the inner pyridine ligand dissociation mechanism for [Co^{III}C(Py$_{OUT}$)(Py$_{IN}$)]. Spin states of the Co(III) are written as follow: Singlet ^{1}CoC (black), Triplet ^{3}CoC (blue), Quintet ^{5}CoC (red). DFT structures are only shown for the ^{1}CoC state. H atoms are omitted for clarity.

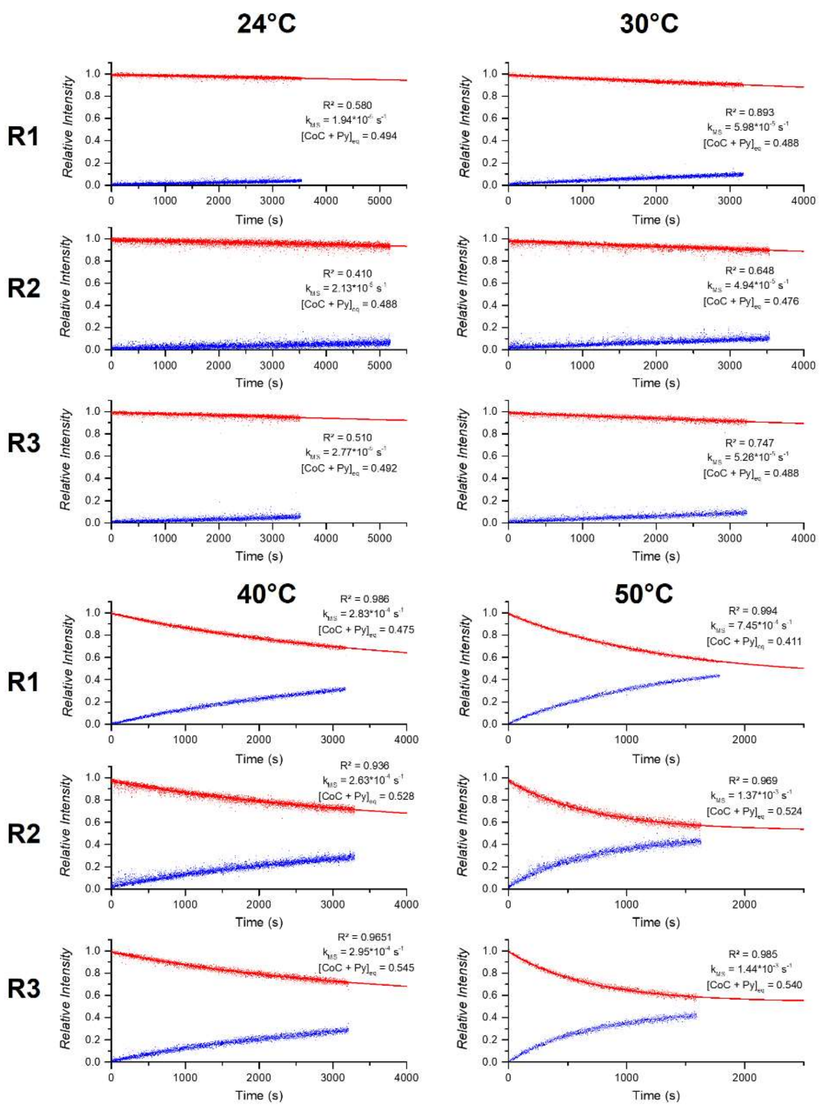

Figure S10. Relative time evolution of $[CoC(Pyridine)]^+$ (red dots) and $[CoC(Pyridine\text{-}D5)]^+$ (blue dots) in DRL experiments recorded at 24°C, 30°C, 40°C and 50°C for CoC-Cl after addition of 100 equiv. pyridine and 100 equiv. pyridine-D5 in acetonitrile. Dots are experimental data points and red lines correspond to fittings of experimental data by Equation S1. Each experiment was repeated three times, and each replica is denoted by R1, R2, R3. The values of k_{MS} and $[CoC + Py]_{eq}$ obtained from fittings are indicated for each experiment.

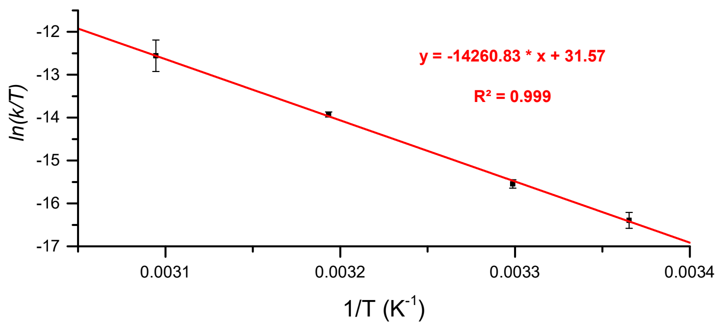

Figure S11. Eyring plot from variable temperature DRL experiments for CoC-Cl after addition of 100 equiv. pyridine and 100 equiv. pyridine-D5 in acetonitrile. Dots correspond to the average of ln(k/T) values from triplicate measurements, with error bars corresponding to the standard deviation.

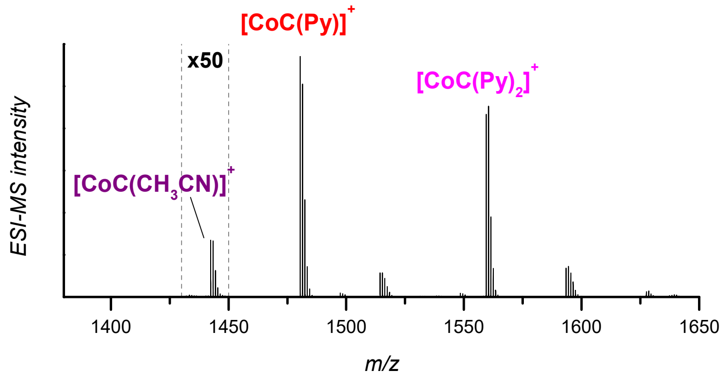

Figure S12. ESI-MS detection of $[CoC(CH_3CN)]^+$ (*m/z* 1442.4), $[CoC(Pyridine)]^+$ (*m/z* 1480.4), $[CoC(Pyridine)_2]^+$ (*m/z* 1559.4) by infusion of a mixture of CoC-Cl with 100 equiv. pyridine in acetonitrile. Additional signals are observed at *m/z* 1515.4 and 1594.4 and can be attributed to $[CoC – H + Py + Cl]^+$ and to $[CoC – H + 2 Py + Cl]^+$.

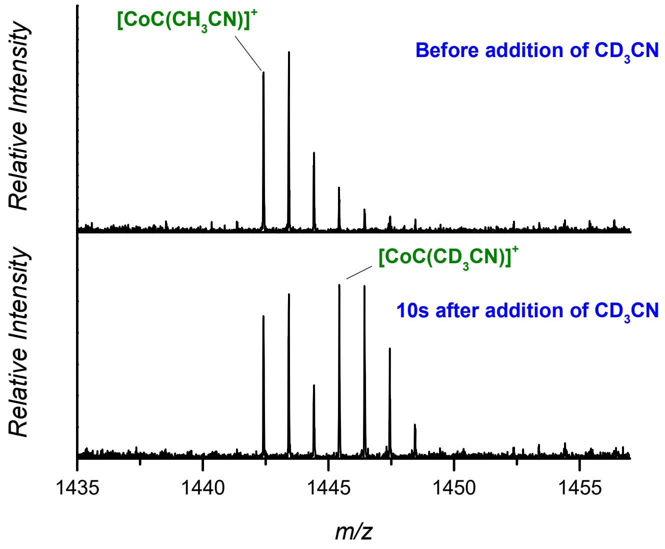

Figure S13. ESI-MS detection of $[CoC(CH_3CN)]^+$ (*m/z* 1442.4) and $[CoC(CD_3CN)]^+$ (*m/z* 1445.4) after addition of 1mL CD_3CN to a stirring solution of CoC-Cl with 100 equiv. pyridine in acetonitrile. The fast appearance of $[CoC(CD_3CN)]^+$ ions indicates a fast dissociation of the acetonitrile ligand from the cobalt centre.

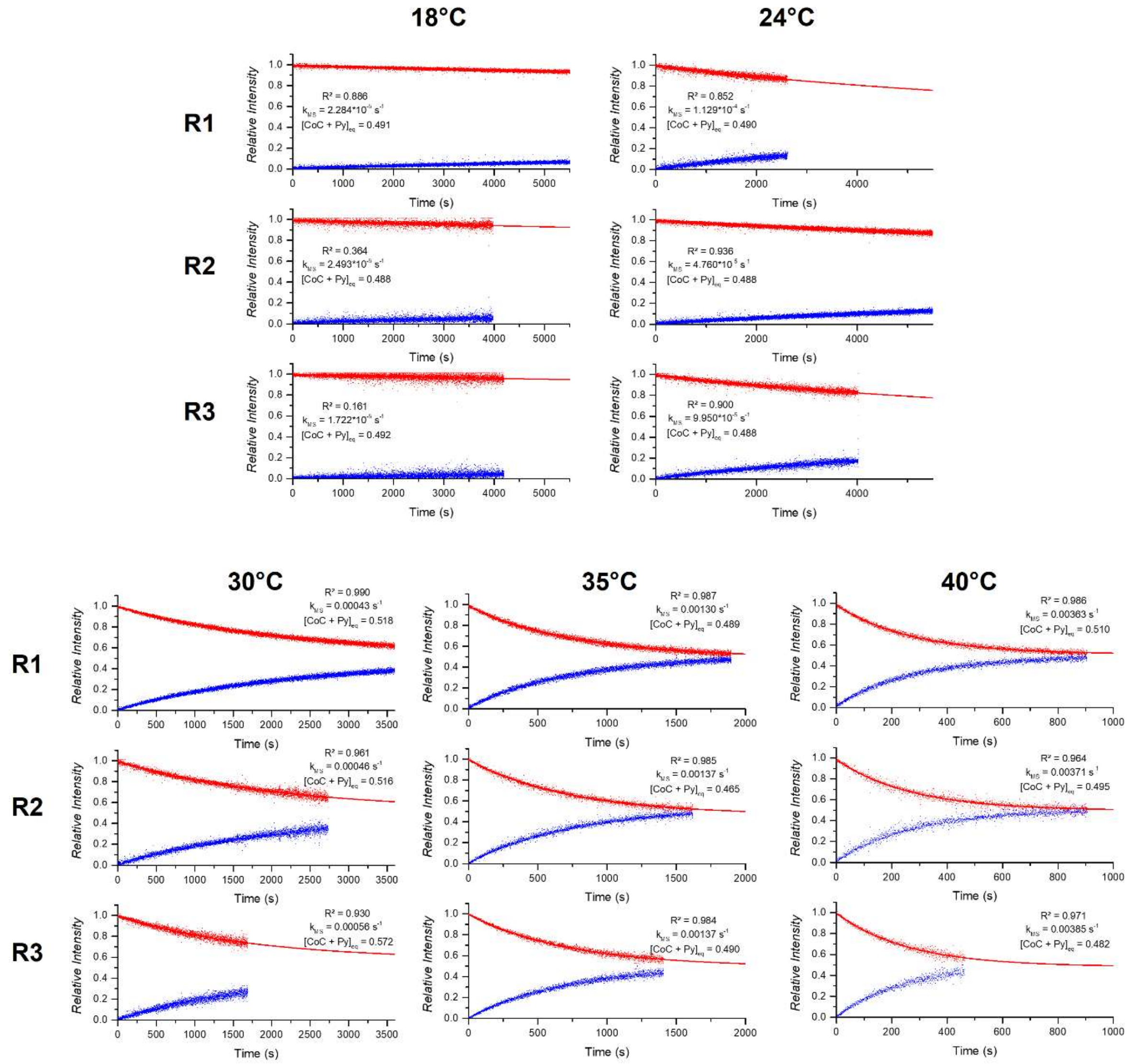

Figure S14. Relative time evolution of [CoC*(Pyridine)]$^+$ (red dots) and [CoC*(Pyridine-D5)]$^+$ (blue dots) in DRL experiments recorded at 18°C, 24°C, 30°C, 35°C and 40°C for CoC*-Cl after addition of 100 equiv. pyridine and 100 equiv. pyridine-D5 in $CHCl_3$. Dots are experimental data points and red lines correspond to fittings of experimental data by Equation S1. Each experiment was repeated three times, and each replica is denoted by R1, R2, R3. The values of k_{MS} and [CoC* + Py]$_{eq}$ obtained from fittings are indicated for each experiment.

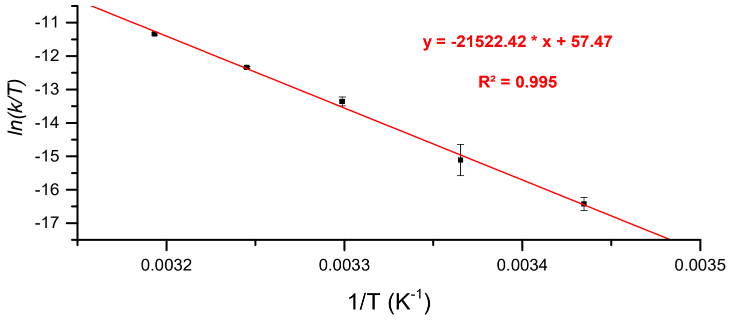

Figure S15. Eyring plot from variable temperature DRL experiments for CoC*-Cl after addition of 100 equiv. pyridine and 100 equiv. pyridine-D5 in $CHCl_3$. Dots correspond to the average of ln(k/T) values from triplicate measurements, with error bars corresponding to the standard deviation.

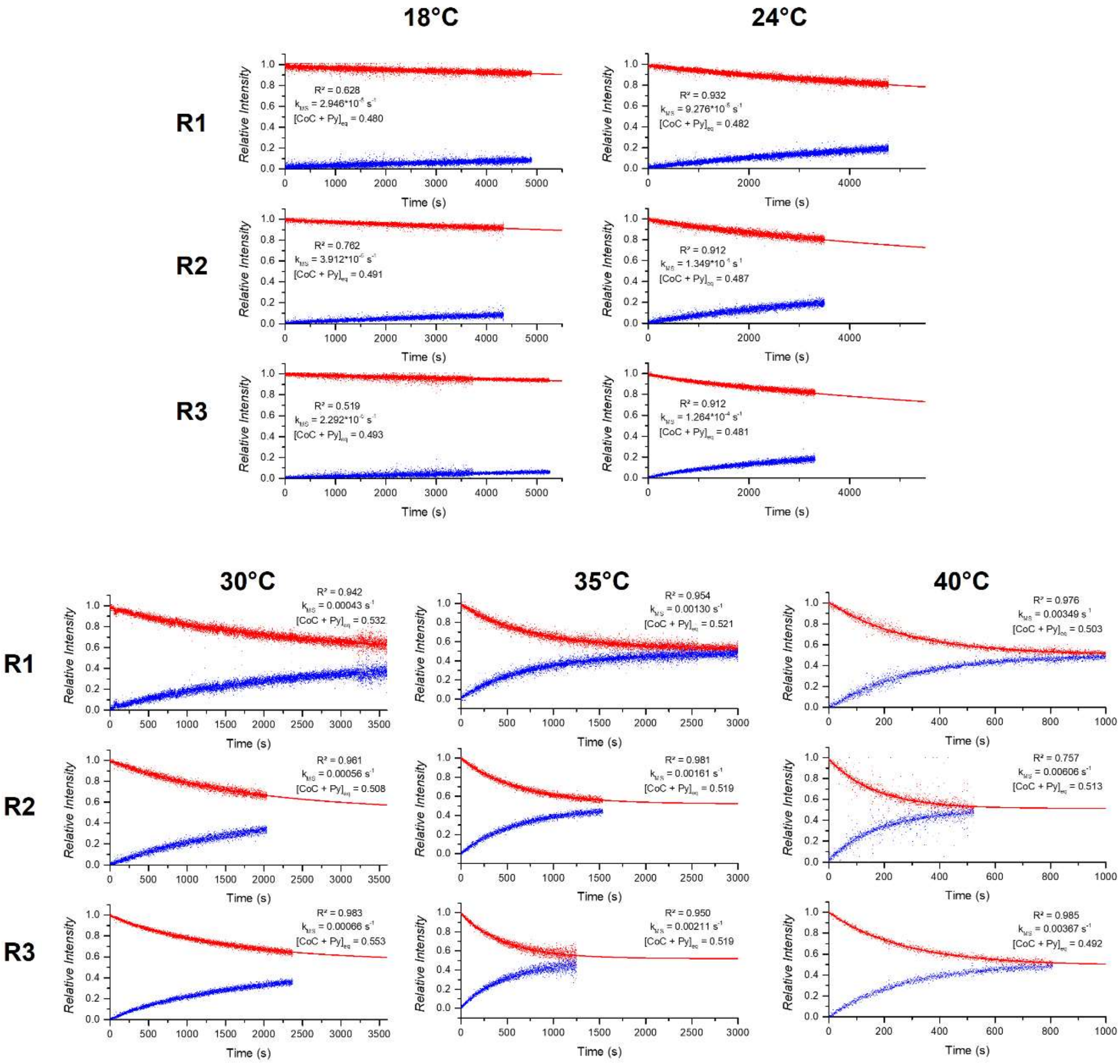

Figure S16. Relative time evolution of $[CoC(Pyridine)]^+$ (red dots) and $[CoC(Pyridine\text{-}D5)]^+$ (blue dots) in DRL experiments recorded at 18°C, 24°C, 30°C, 35°C and 40°C for $CoC\text{-}PF_6$ after addition of 100 equiv. pyridine and 100 equiv. pyridine-D5 in $CHCl_3$. Dots are experimental data points and red lines correspond to fittings of experimental data by Equation S1. Each experiment was repeated three times, and each replica is denoted by R1, R2, R3. The values of k_{MS} and $[CoC + Py]_{eq}$ obtained from fittings are indicated for each experiment.

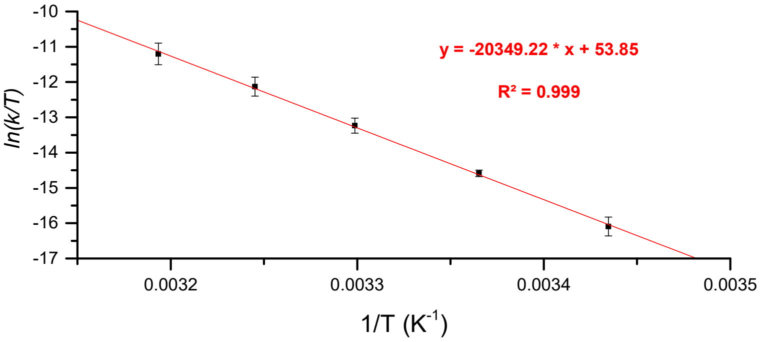

Figure S17. Eyring plot from variable temperature DRL experiments for $CoC\text{-}PF_6$ after addition of 100 equiv. pyridine and 100 equiv. pyridine-D5 in $CHCl_3$. Dots correspond to the average of ln(k/T) values from triplicate measurements, with error bars corresponding to the standard deviation.

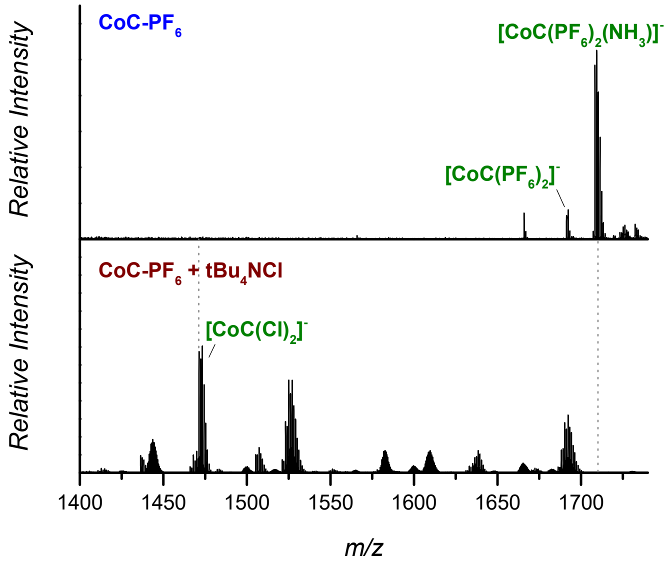

Figure S18. ESI-MS detection of (*top*) $[CoC(PF_6)_2]^-$ (*m/z* 1691.3) by infusion of a 0.5 mM solution of CoC-PF$_6$, and of (*bottom*) $[CoC(Cl)_2]^-$ by infusion of a 0.5 mM solution of CoC-PF$_6$ with 10 equiv. of tBu$_4$NCl.

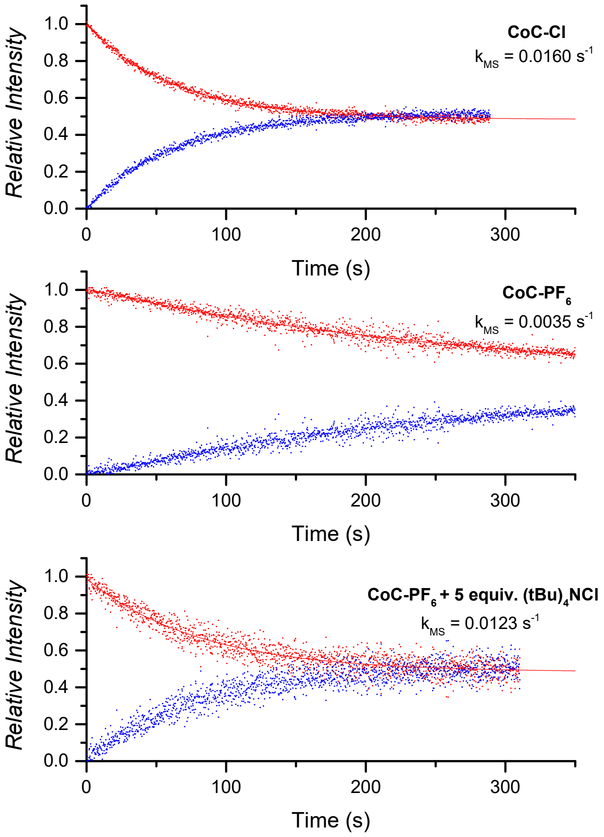

Figure S19. Relative time evolution of $[CoC(Pyridine)]^+$ (red dots) and $[CoC(Pyridine\text{-}D5)]^+$ (blue dots) in DRL experiments recorded with CoC-Cl (up), CoC-PF$_6$ (middle) and CoC-PF$_6$ mixed with 5 equiv. of tetrabutylammonium chloride ((tBu)$_4$NCl), in $CHCl_3$ at 40°C.

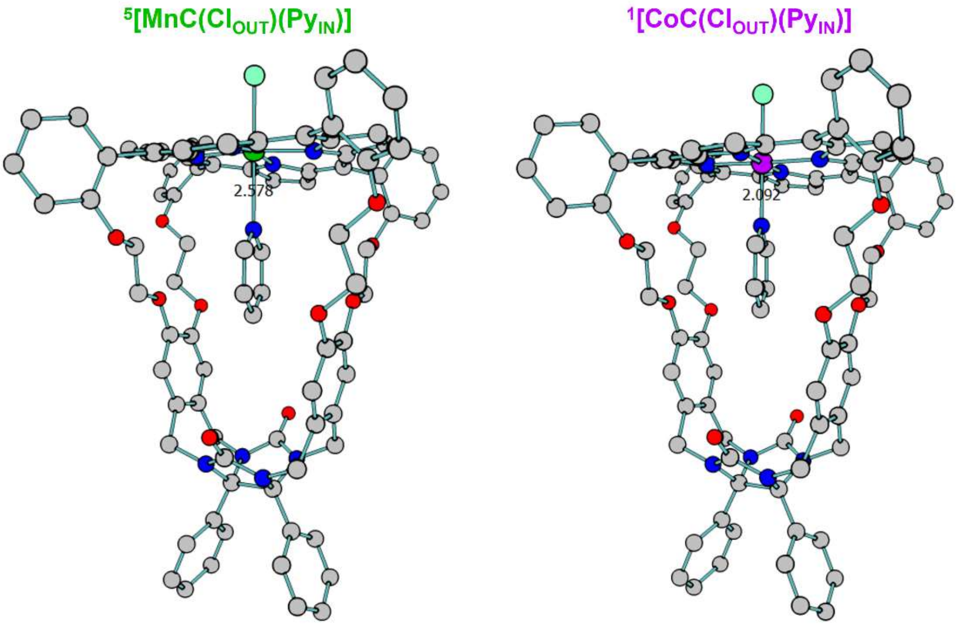

Figure S20. DFT calculated structures of [MnC(Cl$_{OUT}$)(Py$_{IN}$)] and [CoC(Cl$_{OUT}$)(Py$_{IN}$)] in their most stable spin state, *i.e.*, quintet and singlet respectively, optimized with B3LYP-D3/def2svp. The distance between the metal centre and the nitrogen atom of the pyridine is highlighted. Hydrogen atoms are not shown for clarity.

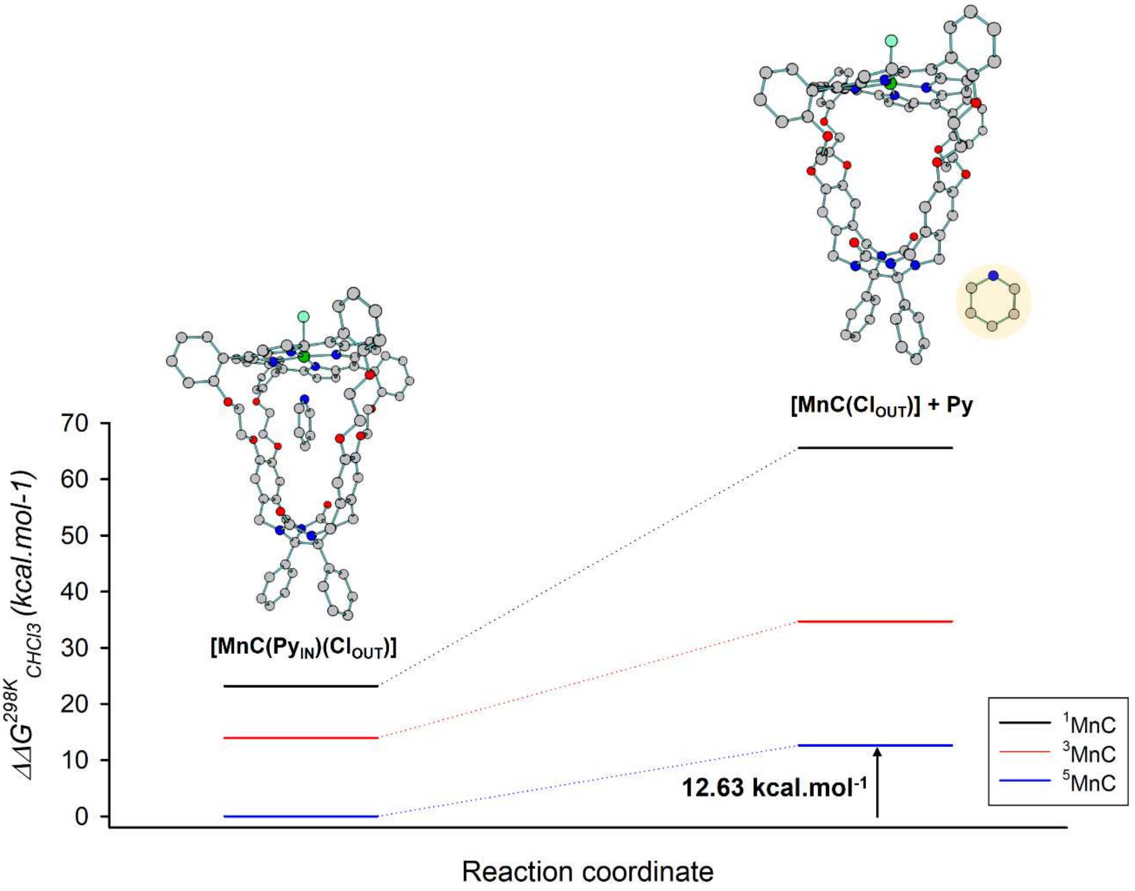

Figure S21. Free energy scheme for the inner pyridine ligand dissociation for $[Mn^{III}C(Py_{IN})(Cl_{OUT})]$. Spin states of the Mn(**III**) are written as follow: Singlet ^{1}MnC (black), Triplet ^{3}MnC (blue), Quintet ^{5}MnC (red). DFT structures are only shown for the ^{5}MnC state. H atoms are omitted for clarity.

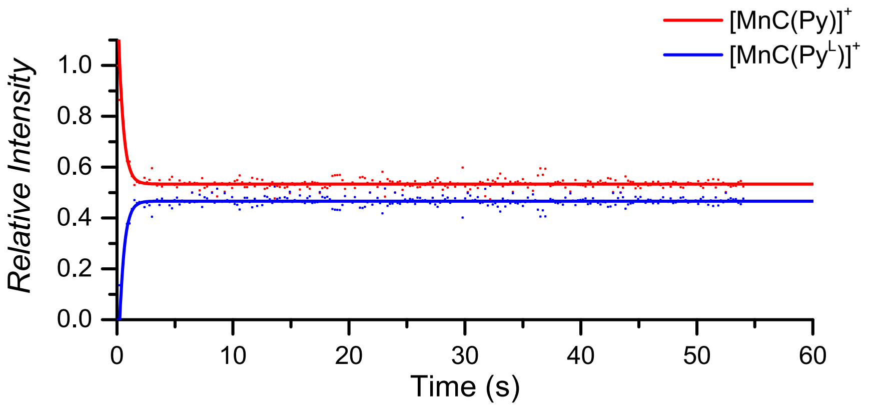

Figure S22. Relative time evolution of the intensities of $[MnC(Py)]^+$ and $[MnC(Py^L)]^+$ in a DRL experiment carried out on **MnC-PF$_6$** at 24°C in $CHCl_3$ with 100 equiv. pyridine and 100 equiv. pyridine-D5. Dots are experimental data points and lines correspond to fittings of experimental data by Equations S1 and S2.

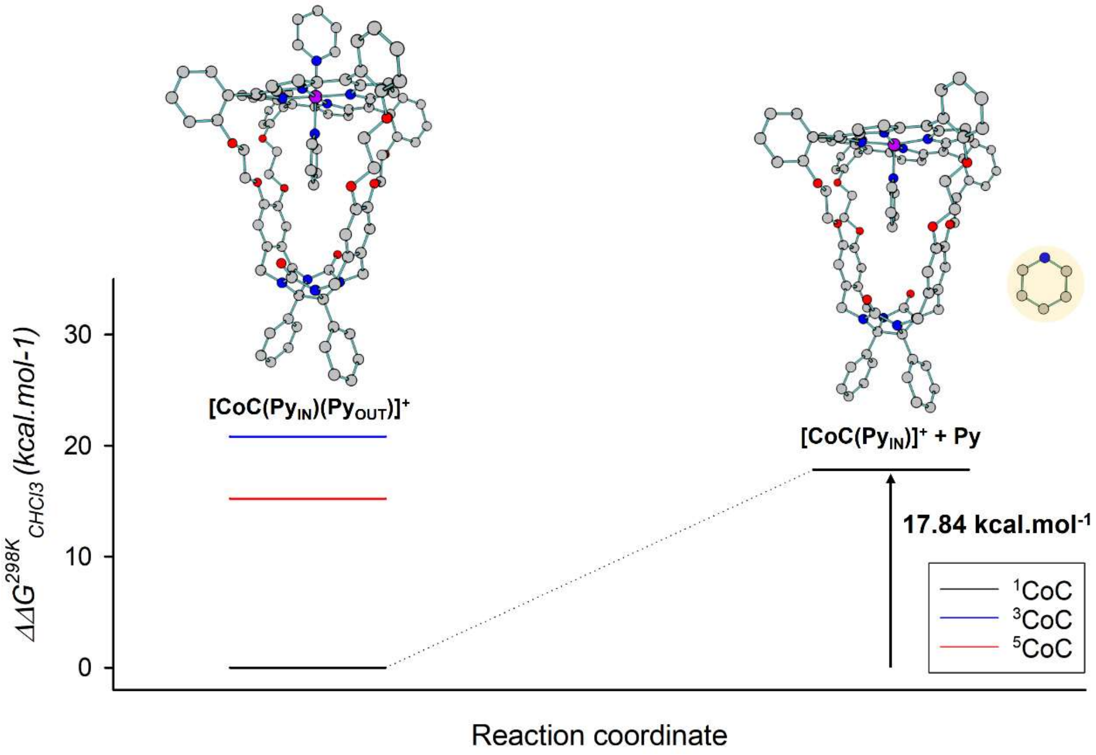

Figure S23. Free energy scheme for the outer pyridine ligand dissociation mechanism for $[Co^{III}C(Py_{OUT})(Py_{IN})]$. Spin states of the Co(III) are written as follow: Singlet ^{1}CoC (black), Triplet ^{3}CoC (blue), Quintet ^{5}CoC (red). DFT structures are only shown for the ^{1}CoC state. H atoms are omitted for clarity. Note that the calculations of the triplet and quintet states of $[CoC(Py_{IN})]^{+}$ did not converge, and are not shown.

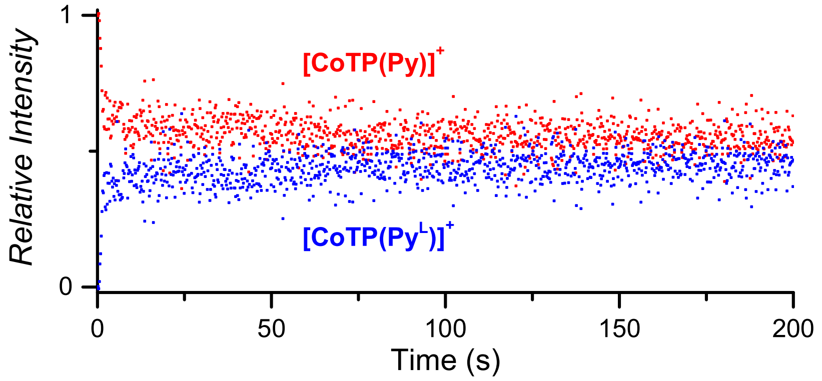

Figure S24. a) Relative time evolution of $[CoTP(Pyridine)]^{+}$ (red dots) and $[CoTP(Pyridine\text{-}D5)]^{+}$ (blue dots) in a DRL experiment recorded at 24°C with CoTP-Cl after addition of 100 equiv. pyridine and 100 equiv. pyridine-D5 in $CHCl_3$. The initial concentration of the CoTP-Cl complex was 5 μM.

Complex	Solvent	Total equiv. pyridine	T (°C)	k_{MS} (s^{-1})
**CoC-Cl**	$CHCl_3$	200	18	$1.20*10^{-4} \pm 2.36*10^{-5}$
			24	$5.10*10^{-4} \pm 3.01*10^{-5}$
			30	$2.00*10^{-3} \pm 8.18*10^{-5}$
			35	$5.59*10^{-3} \pm 6.32*10^{-4}$
			40	$1.49*10^{-2} \pm 1.05*10^{-3}$
**CoC-Cl**	CH_3CN	200	24	$2.28*10^{-5} \pm 4.36*10^{-6}$
			30	$5.39*10^{-5} \pm 5.33*10^{-6}$
			40	$2.80*10^{-4} \pm 1.63*10^{-5}$
			50	$1.18*10^{-3} \pm 3.83*10^{-4}$
**CoC*-Cl**	$CHCl_3$	200	18	$2.17*10^{-5} \pm 3.99*10^{-6}$
			24	$8.67*10^{-5} \pm 3.45*10^{-5}$
			30	$4.83*10^{-4} \pm 6.71*10^{-5}$
			35	$1.35*10^{-3} \pm 4.04*10^{-5}$
			40	$3.73*10^{-3} \pm 1.11*10^{-4}$
**CoC-PF$_6$**	$CHCl_3$	200	18	$3.05*10^{-5} \pm 8.15*10^{-6}$
			24	$1.38*10^{-4} \pm 1.31*10^{-5}$
			30	$5.49*10^{-4} \pm 1.13*10^{-4}$
			35	$1.71*10^{-3} \pm 4.62*10^{-4}$
			40	$4.41*10^{-3} \pm 1.43*10^{-3}$

Table S1. Summary of the k_{MS} measured by DRL experiments, used to calculate activation parameters for pyridine dissociation (**Table 1** of the main text). Average value for are shown, and the error range correspond to the standard deviation (n=3).

Experimental section

Mass spectrometry. Ion mobility-mass spectrometry experiments were performed with a timsToF instrument (Bruker, Germany) equipped with an ESI source. Ions were electrosprayed in positive mode with a source voltage of +5.5 kV, with a Nebulizer of 0.2 Bar, a drying gas flow of 2 L.min^{-1}, and the End Plate Offset set to 500V. Typical ion transfer voltages were quadrupole ion energy = 3 eV and collision energy = 5 eV. The mass range scanned by the ToF analyzer was *m/z* 1200-2000. TIMS experiments were performed in N_2 using the imeX Detect mode, by scanning ion mobility from 1.3 V.s.cm^{-2} to 2.08 V.s.cm^{-2}. The accumulation time was varied from 0 to 30 ms depending on the pyridine concentration, in order to maintain the maximum ion signal lower than 2.10^4 counts and thereby minimize ion activation in the ion mobility region.

The TIMS dimension was calibrated using five selected ions from the Agilent ESI LC/MS tuning mix [(1221.9906, 1.3820 V.s.cm^{-2}), (1521.9715, 1.5558 V.s.cm^{-2}), (1821.9523, 1.7286 V.s.cm^{-2}), (2121.9331, 1.8842 V.s.cm^{-2}), (2421.9140, 2.0298 V.s.cm^{-2})]. The MS dimension was calibrated linearly using the ions [1221.9906, 1521.9715, 1821.9523].

For experiments performed with the tBu_4NCl salt, the End Plate Offset was varied from 0-80V, the nebulizer set to 0 Bar and the accumulation time was set to 50 ms to account for ion suppression.

Data analysis. Ion chromatograms and ion mobilograms were extracted with a width of 0.02 Da. The ion intensities of *m/z* 1485 ($[CoC(Py^L)]^+$), *m/z* 1536 ($[CoC^*(Py^L)]^+$), *m/z* 1564 ($[CoC(Py)(Py^L)]^+$), *m/z* 1569 ($[CoC(Py^L)_2]^+$), *m/z* 1620 ($[CoC^*(Py)(Py^L)_2]^+$), and *m/z* 1625 ($[CoC^*(Py^L)_2]^+$) were corrected to remove the isotopic contribution from the non-labeled compounds *m/z* 1480 ($[CoC(Py)]^+$), *m/z* 1559 ($[CoC(Py)_2]^+$) and *m/z* 1615 ($[CoC^*(Py)_2]^+$) respectively.

Fitting of the DRL curves. The relative abundance of $[CoC(Py)]^+$ and $[CoC(Py^L)]^+$ were fitted with Equations S1 and S2:

$$[CoC(Py)]_t = e^{-k_{MS}.t} + A\left(1 - e^{-k_{MS}.t}\right) - L_0 \quad \text{(Equation S1)}$$

$$[CoC(Py^L)]_t = A^L(1 - e^{-k_{MS}.t}) + L_0 \quad \text{(Equation S2)}$$

With L_0 being the amount of $[CoC(Py^L)]$ at t = 0. This parameter has been added to account for eventual carryovers of pyridine-D5 from one experiment to another. In most of the experiments performed, L_0 was comprised between 0 and 0.05. With these equations, $[CoC(Py)]_{eq} = A - L_0$ and $[CoC(Py^L)]_{eq} = A^L + L_0$.

Computational details. DFT calculations were carried out at the B3LYP-D3/def2svp level with the Gaussian 16 package. The SMD model was used to account for implicit chloroform solvation. All reported structures correspond to potential energy surface minima as confirmed by analyses of the corresponding Hessian matrixes. Reported energies include zero-point vibrational energy corrections and thermal corrections calculated at the same level of theory. Reported ΔG values also include a correction of (1.9 Δn) kcal mol^{-1} to account for the change in number of moles (n) for reactions involving dissociation of a ligand.

Preparation of reaction mixtures. Reaction mixtures for DRL experiments were prepared by mixing stock solutions of the porphyrin complex and pyridine in a glass vial under continuous stirring to obtain a concentration of 4 µM of porphyrin complex, and 100 equiv. pyridine. The glass vial was placed in a home-made Peltier heater/cooler to control the reaction temperature (**Figure S25**), and the reaction mixture was infused into the ESI source of the timsToF instrument via a silica capillary by applying an overpressure of N_2 (approx. 2.5 psi). After two

minutes, a minimal volume (80µL) of pyridine-D5 is added to the reaction mixture with a Hamilton syringe. Final concentrations (for addition of 100 equiv. pyridine and 100 equiv. pyridine-D5) : 3.82 µM of porphyrin compound, 380 µM pyridine, 380 µM pyridine-D5.

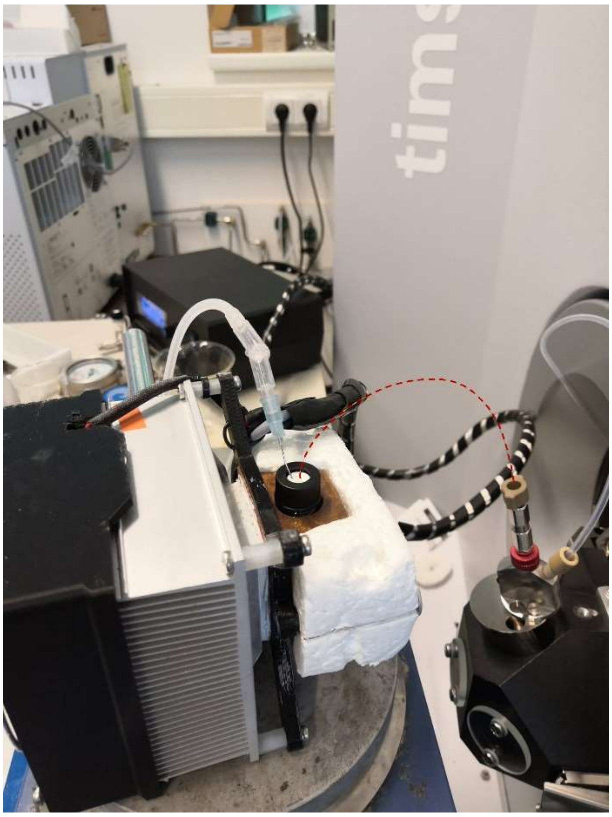

Figure S25. Picture of the experimental setup in which the DRL reaction mixture is contained in the glass vial placed intro a home-made Peltier heater/cooler. A flow of N_2 is applied to the reaction mixture to infuse the solution from the vial to the ESI source through a silica capillary highlighted by red stripes.

X-ray crystallography. Reflections were measured on a Bruker D8 Quest diffractometer with sealed tube and Triumph monochromator (λ = 0.71073Å). Software package used for the intensity integration was Saint (v8.40a).[1] Absorption correction was performed with SADABS.[2] The structures were solved with direct methods using SHELXT-2014/5.[3] Least-squares refinement was performed with SHELXL-2018/3[4] against $|F_h^o|^2$of all reflections. Non-hydrogen atoms were refined freely with anisotropic displacement parameters. Hydrogen atoms were placed on calculated positions or located in difference Fourier maps. All calculated hydrogen atoms were refined with a riding model.

Crystal structure and structure refinement of CoC-Cl

Single crystals were grown by slow diffusion of *n*-heptane into a chloroform solution of the compound.

General information

Identification code	**CoC-Cl** / p2124a
Crystal colour	blue
Crystal dimensions [mm] / shape	0.07 x 0.12 x 0.62 / needle
Crystallization solvent	Chloroform

Empirical formula		$C_{94}H_{72}CoN_{10}O_{10}$, 6($CHCl_3$), Cl
Formula weight	[g/mol]	2312.20

Crystal Data

Crystal system	Monoclinic
Space group	*P*2$_1$/c (#14)
Unit cell dimensions	
a, b, c [Å]	16.1693(4), 23.4065(6), 27.4555(8)
α, β, γ [°]	90, 94.7126(11), 90
Volume [Å^3]	10355.9(5)
Z	4
Density (calculated) [g/cm^3]	1.483
Absorption coefficient (MoKα) [mm^{-1}]	0.719
F(000)	4712

Data Collection

Temperature during experiment [K]	200
Wavelength [Å]	0.71073
θ Min-Max [°]	1.9, 28.3
Index range	-21≤ h ≤20 ; -30 ≤ k ≤31 ; 36 ≤ l ≤35
Tot., Uniq. Data, R(int)	112760, 25748, 0.049
Observed Data [I > 2.0 σ(I)]	17972

Refinement

Nref, Npar	25748, 1299
R, wR2, S	0.0994, 0.2834, 1.05
Min. and Max. Resd. Dens. [e/ Å^3]	-1.47, 2.15

Crystal structure and structure refinement of [CoC(Py)$_2$]Cl

Single crystals were grown by slow diffusion of *n*-heptane into a chloroform solution of the complex.

General information

Identification code		**[CoC(Py)$_2$]Cl** / p2126_sq
Crystal colour		red
Crystal dimensions [mm] / shape		0.05 x 0.34 x 0.39 / plate
Crystallization solvent		Chloroform
Empirical formula		$C_{84}H_{62}ClCoN_8O_{10}$, 3($CHCl_3$) [+SOLVENT]
Formula weight	[g/mol]	1795.90

Crystal Data

Crystal system	Triclinic
Space group	*P*-1 (#2)
Unit cell dimensions	
a, b, c [Å]	12.0482(6), 18.3543(9), 22.3068(11)
α, β, γ [°]	83.0587(17), 77.5078(17), 83.6713(18)
Volume [Å^3]	4762.7(4)
Z	2
Density (calculated) [g/cm^3]	1.252
Absorption coefficient (MoKα) [mm^{-1}]	0.517
F(000)	1840

Data Collection

Temperature during experiment [K]	200
Wavelength [Å]	0.71073
θ Min-Max [°]	1.9, 31.7
Index range	-17≤ h ≤17 ; -27 ≤ k ≤18 ; 32 ≤ l ≤32
Tot., Uniq. Data, R(int)	137129, 31868, 0.059
Observed Data [I > 2.0 σ(I)]	17815

Refinement

Nref, Npar	31868, 1045
R, wR2, S	0.0999, 0.3350, 1.03
Min. and Max. Resd. Dens. [e/ Å ^{3}]	-1.46, 1.80

Sqeeze

Solvent Accessible Volume [Å ^{3}]	1014
Electrons Found in S.A.V. [e]	343

Synthesis Procedures

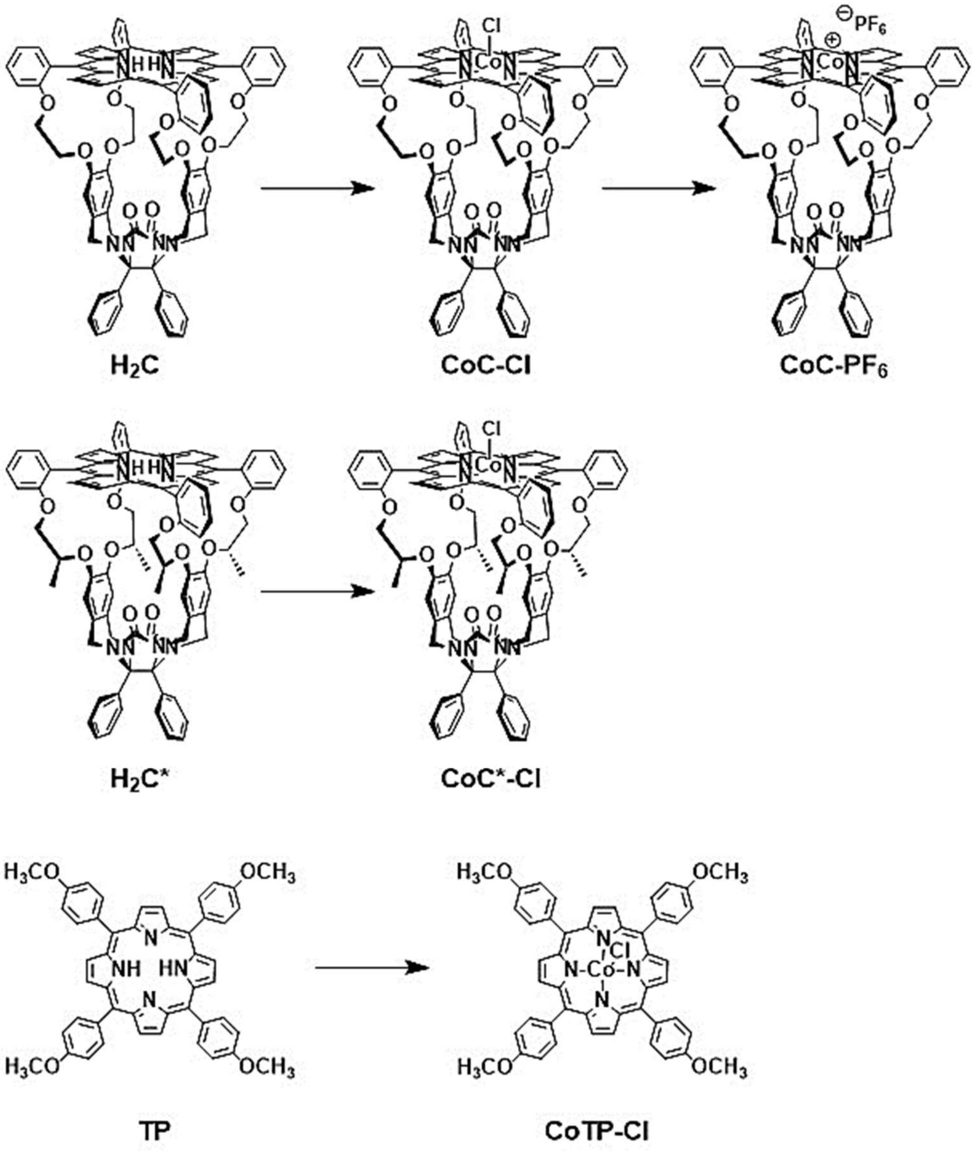

Scheme S1. Synthesis schemes of the various cobalt porphyrin derivatives.

General. Porphyrin cages **H_2C**,[5] **H_2C***,[6] **MnC-Cl**[7] and **MnC-PF_6**[8] were synthesized according to literature procedures. Other solvents and reagents were obtained from commercial suppliers and used without further purification. Reactions were followed by using thin-layer chromatography (TLC) on silica gel-coated plates (Merck 60 F254). Melting points were taken on a polarization microscope with a programmable hot-stage. NMR spectra were recorded at 298 K on a Bruker Avance III 500 spectrometer (500 MHz) equipped with a Prodigy BB cryoprobe and on a Bruker Avance III 400 spectrometer (400 MHz) equipped with a BBFO probe. ^{1}H NMR chemical shifts (δ) are given in parts per million (ppm) and were referenced to tetramethylsilane (TMS, δ = 0.00 ppm). Carbon chemical shifts (δ) are given in ppm and were referenced to to tetramethylsilane (TMS, δ = 0.00 ppm). Data for ^{1}H NMR spectra are reported as follows: chemical shift (multiplicity, coupling constant, integration, assignment). Data for ^{13}C NMR spectra are reported as follows: chemical shift. Multiplicities are abbreviated as s (singlet), d (doublet), t (triplet), q (quartet), p (quintet), m (multiplet), b (broad). Coupling constants are reported as J values in Hertz (Hz). Assignments were based on ^{1}H, ^{13}C, COSY, HSQC, and HMBC NMR spectra. High Resolution Mass Spectra were recorded on a Bruker timsToF

equipped with an ESI ion source. UV-Vis spectra spectra were recorded at 298 K on a JASCO J-815 CD spectrophotometer (2 mm quartz cell).

Synthesis of CoC-Cl. To an argon-purged solution of the corresponding free base porphyrin cage **H_2C** (182 mg, 0.135 mmol) in chloroform (20 mL), methanol (20 mL) and triethylamine (1 mL) was added $Co(OAc)_2.4H_2O$ (215 mg, 0.863 mmol). The mixture was heated at reflux under argon for 64 h. After cooling, the solvent was evaporated and the residue redissolved in $CHCl_3$ (50 mL). The organic layer was subsequently extracted with brine (2 × 100 mL) and water (100 mL) and evaporated to dryness. The crude product was purified by column chromatography (silica 60H, 1% MeOH in $CHCl_3$ (v/v)) and the first red fraction was collected and evaporated to dryness. The product was dissolved in a minimal amount of chloroform, the solution was filtered, and then added to rapidly stirred *n*-heptane (20 mL). The formed precipitate was collected by centrifugation and dried under vacuum. It was subsequently dissolved in chloroform (5 mL), methanol (3 mL) and 12N aqueous HCl (2 mL) and the solution was stirred in air for 3 h. $CHCl_3$ (50 mL) was added and the organic layer was extracted with water (2 × 100 mL) and evaporated to dryness. The product was dissolved in a minimal amount of chloroform, the solution was filtered, and then added to rapidly stirred *n*-heptane (20 mL). The formed precipitate was collected by centrifugation and dried under vacuum. Yield: 122 mg (63%) of **CoC-Cl** as a red solid. Single crystals suitable for X-ray analysis were grown by slow diffusion of *n*-heptane in a $CDCl_3$ solution of the compound. M.p. > 300 °C (dec). ^{1}H NMR (500 MHz, $CDCl_3$) δ 8.57 (s, 4H, β-pyrroleH), 8.44 (s, 4H, β-pyrroleH), 8.02 (dd, *J* = 7.4, 1,7 Hz, 4H, ArH), 7.76 (td, *J* = 8.4, 1,7 Hz, 4H, ArH), 7.31 (t, *J* = 7.4 Hz, 4H, ArH), 7.29 (d, *J* = 8.2 Hz, 4H, ArH), 7.00-6.92 (m, 6H, ArH), 6.84-6.77 (m, 4H, ArH), 6.21 (s, 4H, ArH), 4.40-4.33 (m, 4H, CH_2O), 4.25 (d, *J* = 15.9 Hz, 4H, NCH_2Ar), 4.20-4.13 (m, 4H, CH_2O), 3.77 (d, *J* = 15.9 Hz, 4H, NCH_2Ar), 3.64-3.56 (m, 4H, CH_2O), 3.55-3.46 (m, 4H, CH_2O) ppm. ^{13}C{^{1}H} NMR (125 MHz, $CDCl_3$) δ 157.13, 156.18, 150.72, 148.44, 146.52, 133.45, 133.04, 132.97, 132.83, 131.71, 129.96, 129.93, 128.62, 128.52, 128.10, 120.21, 115.33, 111.71, 84.86, 67.30, 66.59, 44.45 ppm. UV-vis ($CHCl_3$) λ/nm (log(ε/$M^{-1}\cdot cm^{-1}$)) 407 (4.70), 425 (sh, 4.63), 547 (3.84); MALDI-TOF: *m/z* = 1401 (M – Cl)$^+$. HRMS: *m/z* 1442.4160 [M – Cl + CH_3CN]$^+$ (expected 1442.4181 for [$C_{86}H_{65}N_9O_{10}Co$]$^+$).

Synthesis of CoC-PF$_6$. A suspension of **CoC-Cl** (34.0 mg, 23.6 μmol) and $AgPF_6$ (6.0 mg, 24 μmol) in freshly distilled CH_2Cl_2 (2 mL) was stirred under argon in the dark for 16 h. Then, 5.0 mg of $AgPF_6$ (19 μmol) were added and stirring was continued for 2h. The suspension was filtered over a thin layer celite, which was washed with a mixture of $CHCl_3$ and MeOH (9:1, v/v) until the washings remained colorless. The filtrate was evaporated to dryness and the crude product was purified by column chromatography (silica 60H, $CHCl_3$/MeOH 9:1, v/v). The red fraction was collected and evaporated to dryness. The product was dissolved in a minimal amount of chloroform, the solution was filtered, and then added to rapidly stirred *n*-heptane (20 mL). The formed precipitate was collected by centrifugation and dried under vacuum. Yield: 18.0 mg (49%) of **CoC-PF$_6$** as a red solid. M.p. > 300 °C (dec). ^{1}H NMR (500 MHz, DMSO-d6) δ 9.19 (s, 4H, β-pyrroleH), 9.05 (s, 4H, β-pyrroleH), 8.09 (d, *J* = 7.4 Hz, 4H, ArH), 7.94 (t, *J* = 8.4 Hz, 4H, ArH), 7.65 (d, *J* = 8.2 Hz, 4H, ArH), 7.52 (t, *J* = 7.4 Hz, 4H, ArH), 7.08-6.98 (m, 6H, ArH), 6.92-6.74 (m, 4H, ArH), 6.23 (s, 4H, ArH), 4.44-4.26 (m, 8H, CH_2O), 4.18 (d, *J* = 15.9 Hz, 4H, NCH_2Ar), 3.85-3.72 (m, 4H, CH_2O), 3.67 (d, *J* = 15.9 Hz, 4H, NCH_2Ar), 3.33-3.21 (m, 4H, CH_2O) ppm. ^{13}C{^{1}H} NMR (125 MHz, DMSO-d6) δ 158.50, 156.57, 145.08, 144.48, 144.24, 135.29, 134.99, 134.58, 134.18, 129.97, 129.88, 129.36, 128.86, 128.67, 128.25, 120.42, 116.88, 113.90, 112.42, 84.63, 67.05, 66.82, 44.10 ppm. UV-vis ($CHCl_3$) λ/nm (log(ε/$M^{-1}\cdot cm^{-1}$)) 412 (4.85), 422 (4.85), 543 (3.99); HRMS: *m/z* 1442.4144 [M – PF_6 + CH_3CN]$^+$ (expected 1442.4181 for [$C_{86}H_{65}N_9O_{10}Co$]$^+$).

Synthesis of CoC*-Cl (*S,S,S,S*-enantiomer). Starting from the corresponding free base porphyrin cage **H_2C*** (*S,S,S,S*-enantiomer, 19.0 mg, 0.0136 mmol) this compound was synthesized as described for **CoC-Cl**. Yield: 13.8 mg (70%) of a red solid. M.p. > 300 °C (dec). ^{1}H NMR (500 MHz, $CDCl_3$) : the compound

is present as two isomers, a major isomer (88%) with the Cl-ligand coordinated to the outside of the cavity, and a minor isomer (12%) with the Cl-ligand coordinated to the inside. In the following only the NMR-signals of the major isomer will be reported : δ 8.73-8.54 (m, 8H, β-pyrroleH), 8.26 (d, *J* = 7.0 Hz, 2H, ArH), 8.17 (d, *J* = 6.5 Hz, 2H, ArH) 7.81-7.72 (m, 4H, ArH), 7.47-7.36 (m, 4H, ArH), 7.28 (d, *J* = 8.5 Hz, 4H, ArH), 7.06-6.77 (m, 10H, ArH), 6.28 (s, 2H, ArH), 6.10 (s, 2H, ArH), 4.21 (d, *J* = 15.5 Hz, 2H, NCH_2Ar), 4.17 (d, *J* = 15.5 Hz, 2H, NCH_2Ar), 4.10-4.02 (m, 2H, CH_2O), 4.02-3.94 (m, 2H, $CH(CH_3)O$), 3.90-2.83 (m, 2H, CH_2O), 3.81- 3.71 (m, 2H, CH_2O), 3.74 (d, *J* = 15.5 Hz, 2H, NCH_2Ar), 3.72 (d, *J* = 15.5 Hz, 2H, NCH_2Ar), 3.58-3.49 (m, 2H, CH_2O), 3.31-3.21 (m, 2H, $CH(CH_3)O$), −0.33 (d, *J* = 5.5 Hz, 6H, CH_3), −0.52 (d, *J* = 5.5 Hz, 6H, CH_3) ppm; $^{13}C\{^1H\}$ NMR (125 MHz, $CDCl_3$) δ 157.59, 157.40, 157.11, 150.73, 149.76, 149.25, 148.48, 147.30, 147.04, 146.15, 143.92, 133.76, 133.55, 133.40, 133.25, 133.06, 132.86, 132.25, 131.78, 131.16, 130.02, 129.47, 129.03, 128.66, 128.51, 128.44, 128.17, 127.75, 127.49, 125.29, 122.24, 122.14, 115.85, 113.93, 113.50, 111.54, 84.96, 72.67, 72.49, 72.40, 71.36, 71.01, 70.52, 44.60, 44.42, 43.98, 43.84, 17.00, 15.26, 13.69, 12.68 ppm. UV-vis ($CHCl_3$) λ/nm (log(ε/$M^{-1}\cdot cm^{-1}$)) 410 (sh, 4.69), 432 (4.65), 547 (3.82); HRMS: *m/z* 1498.4781 $[M - Cl + CH_3CN]^+$ (expected 1498.4807 for $[C_{90}H_{73}N_9O_{10}Co]^+$).

Synthesis of CoTP-Cl. Tetrakis-(*meso*-*p*-methoxyphenyl) porphyrin **TP** (60 mg, 0.082 mmol) and $Co(OAc)_2.4H_2O$ (92 mg, 0.37 mmol) were suspended in an argon-purged mixture of $CHCl_3$ (4 mL), MeOH (2 mL) and Et_3N (0.5 mL). The mixture was heated at reflux under argon for 64 h. After cooling, the solvent was evaporated to dryness. The residue was suspended in $CHCl_3$ (20 mL), the organic layer was extracted with water (2 x 50 mL) and evaporated to dryness. The formed Co(II) porphyrin derivative was purified by flash column chromatography (silica 60A, $CHCl_3$). The product was dissolved in a mixture of $CHCl_3$ (6 mL) and MeOH (2 mL) and a droplet of 12N aqueous HCl was added. The solution was stirred open to air until TLC analysis ($CHCl_3$) indicated consumption of the starting material (after about 1h), and then extracted with water (2 x 50 mL). The organic layer was evaporated to dryness, and the crude product was recrystallized from $CHCl_3$/*n*-heptane (1:10, v/v) to yield 35 mg (52%) of **CoTP-Cl** as an orange-red solid. M.p. > 300°C. ^1H NMR (400 MHz, $CDCl_3$/DMSO-d6 95:5, v/v) δ 9.08 (s, 8H, β-pyrroleH), 8.11 (d, 8H, ArH, *J* = 8.0 Hz), 7.28 (d, 8H, ArH, *J* = 8.0 Hz), 4.09 (s, 12H, OCH_3). $^{13}C\{^1H\}$ NMR (125 MHz, $CDCl_3$/DMSO-d6 95:5, v/v) δ 159.34, 144.40, 135.24, 134.15, 119.46, 112.33, 55.56. UV-vis ($CHCl_3$) λ/nm (log(ε/$M^{-1}\cdot cm^{-1}$)) 418 (4.92), 539 (4.25); HRMS: *m/z* 791.2049 $[M - Cl]^+$ (expected 791.2063 for $[C_{48}H_{36}N_4O_4Co]^+$).

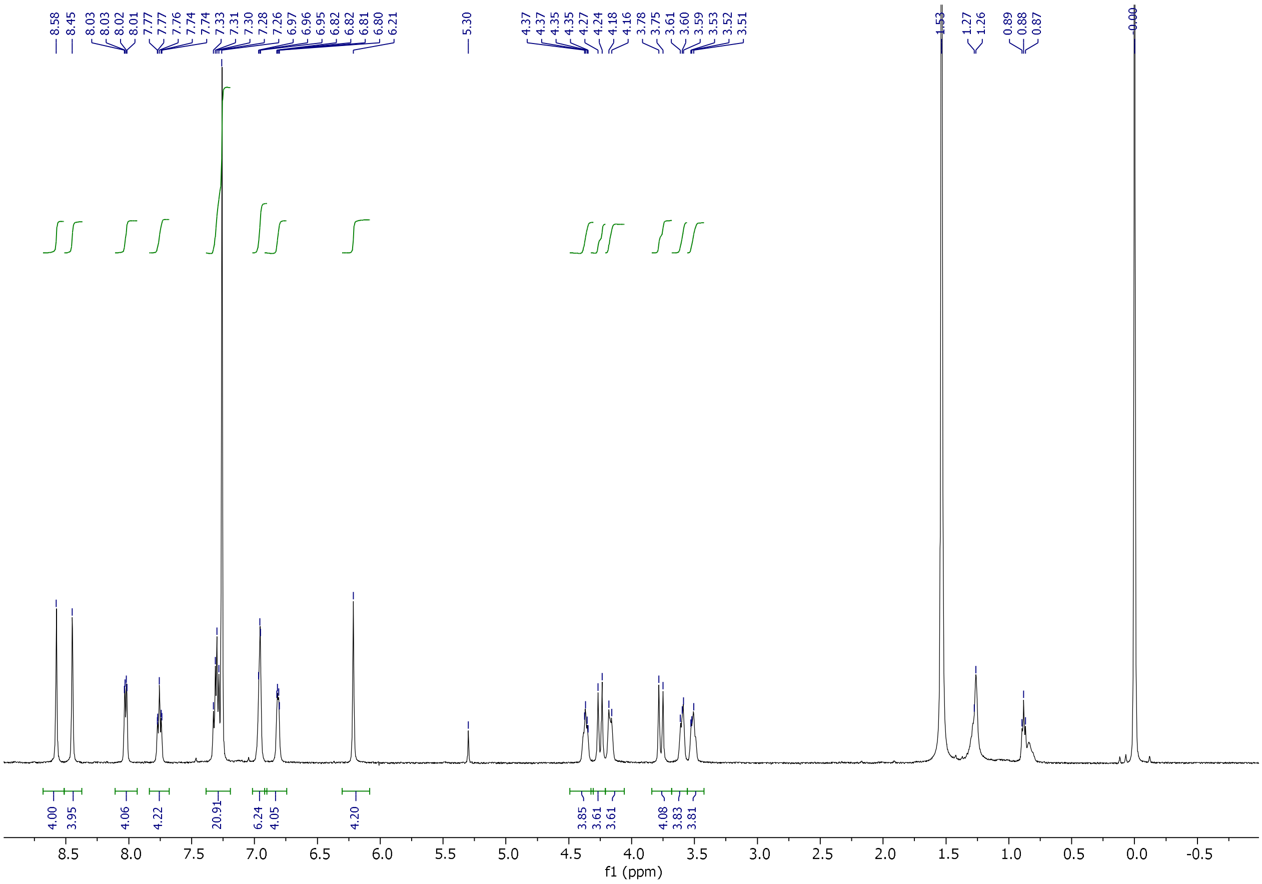

Figure S26 ^{1}H NMR ($CDCl_3$, 500 MHz) spectrum of **CoC-Cl** (c = 1.79 mM).

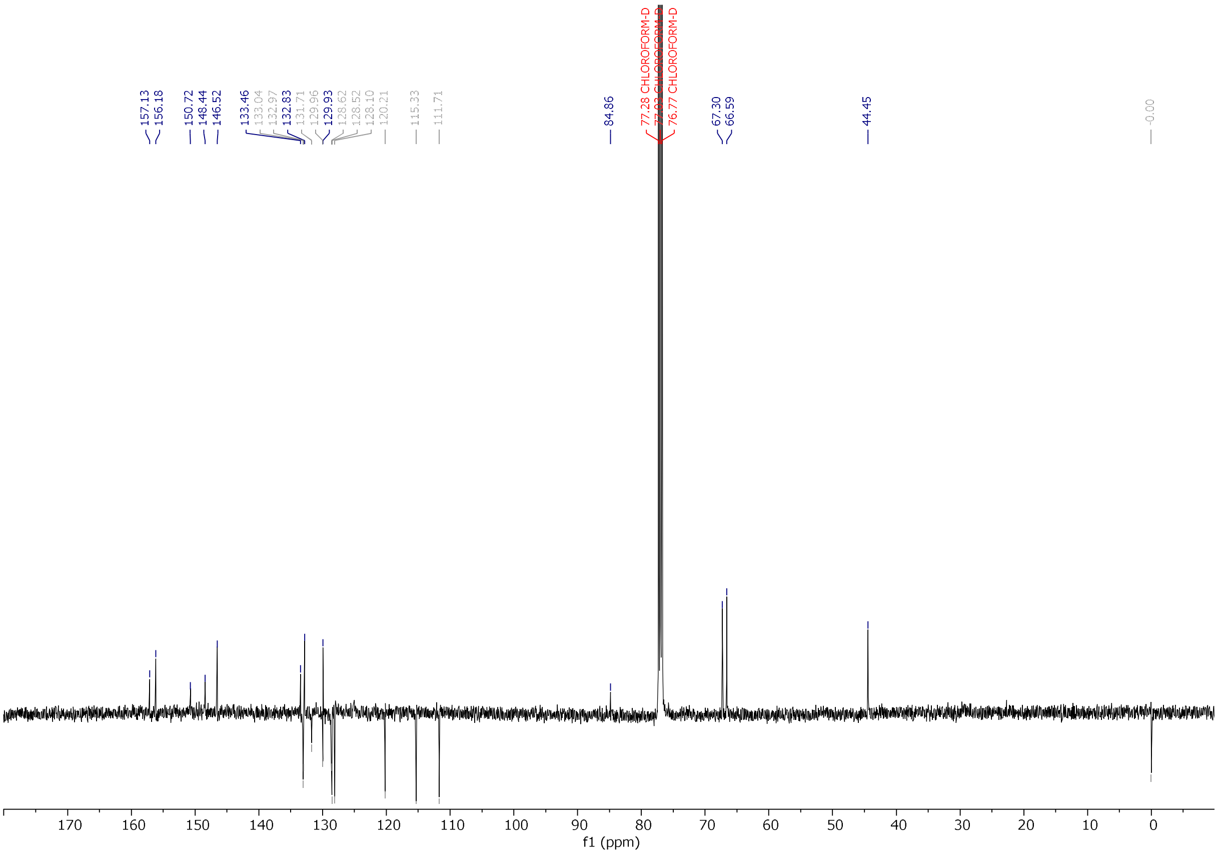

Figure S27 $^{13}C\{^{1}H\}$ NMR ($CDCl_3$, 125 MHz) spectrum of **CoC-Cl** (c = 1.79 mM).

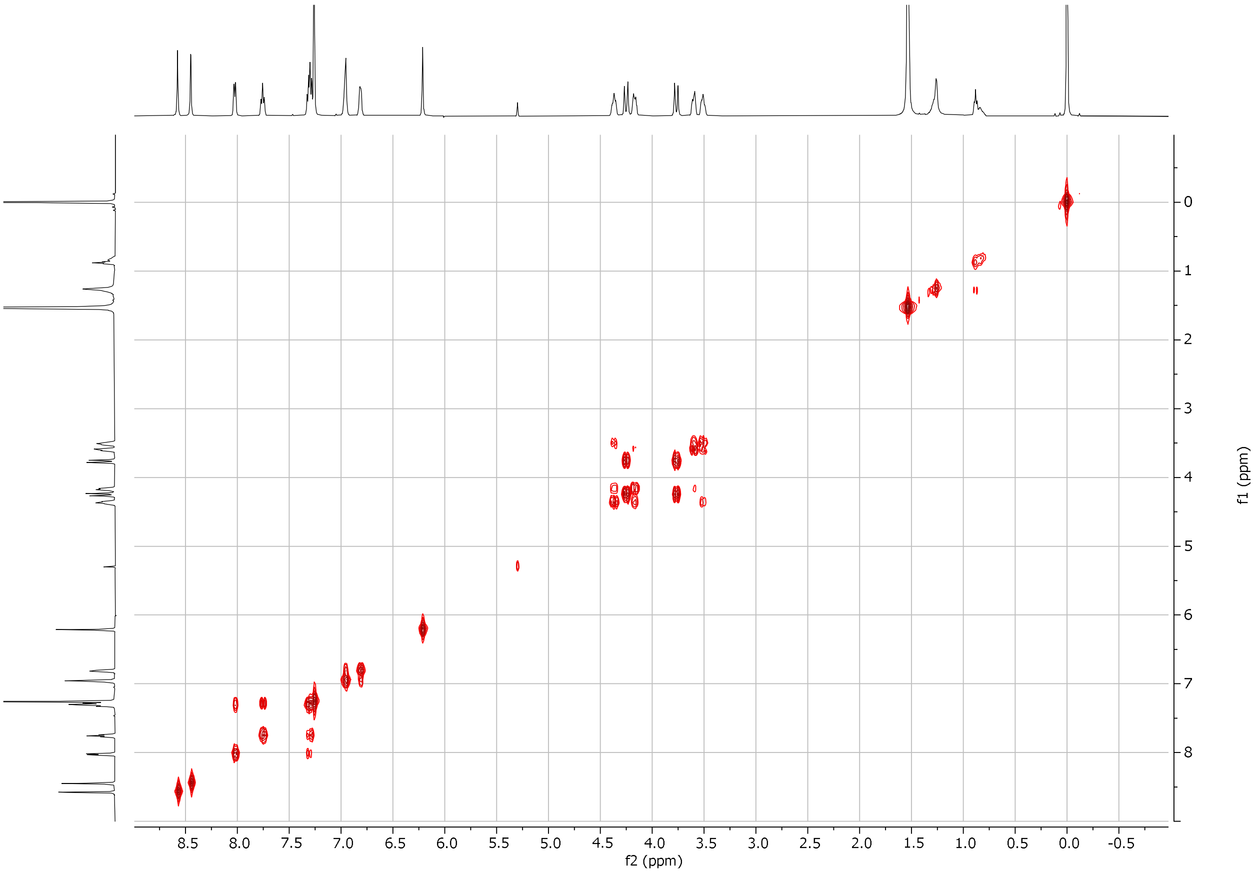

Figure S28 ^{1}H-^{1}H COSY NMR ($CDCl_3$, 500 MHz) spectrum of **CoC-Cl** (c = 1.79 mM).

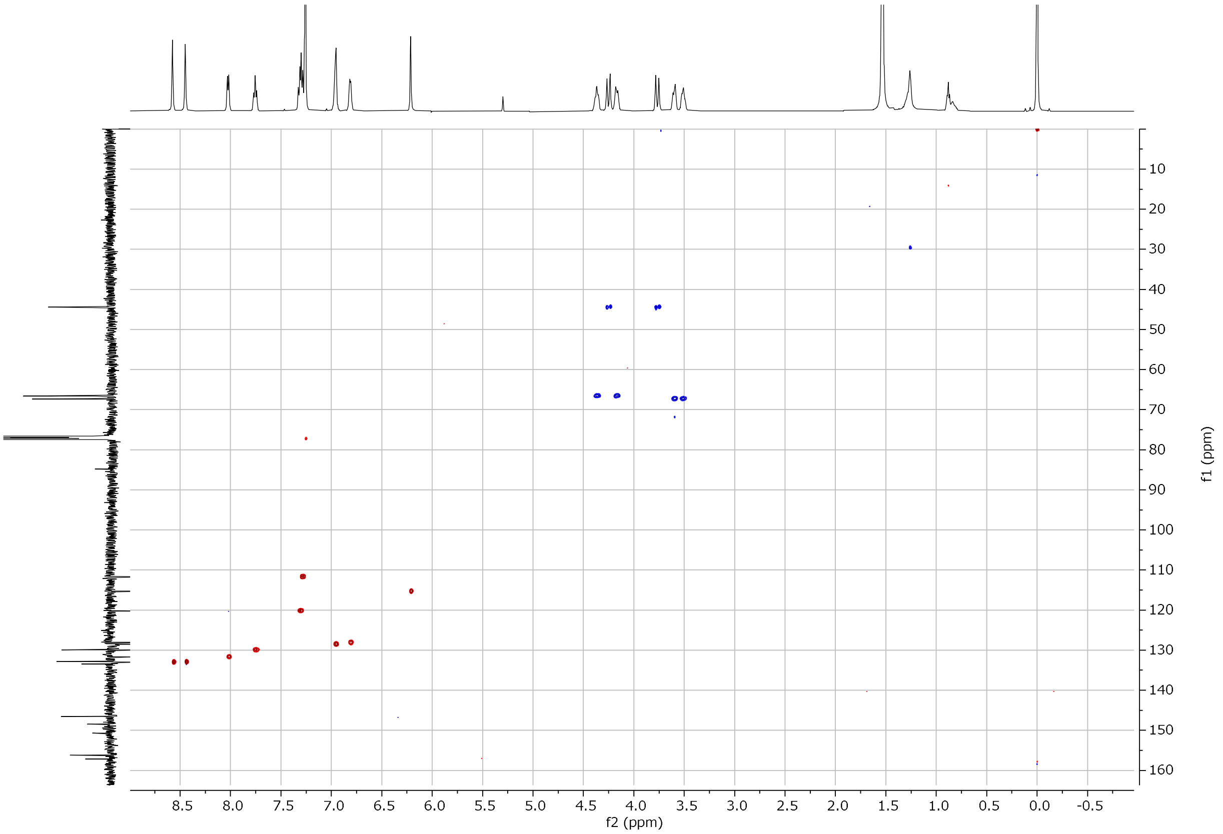

Figure S29 ^{1}H-^{13}C HSQC NMR ($CDCl_3$, 500-125 MHz) spectrum of **CoC-Cl** (c = 1.79 mM).

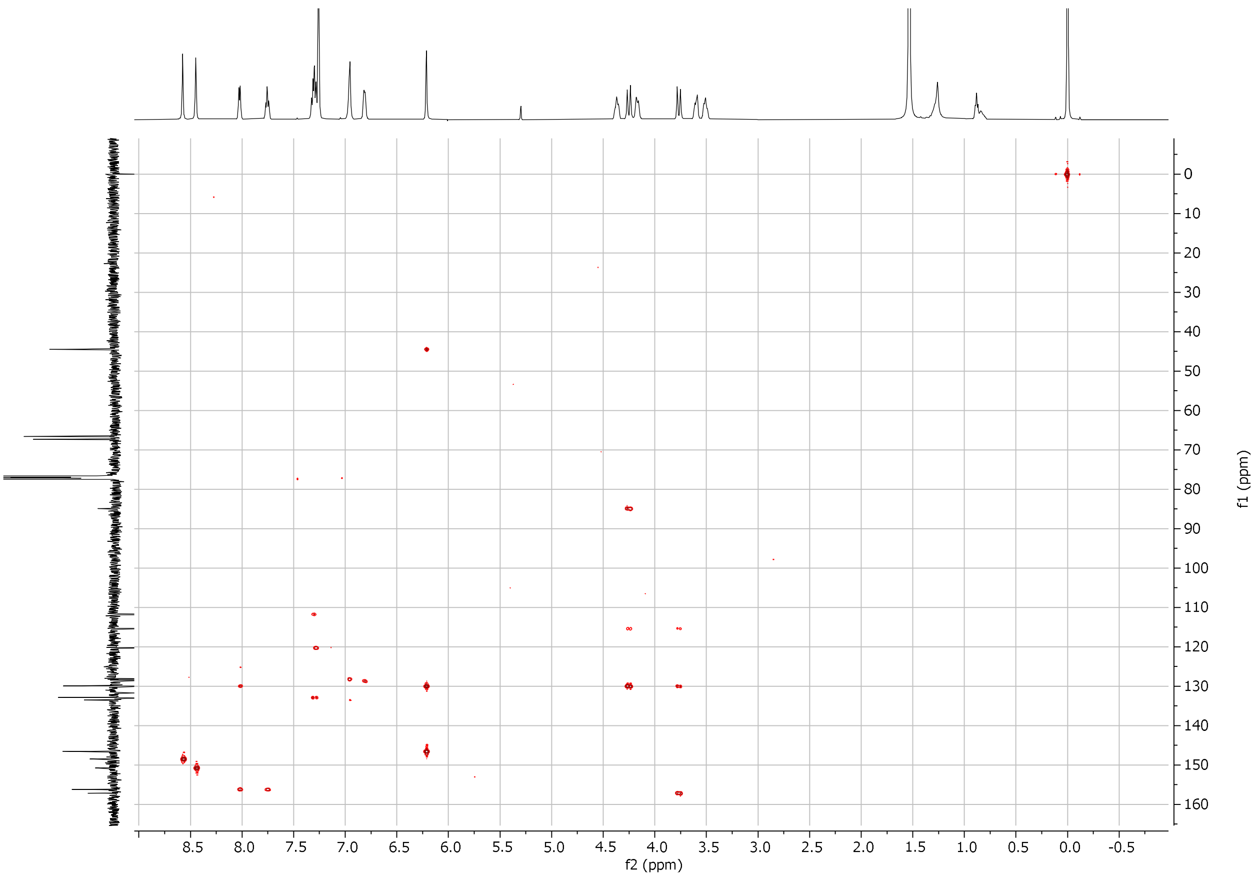

Figure S30 ^{1}H-^{13}C HMBC NMR ($CDCl_3$, 500-125 MHz) spectrum of **CoC-Cl** (c = 1.79 mM).

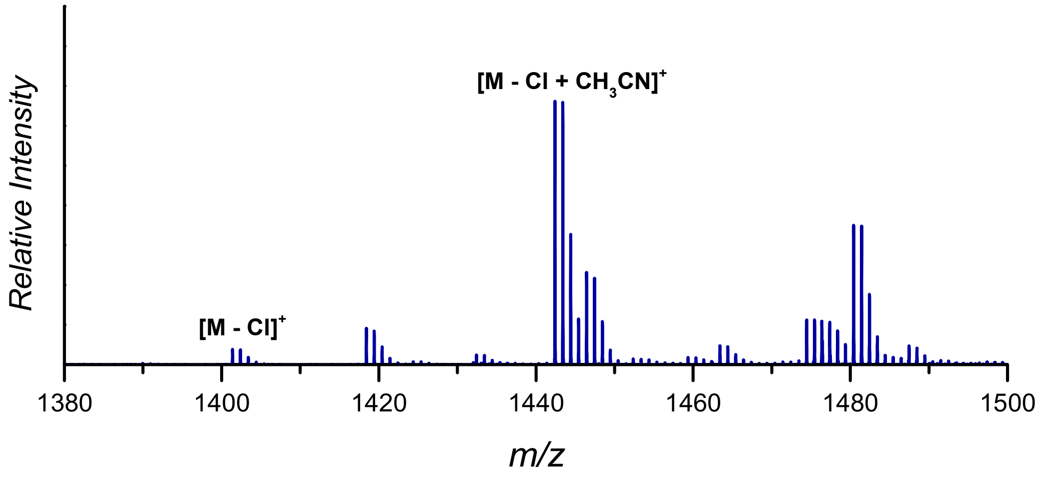

Figure S31 High resolution mass spectrum of **CoC-Cl** (c = 4 µM, CH_3CN).

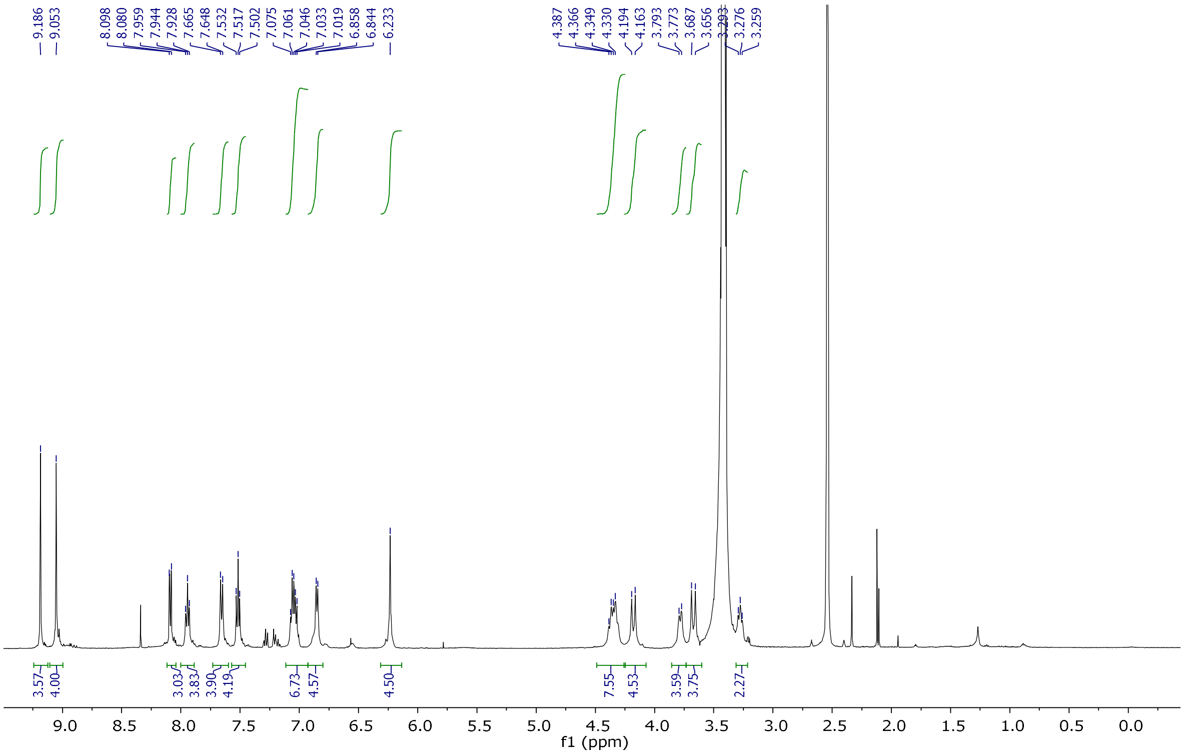

Figure S32 ^{1}H NMR (DMSO-d6, 500 MHz) spectrum of **CoC-PF$_6$** (c = 1.4 mM).

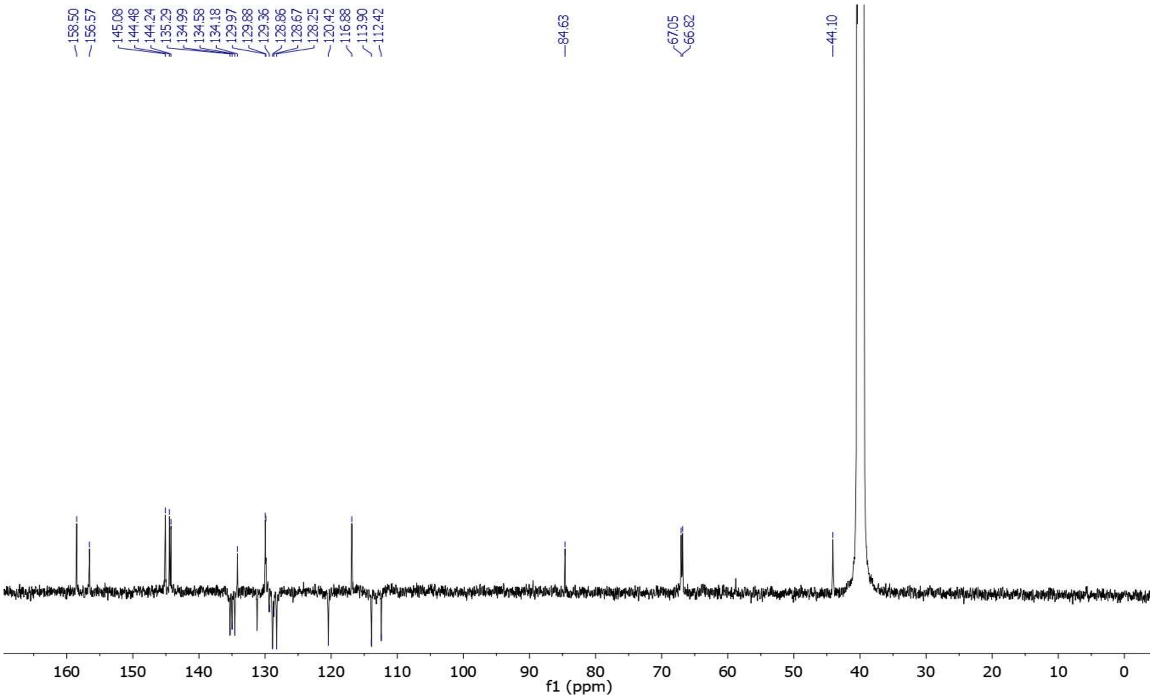

Figure S33 $^{13}C\{^{1}H\}$ NMR (DMSO-d6, 125 MHz) spectrum of **CoC-PF$_6$** (c = 1.4 mM).

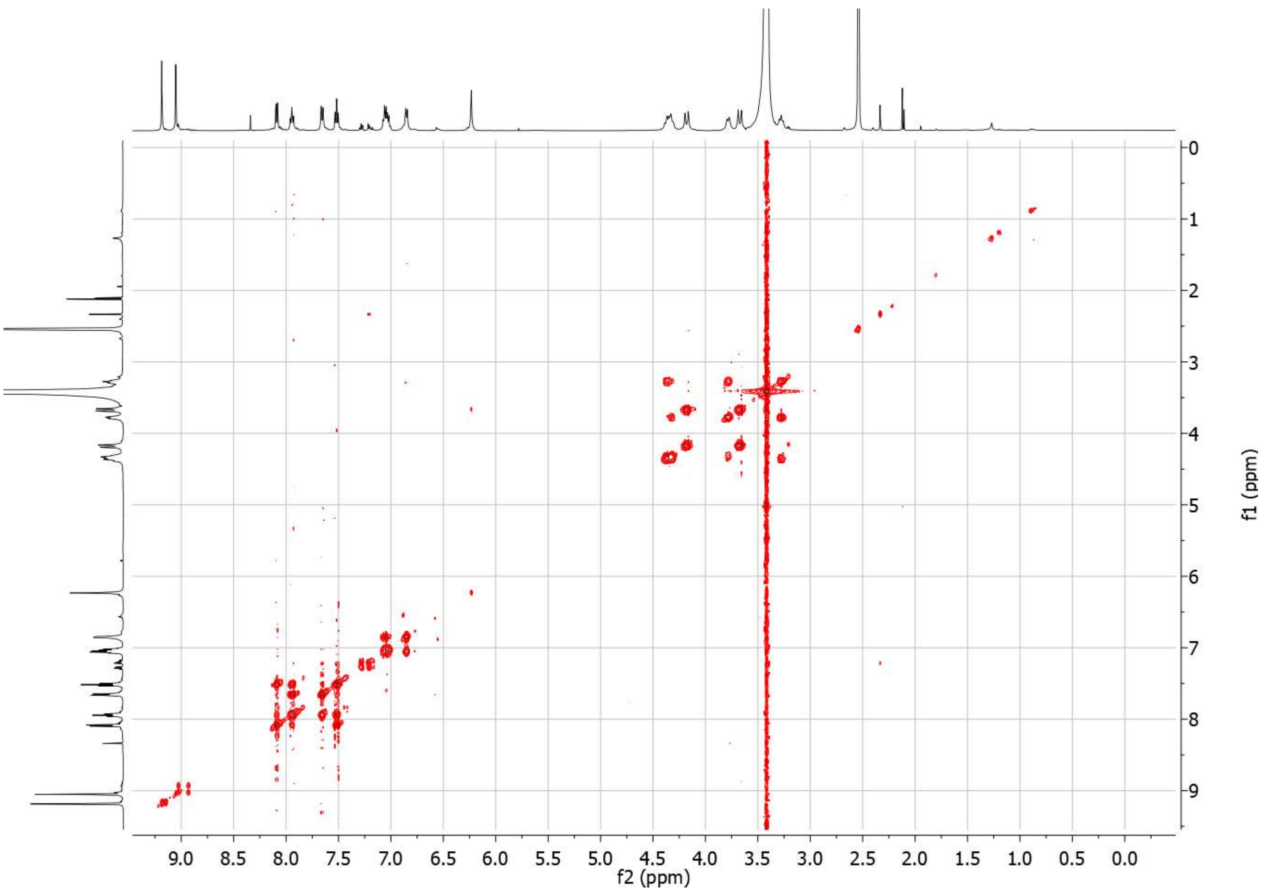

Figure S34 ^{1}H-^{1}H COSY NMR (DMSO-d6, 125 MHz) spectrum of **CoC-PF$_6$** (c = 1.4 mM).

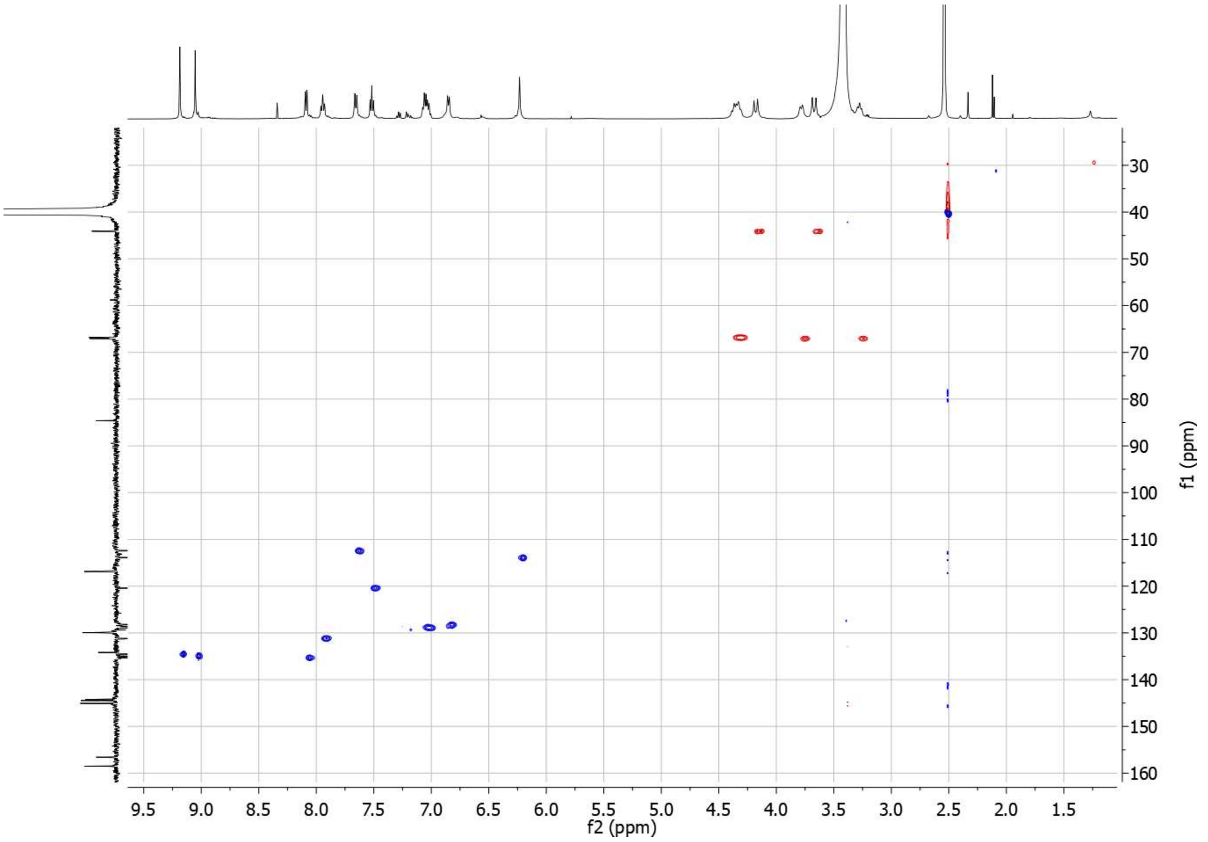

Figure S35 ^{1}H-^{13}C HSQC NMR (DMSO-d6, 125 MHz) spectrum of **CoC-PF$_6$** (c = 1.4 mM).

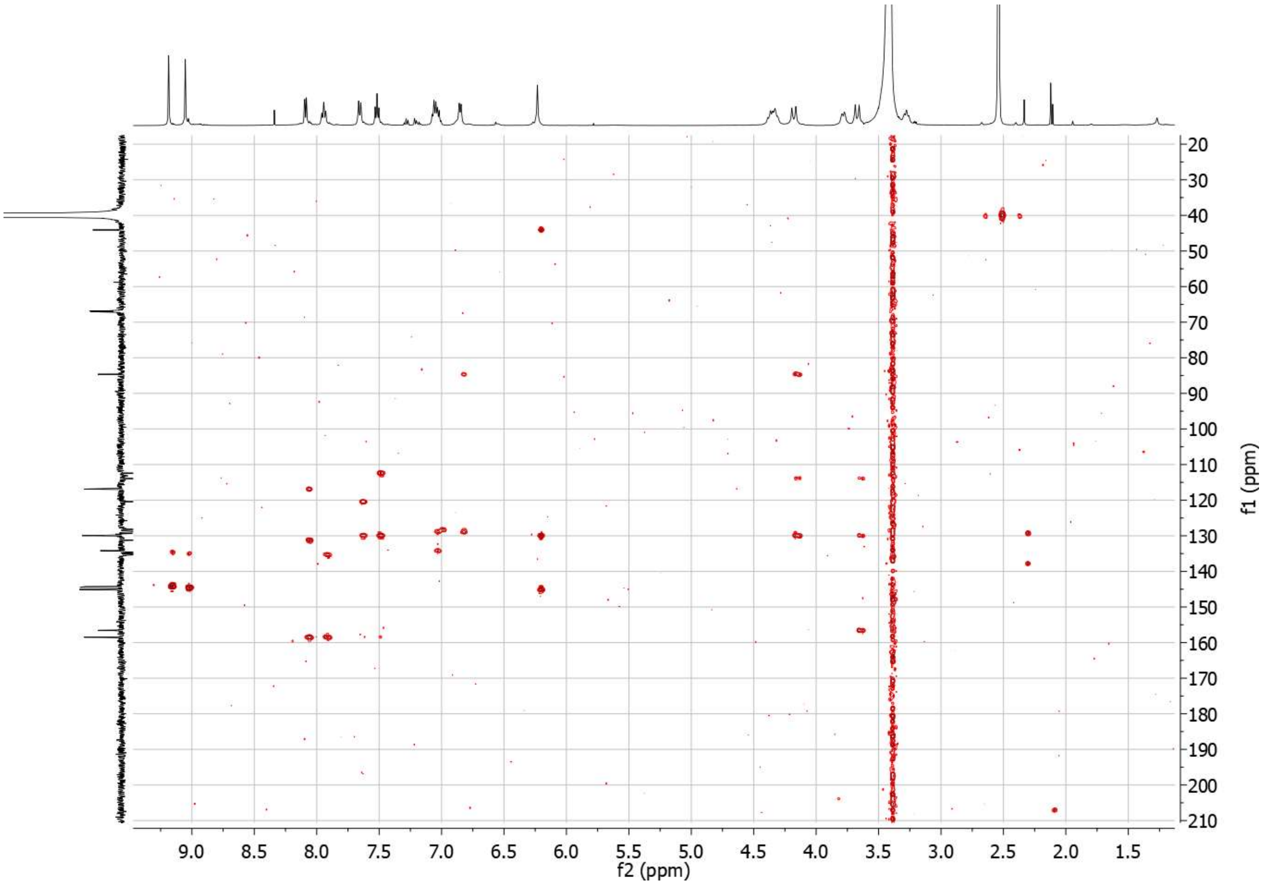

Figure S36 ^{1}H-^{13}C HMBC NMR (DMSO-d6, 125 MHz) spectrum of **CoC-PF₆** (c = 1.4 mM).

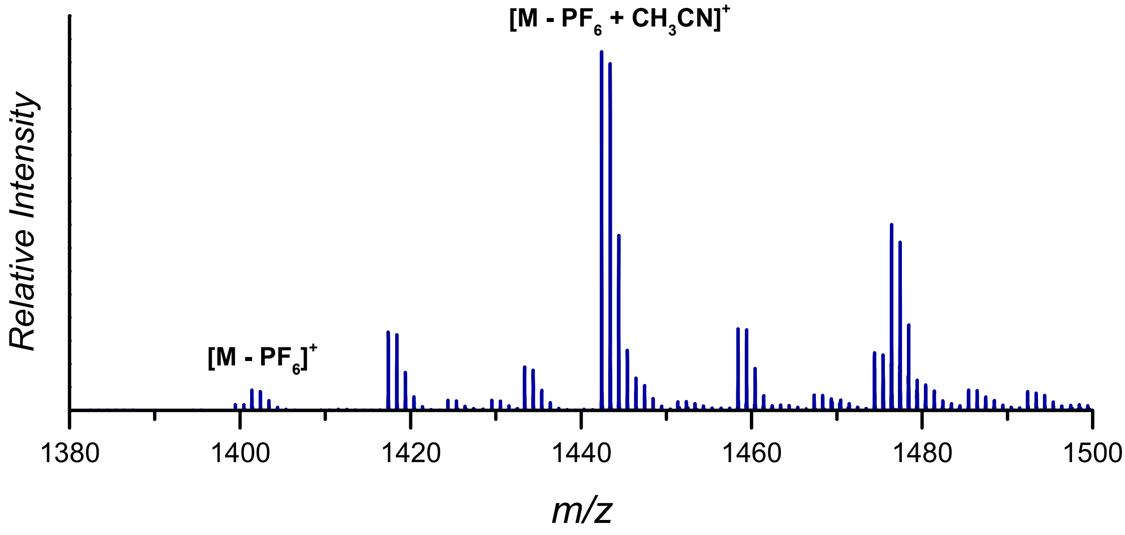

Figure S37 High resolution mass spectrum of **CoC-PF₆** (c = 4 µM, CH_3CN).

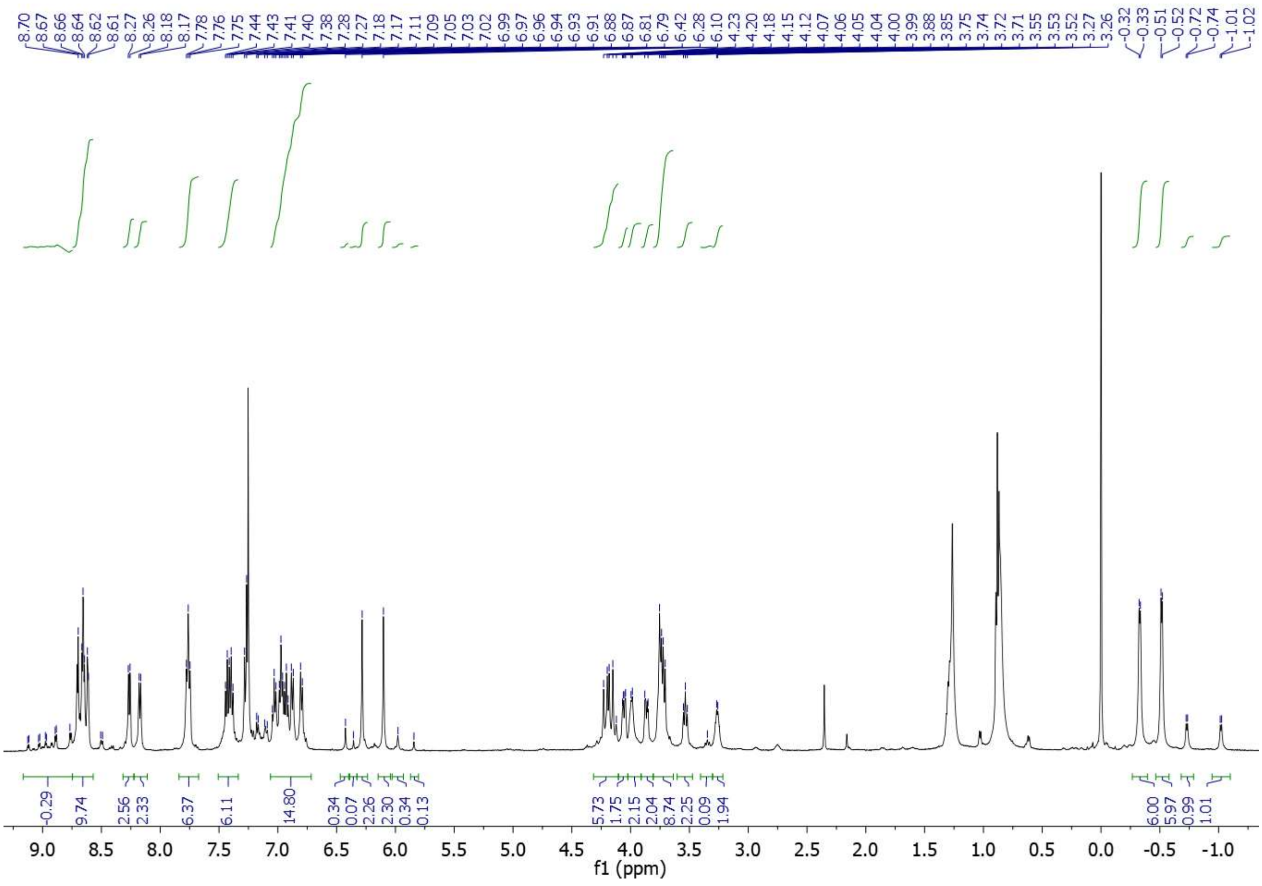

Figure S38 ^{1}H NMR ($CDCl_3$, 500 MHz) spectrum of **CoC*-Cl** (c = 9.6 mM).

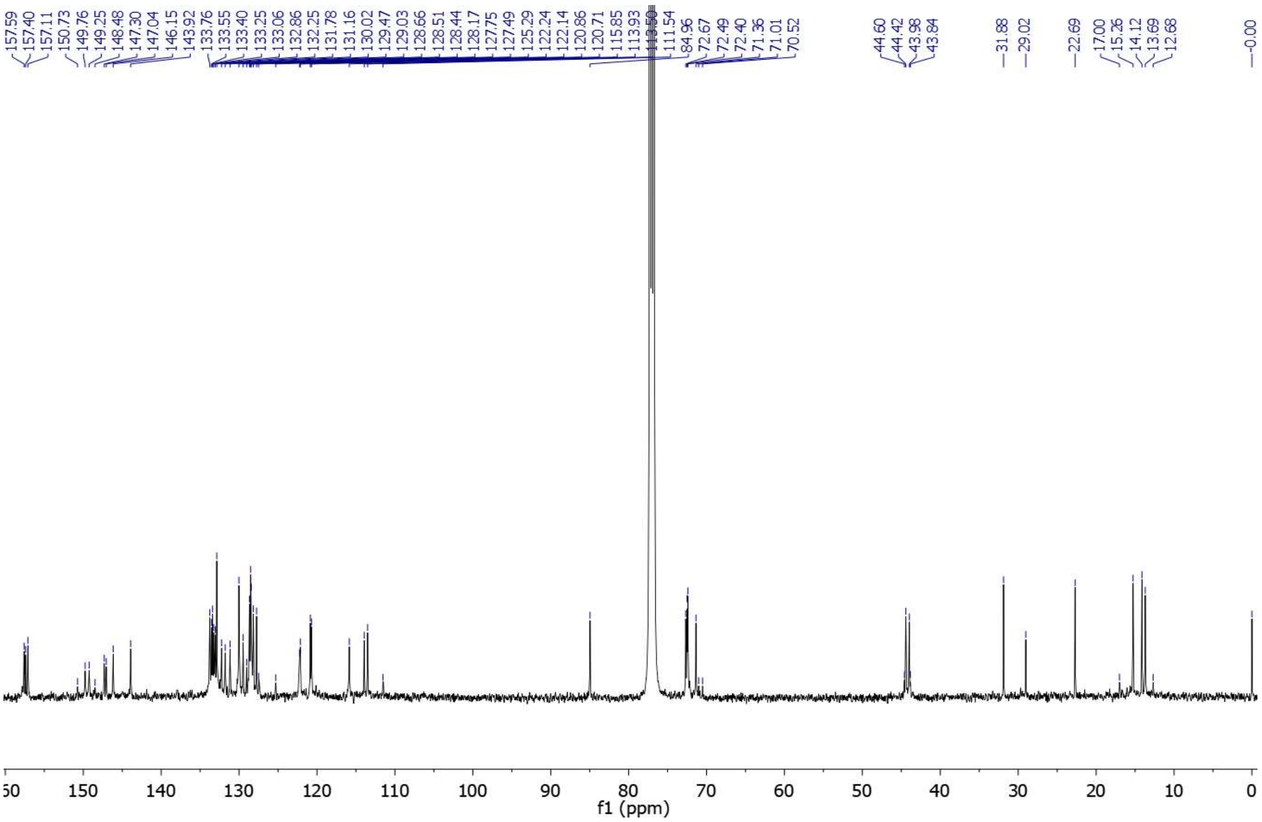

Figure S39 $^{13}C\{^{1}H\}$ NMR ($CDCl_3$, 125 MHz) spectrum of **CoC*-Cl** (c = 9.6 mM).

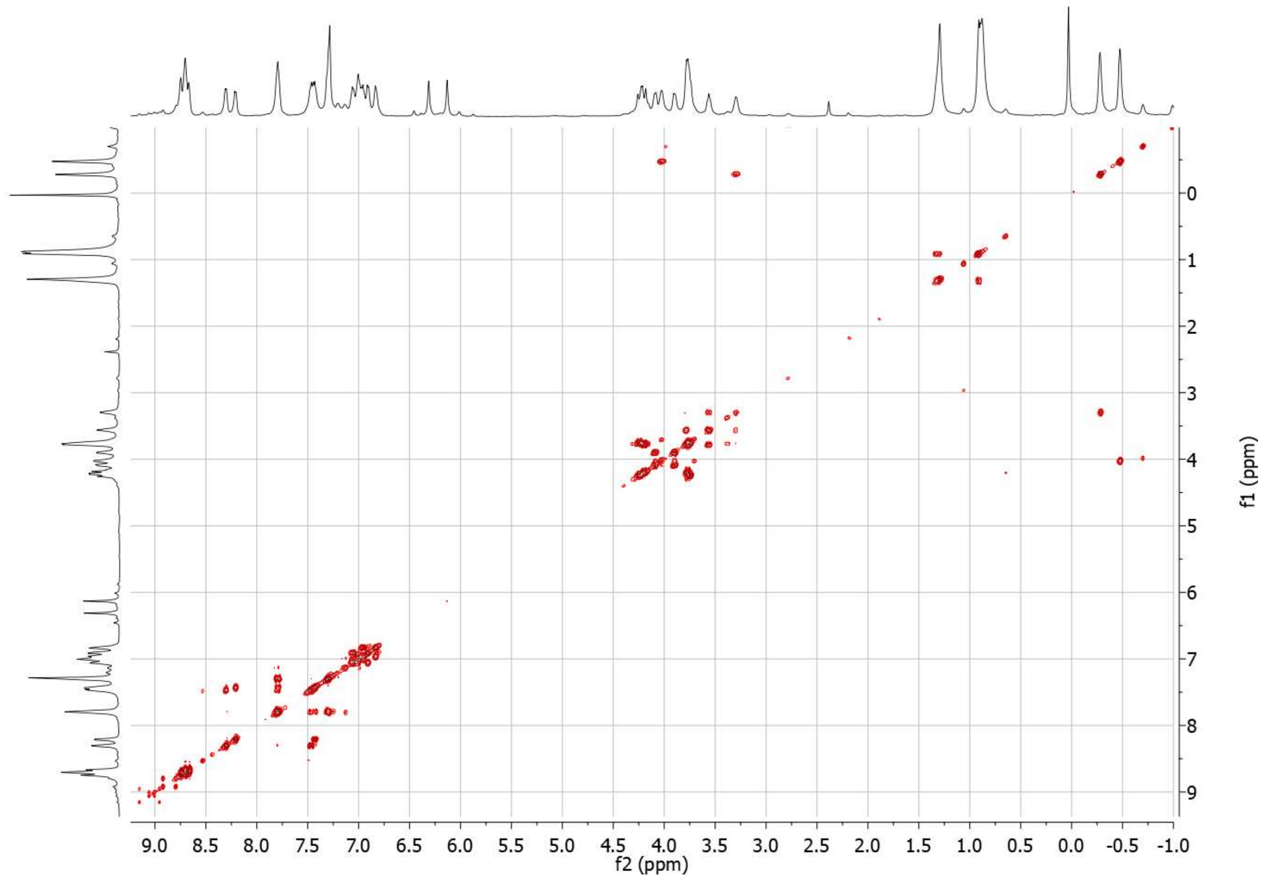

Figure S40 ^1H-^1H COSY NMR ($CDCl_3$, 500 MHz) spectrum of **CoC*-Cl** (c = 9.6 mM).

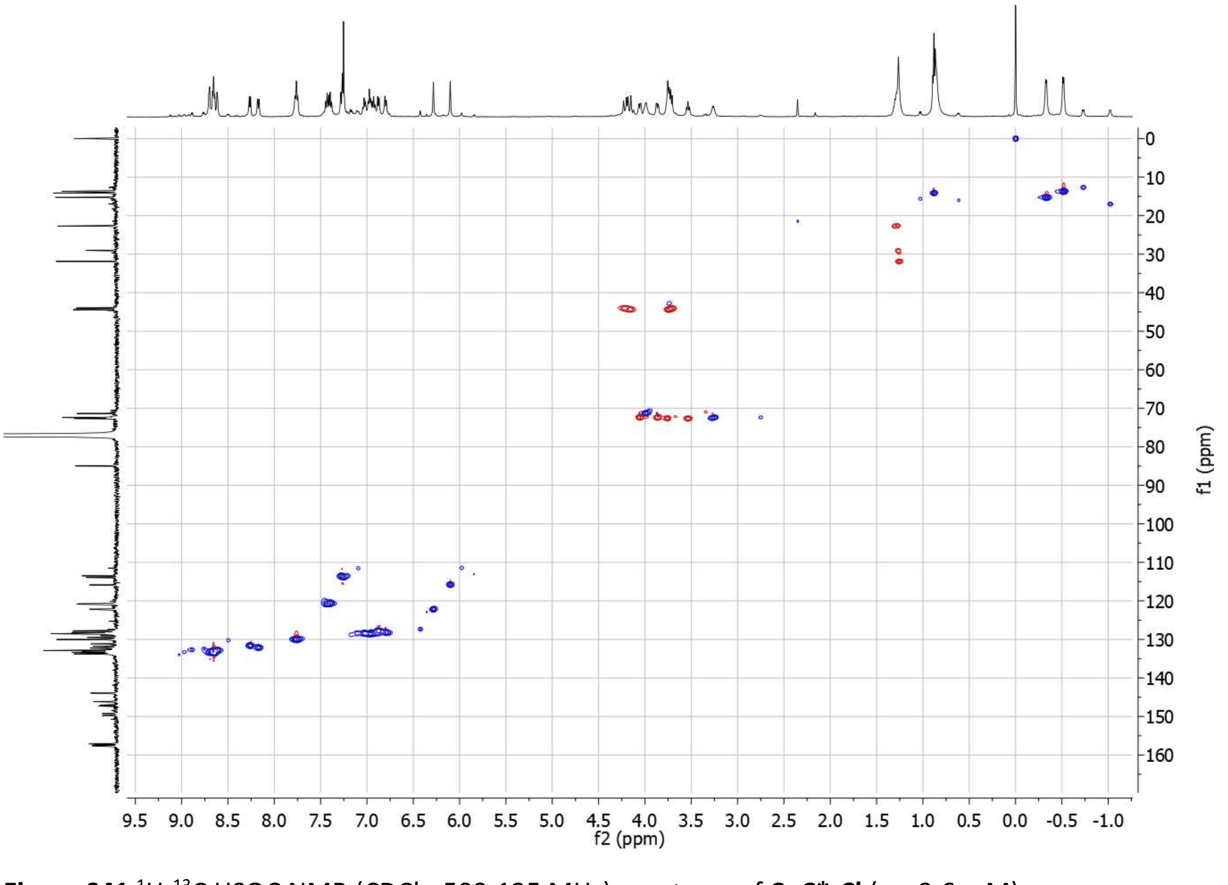

Figure S41 ^1H-^{13}C HSQC NMR ($CDCl_3$, 500-125 MHz) spectrum of **CoC*-Cl** (c = 9.6 mM).

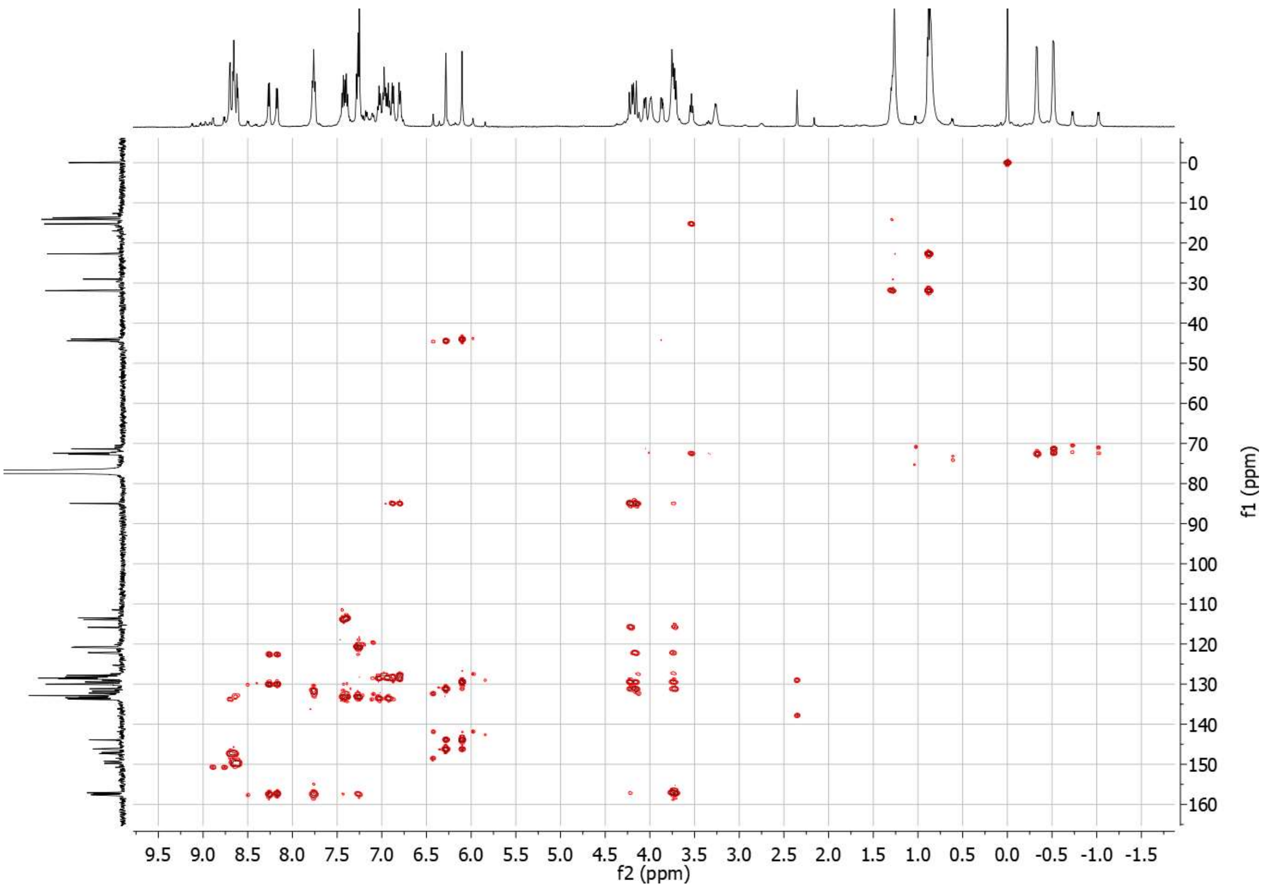

Figure S42 ^{1}H-^{13}C HMBC NMR ($CDCl_3$, 500-125 MHz) spectrum of **CoC*-Cl** (c = 9.6 mM).

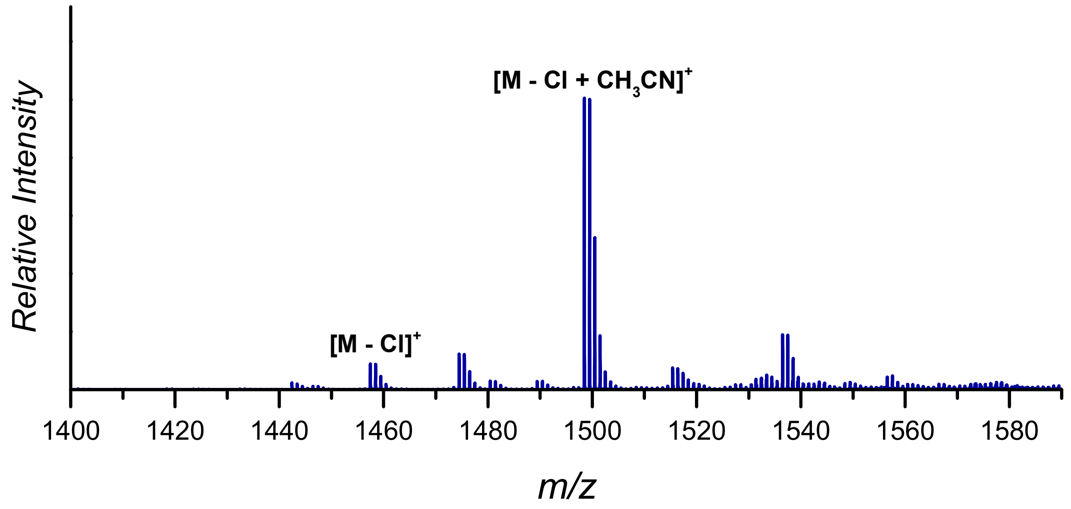

Figure S43 High resolution mass spectrum of **CoC*-Cl** (c = 4 µM, CH_3CN).

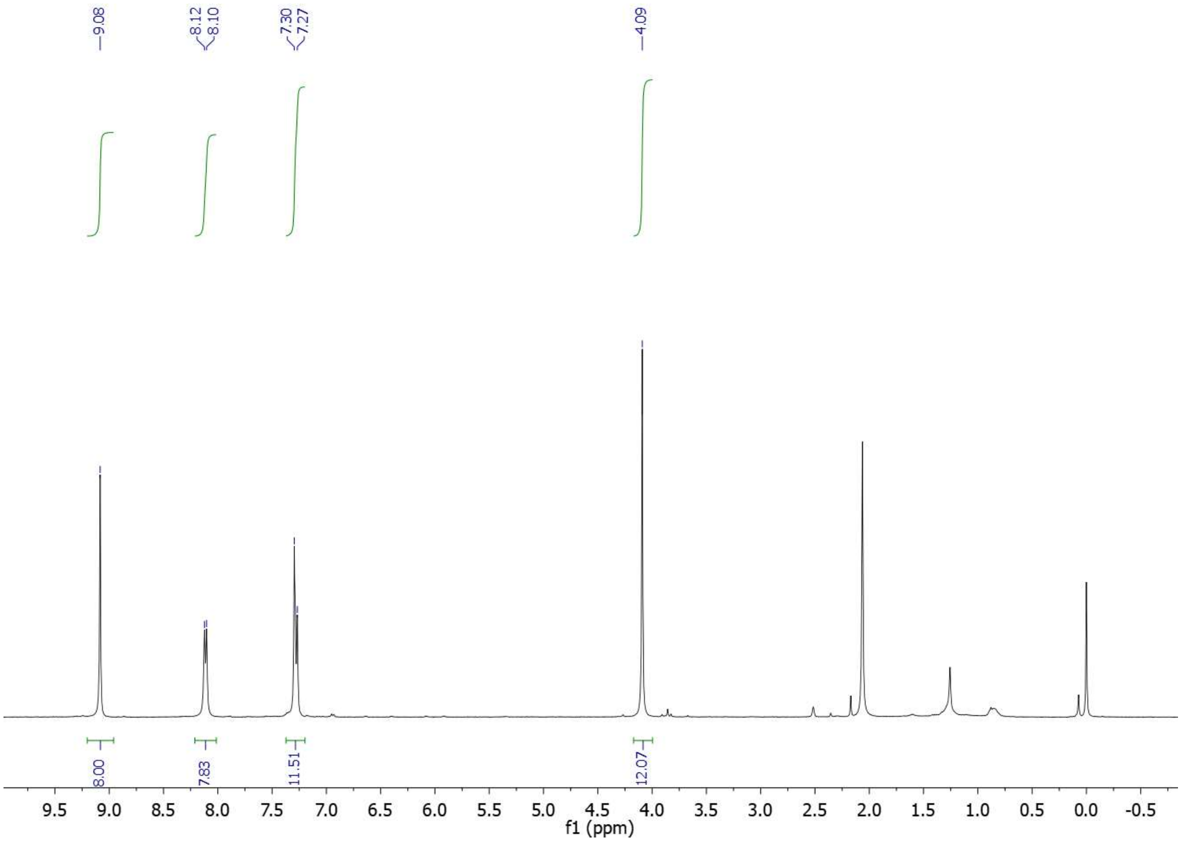

Figure S44 ^{1}H NMR ($CDCl_3$/DMSO-d6 95:5 (v/v), 400 MHz) spectrum of **CoTP-Cl** (c = 1.7 mM).

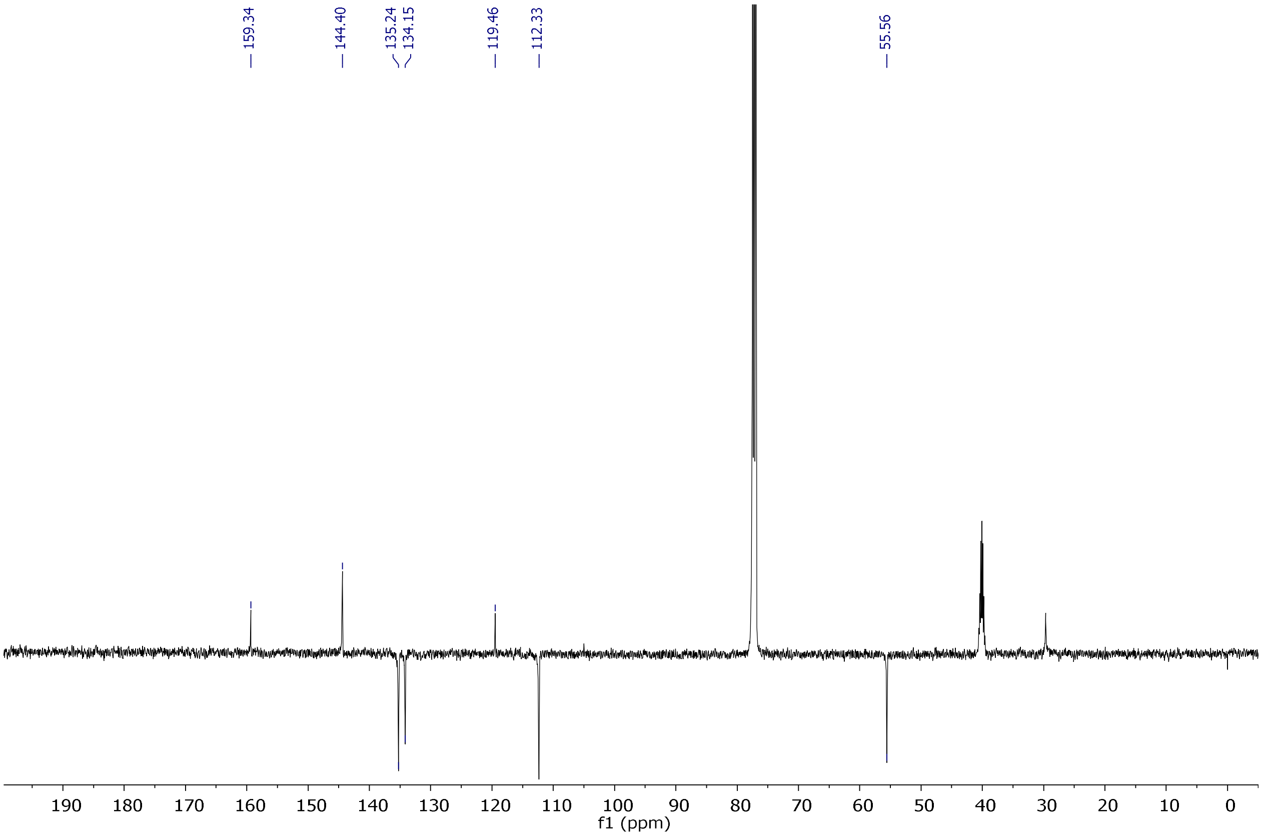

Figure S45 $^{13}C\{^{1}H\}$ NMR ($CDCl_3$/DMSO-d6 95:5 (v/v), 125 MHz) spectrum of **CoTP-Cl** (c = 1.7 mM).

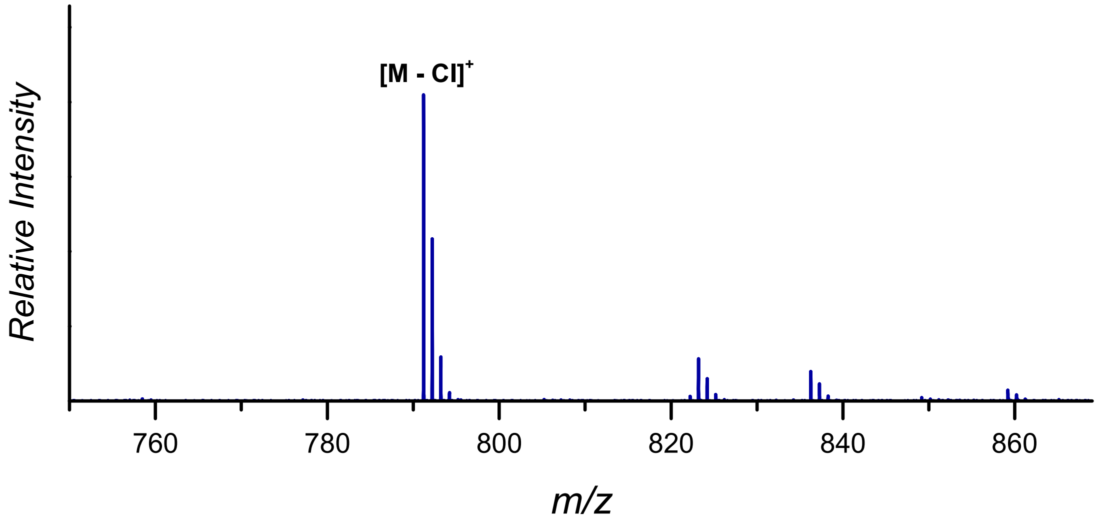

Figure S46 High resolution mass spectrum of **CoTP-Cl** (c = 4μM, $CHCl_3$).

Kinetic model

We built a kinetic model to describe the exchange of pyridine ligands bound at a particular site on the CoC complex in a DRL experiment, with X being any trans-axial ligand bound to the cage complex in solution:

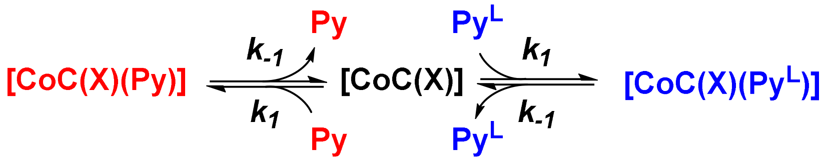

By renaming [CoC(X)(Py)] as [P], and [CoC(X)(Py^L)] as [Y], the initial concentrations of CoC, pyridine (Py) and pyridine-D5 (Py^L) can be written as follows:

$$C_{CoC}^0 = [P] + [Y] + [CoC] \text{ (Equation S3)}$$

$$C_{Py}^0 = [Py] + [P] \text{ (Equation S4)}$$

$$C_{PyL}^0 = [Py^L] + [Y] \text{ (Equation S5)}$$

The time evolution of the concentrations of P and Y can be written according to Equations S6 and S7:

$$\frac{\partial[P]}{\partial t} = k_1[CoC][Py] - k_{-1}[P] \text{ (Equation S6)}$$

$$\frac{\partial[Y]}{\partial t} = k_1[CoC][Py^L] - k_{-1}[Y] \text{ (Equation S7)}$$

By inserting of Equations S3 and S4 into Equation S6, the following differential equation can be obtained:

$$\frac{\partial[P]}{\partial t} = k_1 C_{CoC}^0 C_{Py}^0 - k_1 C_{CoC}^0[P] - k_1 C_{Py}^0[P] + k_1[P]^2 - k_1 C_{Py}^0[Y] + k_1[P][Y] - k_{-1}[P]$$
(Equation S8)

Similarly, Equation S9 can be obtained by insertion of Equations S3 and S5 into Equation S7:

$$\frac{\partial[Y]}{\partial t} = k_1 C_{CoC}^0 C_{PyL}^0 - k_1 C_{CoC}^0[Y] - k_1 C_{PyL}^0[P] + k_1[Y]^2 - k_1 C_{PyL}^0[Y] + k_1[P][Y] - k_{-1}[Y]$$
(Equation S9)

These equations can be simplified by considering a large excess of pyridine(-D5). For large excess of pyridine, [P] becomes negligible compared to [Py] and Equation S4 can be rewritten as:

$$C_{Py}^0 = [Py] \text{ (Equation S10)}$$

By insertion of Equations S3 and S10 in Equation S6:

$$\frac{\partial[P]}{\partial t} = k_1 C_{CoC}^0 C_{Py}^0 - k_1 C_{Py}^0 [P] - k_1 C_{Py}^0 [Y] - k_{-1}[P] \text{ (Equation S11)}$$

Since we consider large excess of pyridine, we assume that the concentration of empty cage complexes in solution will be negligible compared to [P] and [Y] and we can rewrite Equation S3 by:

$$C_{CoC}^0 = [P] + [Y] \text{ (Equation S12)}$$

Equation S11 then becomes:

$$\frac{\partial[P]}{\partial t} = -k_{-1}[P] \text{ (Equation S13)}$$

For large excess of pyridine, fitting the decay of [P] with first order kinetic laws (Equation S1) yields the rate constant of ligand dissociation k_{-1}.

Solving the kinetic model for small excess of ligand:

The analytical solving of Equations S8 and S9 proved to be difficult. We thus decided to solve them numerically using Matlab's ode45 solver. The numerical simulations allow to evaluate the effect of systematic variations of the rate constants k_1 and k_{-1}, and initial concentrations of cage complex or pyridine(-D5).

However, finding initial conditions for the numerical solving of Equations S8 and S9 requires to determine the initial values $[P]_0$, $[Y]_0$ and $[CoC]_0$ at the addition of pyridine-D5. These initial values may depend on the delay between the addition of pyridine and pyridine-D5. For this purpose, we first numerically solve Equations S14, S15 and S16 for the following equilibrium:

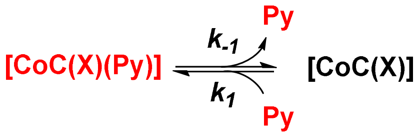

$$\frac{\partial[P]}{\partial t} = k_1[CoC][Py] - k_{-1}[P] \text{ (Equation S14)}$$

$$\frac{\partial[Py]}{\partial t} = k_{-1}[P] - k_1[CoC][Py] \text{ (Equation S15)}$$

$$\frac{\partial[CoC]}{\partial t} = k_{-1}[P] - k_1[CoC][Py] \text{ (Equation S16)}$$

DRL experiments performed with small excess of pyridine ligand and different delays (30s to 5 min – **Figure S5**) revealed that different delays between the addition of pyridine and pyridine-D5 do not produce significantly/statistically different data. The initial equilibrium between the CoC complex and pyridine ligands thus reaches steady state within 30s. Using the k_{-1} rate constant measured with large excess of ligand and the initial concentration of CoC complex, numerical solving of Equations S14, 15 and 16 shows that association rate constants larger than 50 000 M^{-1} s^{-1} are required to reach steady state within 30s (**Figure S47**). These experiments provide a lower range estimation for k_1. Note that we used the k_{-1} value obtained by DRL for measurements on **CoC-Cl** with 200 equiv. pyridine(-D5), *i.e.*, 5.4×10^{-4} s^{-1}.

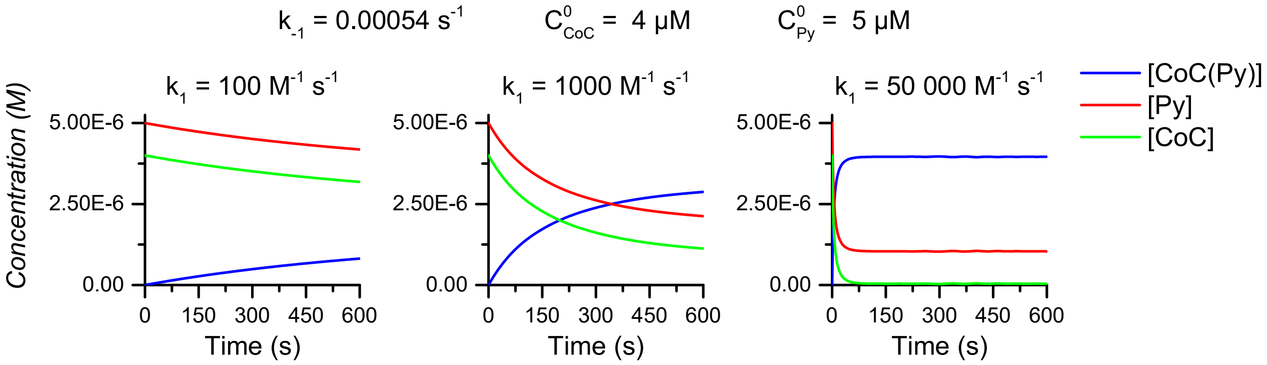

Figure S47. Concentration of [CoC(Py)], [Py] and [CoC] obtained by numerical solving of Equations S14, 15 and 16 with variable k_1 and fixed values of k_{-1} = 0.00054 s^{-1}, C_{CoC}^0 = 4 µM and 1.25 equiv. pyridine relative to initial CoC concentration.

We used the steady-state concentrations of [P] and [CoC] as initial conditions for the numerical solving of Equations S8 and S9. The concentrations of [P], [Y] obtained from the solver are then renormalized in the same way as we renormalize the ion currents of $[CoC(Py)]^+$ and $[CoC(Py^L)]^+$ (See **Figure S48**). The normalized concentrations are then fitted using Equations S1 and S2, thereby affording k_{MS}.

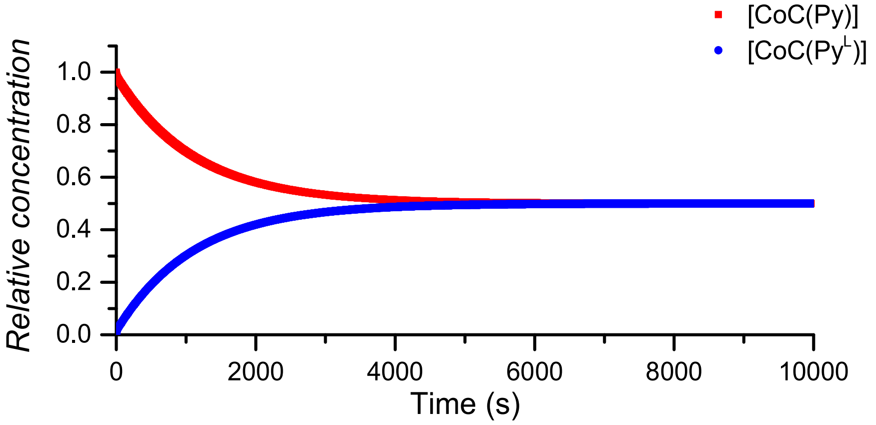

Figure S48. Relative concentrations of [CoC(Py)] and $[CoC(Py^L)]$ obtained by numerical solving of Equations S8 and S9, with k_1 = 50 000 M^{-1} s^{-1}, k_{-1} = 0.00054 s^{-1}, C_{CoC}^0 = 4 µM , 1.25 equiv. pyridine and 1.25 equiv. pyridine-D5 relative to initial CoC concentration.

We then varied systematically one parameter (either k_1, k_{-1} or initial concentration of pyridine(-D5)), while keeping the other ones constant, and monitored the effect on k_{MS}. As shown in **Figure S49**, our model predicts a decrease of k_{MS} with increasing initial concentrations of (pyridine + pyridine-D5). At large excess of (pyridine + pyridine-D5), k_{MS} becomes equal to k_{-1}, in agreement with the approximation discussed in the previous paragraph.

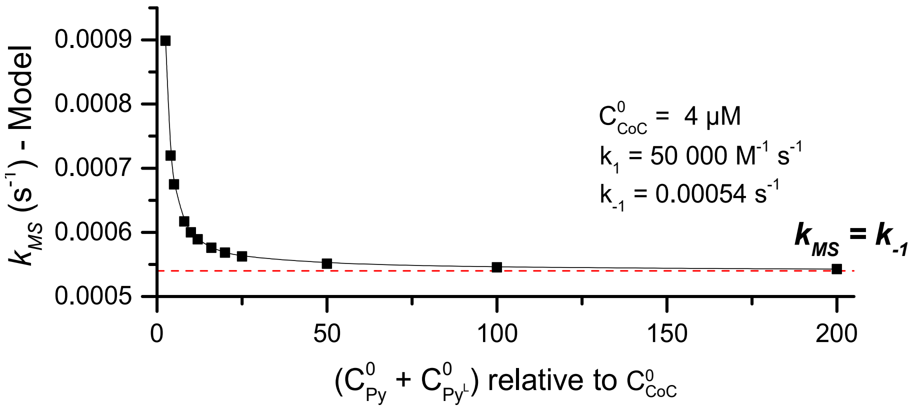

Figure S49. Evolution of k_{MS} with increasing initial concentrations of (pyridine + pyridine-D5) from the numerical solving of the kinetic model. The rate constants k_1 and k_{-1} were kept at 50 000 $M^{-1}s^{-1}$ and 0.00054 s^{-1} respectively.

With large excess of pyridine, k_{MS} is always equal to k_{-1} as evidenced by **Figure S50**, in which we varied k_{-1} while keeping k_1 and initial concentrations of (pyridine + pyridine-D5) constant.

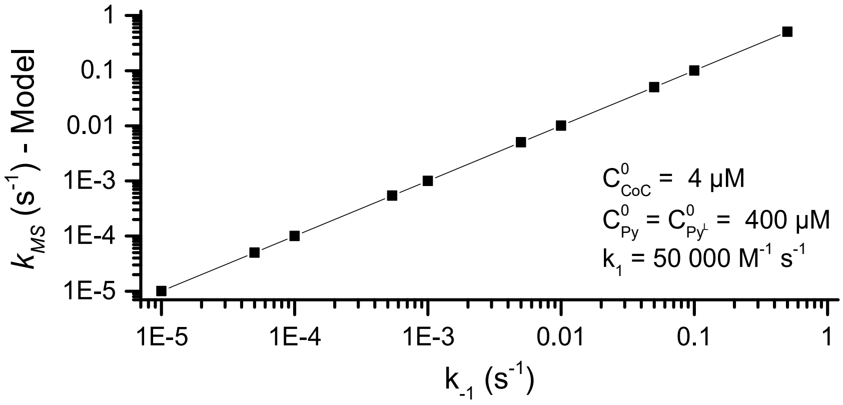

Figure S50. Evolution of k_{MS} with k_{-1} from the numerical solving of the kinetic model. The rate constant k_1 was kept at 50 000 $M^{-1}s^{-1}$ and 100 equiv. of pyridine and pyridine-D5 were considered, relative to the initial concentration of CoC.

However, regardless of the excess of pyridine(-D5), the modelled k_{MS} values are independent from k_1, as shown in **Figure S51**.

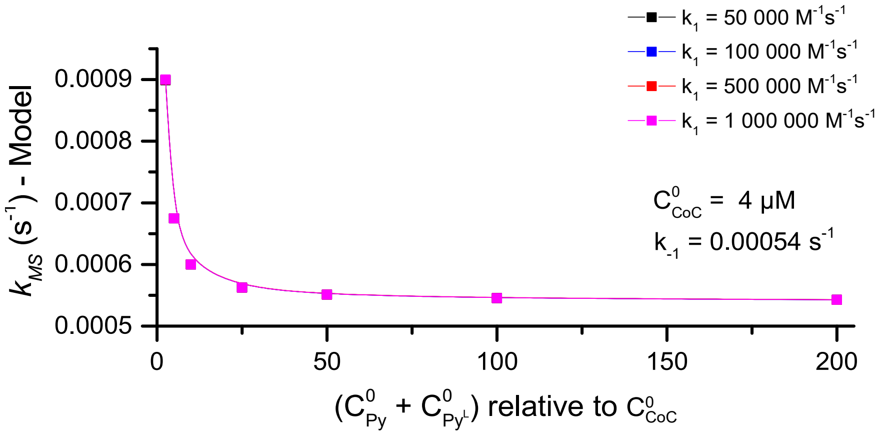

Figure S51. Evolution of k_{MS} with increasing initial concentrations of (pyridine + pyridine-D5) from the numerical solving of the kinetic model. The rate constant k_{-1} was kept at 0.00054 s^{-1} and k_1 was varied from 50 000 $M^{-1}s^{-1}$ to 1 000 000 $M^{-1}s^{-1}$.

Matlab script used to solve Equations S8 and S9:

```
function DRLcage
%Dissociative mechanism - Numerical solving
%Considering free cage
%Definition of the time scale
tstart=0;
tend=10000;
nstep=10000;
tspan=linspace(tstart,tend,nstep);

%options for ode45
options = odeset('RelTol',1e-8,'AbsTol',1e-8);

%Definition of the differential equations to solve for the delay
%equilibration
%z(1) is Cage+Py (P in the paper)
%z(2) is the free pyridine
%z(3) is the free cage
function dzdt = equil(~,z)
    dzdt=zeros(3,1);
    dzdt(1) = k2*z(2)*z(3)-k1*z(1);
    dzdt(2) = k1*z(1)-k2*z(2)*z(3);
    dzdt(3) = k1*z(1)-k2*z(2)*z(3);
    end

%Definition of the differential equations to solve for the DRL step
%x(1) is Cage+Py (P in the paper)
%x(2) is Cage+PyL (Y in the paper)
%x(3) is the free cage
function dxdt = fnct(~,x)
    dxdt=zeros(3,1);
    dxdt(1) = k2*C*P-k2*C*x(1)-k2*P*x(1)+k2*x(1)^2-k2*P*x(2)+k2*x(1)*x(2)-k1*x(1);
    dxdt(2) = k2*C*L-k2*C*x(2)-k2*L*x(1)+k2*x(2)^2-k2*L*x(2)+k2*x(1)*x(2)-k1*x(2);
    dxdt(3) = k1*x(1)+k1*x(2)-k2*P*x(3)+k2*x(3)*x(1)-k2*L*x(3)+k2*x(3)*x(1);
    end

%VALUES TO CHANGE HERE
k1=0.00054; %Kinetic constant k-1 - Dissociation
k2=50000; %Kinetic constant k1 - Association
C=0.000004; %Initial concentration of CoC
delay=1000;


for i=[1.25, 2.5, 5, 12.5, 25, 50, 100] % Iterate for initial concentrations of
pyridine
    P=(i*C); %Initial concentration of Py expressed as a multiple of the inital
cage concentration
    L=P; %Initial concentration of PyL - We use equimolar initial concentrations
of Py and PyL

    %Initial conditions for equilibration ODE solving: [Cage-Py] = (1-initcage),
[Cage-PyL] = 0, [Free cage] = initcage
    zinit=[0;P;C];

    %Solving numerically the initial equilibrium for the set delay
    [t,z]=ode45(@equil,tspan,zinit,options);
    eq1=[z(delay,1)];
    eq2=[z(delay,2)];
```

```
    eq3=[z(delay,3)];
    plot(t,z);
    legend('Cage-Py','Pyridine','Free cage')


    %Initial conditions for DRL ODE solving obtained from solving the
    %equilibration ODEs
    xinit=[eq1;0;eq3];

    %Solving numerically fnct
    [t,x]=ode45(@fnct,tspan,xinit,options);

    %Renormalization of the two datasets obtained by ode45
    y1=[x(:,1)];
    y2=[x(:,2)];
    y3=[x(:,3)];

    z1=(y1)./(y1+y2);
    z2=(y2)./(y1+y2);

    plot(t,z1,t,z2);
    xlabel('Time'); ylabel('Relative abundance');


end
end
```

References

1. SAINT V8.38A. Bruker AXS Inc., Madison, Wisconsin, USA.
2. SADABS-2016/2, Krause, L.; Herbst-Irmer, R.; Sheldrick, G. M.; Stalke, D. *J. Appl. Crystallogr.* **2015**, *48*, 3.
3. Sheldrick, G. M. *Acta Cryst.* **2015**, *A71*, 3.
4. Sheldrick, G. M. *Acta Cryst.* **2015**, *C71*, 3.
5. Gilissen, P. J.; Swartjes, A.; Spierenburg, B.; Bruekers, J. P. J.; Tinnemans, P.; White, P. B.; Rutjes, F. P. J. T.; Nolte, R. J. M.; Elemans, J. A. A. W. *Tetrahedron* **2019**, *75*, 4640.
6. Gilissen, P. J.; Slootbeek, A. D.; Ouyang, J.; Vanthuyne, N.; Bakker, R.; Elemans, J. A. A. W.; Nolte, R. J. M. *Chem. Sci.* **2021**, *12*, 1661.
7. Elemans, J. A. A. W.; Bijsterveld, E. J. A.; Rowan, A. E.; Nolte, R. J. M. *Eur. J. Org. Chem.*, **2007**, 751.
8. Chen, A.; Duez, Q.; Tripodi, G. L.; Gilissen, P. J.; Piperoudis, D.; Tinnemans, P.; Elemans, J. A. A. W.; Roithová, J.; Nolte, R. J. M. *Eur. J. Org. Chem.* **2022**, e202200280
